# Whole blood transcriptome analysis in ewes fed with hemp seed supplemented diet

**DOI:** 10.1038/s41598-019-52712-6

**Published:** 2019-11-07

**Authors:** Marco Iannaccone, Andrea Ianni, Felice Contaldi, Salvatore Esposito, Camillo Martino, Francesca Bennato, Elisabetta De Angelis, Lisa Grotta, Francesco Pomilio, Daniele Giansante, Giuseppe Martino

**Affiliations:** 1 Faculty of Bioscience and Technology for Food, Agriculture, and Environment, University of Teramo, Via R. Balzarini 1, 64100 Teramo, Italy; 2 CREA Research Centre for Vegetable and Ornamental Crops, Via Cavalleggeri 25, 84098 Pontecagnano Faiano, Italy; 3 Istituto Zooprofilattico Sperimentale dell’Abruzzo e del Molise “G. Caporale”, Via Campo Boario, 64100 Teramo, TE Italy

**Keywords:** RNA sequencing, Transcriptomics

## Abstract

The hemp plant (*Cannabis sativa* L.) has a long tradition of being used for many different purposes such as industry, medicine and nutrition. In particular, because hemp seed (HS) is rich in oil protein and considerable amounts of dietary fiber, vitamins and minerals that are particularly suitable also for animal nutrition. Different studies have evaluated HS on qualitative and quantitative properties of livestock products but as of today, nobody has investigated the molecular pathway behind HS supplementation in farm animals. Thus, in this study, we will report the first RNA sequencing of the whole-blood transcriptome of ewes fed either with a controlled diet (CTR, n = 5) or with a diet supplemented with 5% of hemp seed (HSG, n = 5). Applying a false discovery rate (FDR) <0.05 and a log_2_FC either higher than 0.5 or lower than −0.5, we identified 314 differentially regulated genes in the HS-supplemented group compared to the CTR group. Several genes encoding for different subunits belonging to the complex I, II, III, IV and ATP-synthase were up-regulated making oxidative phosphorylation (FDR: 3.05e-19) and thermogenesis (FDR: 2.17e-16) the highest up-regulated pathways in our study. Moreover, we found up-regulation in different genes involved in lactose biosyntheses such as *GALK1* and *PGM1* and, as a result, we observed a statistically higher lactose percentage in the HSG group (p < 0.05). These results indicate that HS supplementation positively affects the energy production pathway in lactating ewes conferring them also more resistance to adverse climatic conditions such as low temperature. Finally, the higher milk lactose content makes the derived dairy products more profitable.

## Introduction

Recently in the field of animal nutrition, people have paid more attention to meal formulations that meet both the consumers’ needs and ameliorated animals’ health. Thus, it is becoming common that animal diets are often supplemented either with micronutrients^1–3^ but also with by-products of agriculture^4,5^. Moreover, there is also more interest in the use of plants rich in secondary compounds. Of these, *Cannabis sativa* L. (hemp) deserves special attention because it has been used for centuries for different purposes such as a source of fiber but also as a medicinal plant thanks to the cannabinoids contained in the leaves and flowers^6^. In the European Union (EU), the cultivation of hemp is permitted only for the varieties where the maximum content of tetrahydrocannabinol (THC), the main psychoactive substance, is limited to 0.2% (w/w) as dry matter^7^. Hemp seed (HS) is generally used for animal nutrition and is considered THC free; however, some studies have reported traces of THC present in the seed sample probably because it was contaminated with plant debris^6^.

HS is rich in oil and proteins and provides a considerable amount of dietary fiber, vitamins and minerals^6^; moreover, the by-product following oil extraction called HS cake can be used as a more economical source of protein^8^. Studies about the use of HS for animal nutrition have mainly focused on the improvement of the quantity and quality of production. In feedlot cattle, different amounts of HS included in the diet did not show any negative effects on either the matter intake or rate or efficiency of gaining weight; however, positive effects on beef tissue quality were recorded because the content of conjugated linoleic acids (CLAs) was relatively higher but, at the same time, saturated fatty acids (SFAs) also increased^9^. According to this study, also in a different trial, HS cake supplementation in comparison to soybean meal favorably affected FAs composition by boosting the ratio of polyunsaturated on monounsaturated fatty acid (PUFA/MUFA)s in both fresh and cooked meat from bovine *M. longissimus dorsi*^10^. HS cake was also evaluated as a source of crude protein in ewes compared to peas and rapeseed cake. While the crude protein amount was similar between all supplements, Karlsoon *et al*.^11^ have shown the metabolized energy produced by HS cake was lower in comparison to the other two supplements probably because the nondegradable rumen component negatively affects intestinal digestibility. Thus, while the HS cake showed controversial results as a protein food source, a HS supplemented diet favorably effects the fatty FA composition in lactating goats, increasing the quality of milk from a human consumer’s point of view^12^. Indeed, HS feeding also showed an increased amount of fat, shifting the composition in favor of PUFAs and in contrast to the lipids that are considered related to hypercholesterolaemic diseases^13^. In agreement with these studies, also using HS oil as a dietary supplement, it was possible to modify the goat milk’s fatty acid quality with a higher ratio of PUFAs on SFAs without affecting liver function^14^. HS supplementation has been used also in broiler and laying hens for assessing affects on meat and egg quality respectively^15,16^. Importantly, in broilers, HS did not affect negatively growth performance but positively influenced the serum lipid profile showing a decrease of triglyceride, low-density lipoproteins (LDL) and total cholesterol. Moreover, it also improved gut health as a consequence of reducing coliform bacteria associated with HS supplementation^15^. In laying hens, both raw and heat-treated HS improved the egg quality, egg FA profile and hen performance values; moreover, the heat treatment modified the flavour of HS restoring the feed consumption at the control group level^16^.

This data encourages the use of hemp plants and its role as a supplement in animal diets. Despite the positive effects on the quality of products, transcriptomic data behind the HS supplementation is lacking. Thus, in this study using a high-throughput RNA-sequencing approach, we elucidate the molecular mechanisms, signaling pathways and metabolic processes behind the hemp seed supplementation in sheep.

## Results

### Influence of HS supplemented diet on whole blood cell count and milk composition

**Figure 1:**
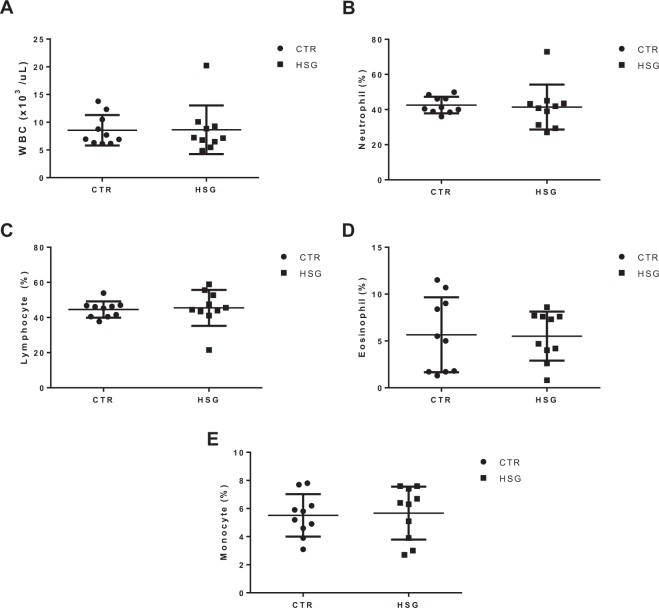
Effect of hemp seed HS-supplemented diet on complete whole blood formula. (**A**) white blood cell, (**B**) neutrophils, (**C**) lymphocyte, (**D**) eosinophil and (**E**) monocyte were measured in CTR and HSG groups at the end of supplementation period. Each point represents a single subject, and data are expressed as percentage. Any possible differences were analyzed by using the Student’s *t*-test. CTR: a control group.

**Figure 2:**
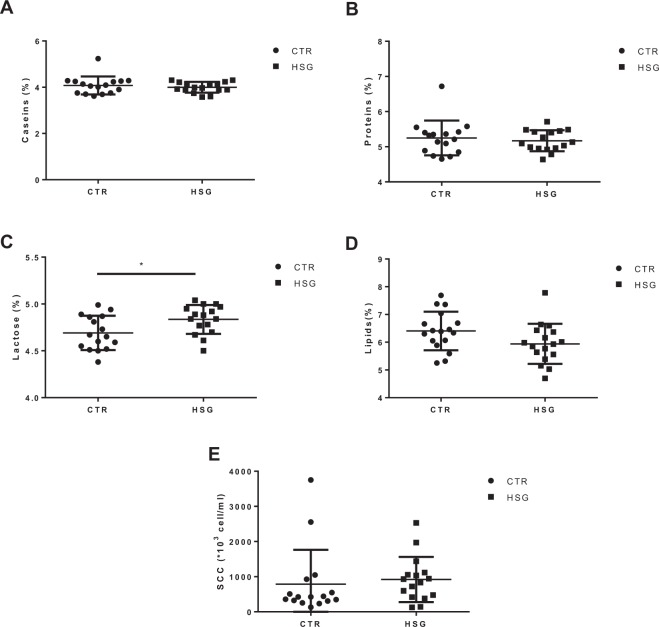
Effect of hemp seed HS-supplemented diet on milk composition. (**A**) caseins, (**B**) proteins, (**C**) lactose, (**D**) lipids and (**E**) somatic cells were quantified in CTR and HSG groups at the end of supplementation period. Each point represents a single subject, and data are expressed as percentage for proteins, caseins, lactose and lipids while somatic cells are expressed as absolute number. Any possible differences were analyzed by using the Student’s *t*-test and a p-value <0.05 is considered significant. *p-value <0.05. CTR: a control group.

To evaluate the overall health of the lactating ewes fed with a 5% HS supplemented diet, the complete whole blood count was evaluated in the control (CTR, n = 10) and HS group (HSG, n = 10) at the end of the supplementation period (day 30). As shown in Fig. 1, no differences were detected either in total white blood cell counts or in the cell type sub-set (neutrophils, lymphocytes, eosinphils and monocytes), indicating that HS does not have a detrimental effect on either innate and adaptive immune system. We assessed also the influence of HS on milk parameters; while total amount of proteins (including caseins) and lipids were not hanged between CTR and HSG groups, lactose showed a statistically significant increase in HSG (4.69 ± 0.04 *vs* 4.84 ± 0.03). Finally, the somatic cell counts were measured as a common parameter used for diagnosis of infectious disease and no differences were found between the two groups indicating that HS is not relevant to the mammary gland health status (Fig. 2).

### Influence of HS supplemented diet on blood transcriptome

In order to identify the transcriptomic signature in lactating ewes fed with HS supplemented diet, we sequenced the RNA isolated from whole blood collected from animals of both groups (CTR, n = 5; HSG, n = 5). Filtering the expression by false discovery rate (FDR) <0.05 combined with a log_2_ fold change (log_2_FC) either higher than 0.5 or lower than −0.5, we identified314 differentially expressed genes (DEGs) (Supplementary Table 1), of which 270 and 44 DEGs were up- and down-regulated, respectively (Supplementary Fig. 1). To identify molecular pathways associated with our data set, we interrogated STRING using the highest available interaction score (0.9) in order to increase stringency and avoid possible uncorrected data interpretation. Up- and down-regulated genes were analyzed separately and no enriched pathways were associated with the down-regulated genes (data not shown). Analyzing the up-regulated genes, in Table 1, we show all the statistically significant enriched pathways; in Supplementary Fig. 2, we show graphically all possible interactions between the up-regulated DEGs and it is possible to appreciate that they mainly clustered in three groups (I, II, and III). The first group includes genes that are shared between oxidative phosphorylation (FDR: 3.05e-19) and thermogenesis (FDR 2.17e-16) pathways; the second group involves gene related to sugar metabolism like Glycolysis/Gluconeogenesis (3.92e-08) and Galactose metabolism (5.72e-06) and more generally to carbon metabolism (5.72e-06); finally, the third group includes genes involved in the protein synthesis and more specifically genes related to the ribosome and protein translation (8.85e-07). Then, we looked for enriched functional GO terms represented in our data set: the significantly enriched biological processes (BP) included genes involved generation of precursor metabolites and energy production (FDR: 3.03e-05), nitrogen compound metabolic process, purine ribonucleoside monophosphate metabolic process, electron transport chain, ATP metabolic process (all significant BP are reported in Supplementary Table 2). In addition, oxidoreductase activity, catalytic activity and NADH dehydrogenase (ubiquinone) activity were the top three enriched molecular functions (MF) GO terms **(**Supplementary Table 3**)**.

**Table 1:** Enriched pathways in the HSG group.

Description	Observed	Background	FDR	Matching protein from the data set
Oxidative phosphorylation (oas00190)	25	119	3.05e-19	ATP5D,ATP5I,ATP6,COX1,COX2,COX4I1,CYC1,CYTB,ENSOARG00000004138,ND4,ND5,ND6,NDUFA11,NDUFA13,NDUFA3,NDUFA7,NDUFA8,NDUFB2,NDUFB7,NDUFS7,NDUFS8,NDUFV1,SDHA,UQCR10,UQCRC1
Thermogenesis (oas04714)	27	211	2.17e-16	ATP5D,ATP5I,ATP6,COX1,COX2,COX4I1,CYC1,CYTB,ENSOARG00000004138,MAPK13,ND4,ND5,ND6,NDUFA11,NDUFA13,NDUFA3,NDUFA7,NDUFA8,NDUFB2,NDUFB7,NDUFS7,NDUFS8,NDUFV1,RPS6KA1,SDHA,UQCR10,UQCRC1
Metabolic pathways (oas01100)	49	1215	3.49e-11	ACSS1,ALDOA,ALPL,APRT,ATP5D,ATP5I,ATP6,B4GALT2,BCAT2,COX1,COX2,COX4I1,CYC1,CYTB,DGKZ,DPM3,ENO1,ENSOARG00000004138,G6PC3,GALK1,GAPDH,HK1,HK3,NADSYN1,ND4,ND5,ND6,NDUFA11,NDUFA13,NDUFA3,NDUFA7,NDUFA8,NDUFB2,NDUFB7,NDUFS7,NDUFS8,NDUFV1,PAFAH1B3,PFKL,PGLS,PGM1,PKM,RRM2B,SDHA,TK1,TPI1,TSTA3,UQCR10,UQCRC1
Retrograde endocannabinoid signaling (oas04723)	16	129	1.08e-09	DAGLB,GNB2,MAPK13,ND4,ND5,ND6,NDUFA11,NDUFA13,NDUFA3,NDUFA7,NDUFA8,NDUFB2,NDUFB7,NDUFS7,NDUFS8,NDUFV1
Glycolysis/Gluconeogenesis (oas00010)	11	62	3.92e-08	ACSS1,ALDOA,ENO1,G6PC3,GAPDH,HK1,HK3,PFKL,PGM1,PKM,TPI1
Ribosome (oas03010)	14	160	8.85e-07	ENSOARG00000005542,ENSOARG00000010831,ENSOARG00000011932,ENSOARG00000017508,ENSOARG00000019510,ENSOARG00000020159,MRPL28,MRPL34,RPL13A,RPL18A,RPL28,RPL8,RPS21,RPS9
Galactose metabolism (oas00052)	7	30	5.72e-06	B4GALT2,G6PC3,GALK1,HK1,HK3,PFKL,PGM1
Carbon metabolism (oas01200)	11	108	5.72e-06	ACSS1,ALDOA,ENO1,GAPDH,HK1,HK3,PFKL,PGLS,PKM,SDHA,TPI1
Fructose and mannose metabolism (oas00051)	6	31	8.95e-05	ALDOA,HK1,HK3,PFKL,TPI1,TSTA3

The most enriched pathways using the up-regulated genes following 30-days hemp seed dietary supplementation.

### Co-expression network analysis

**Figure 3:**
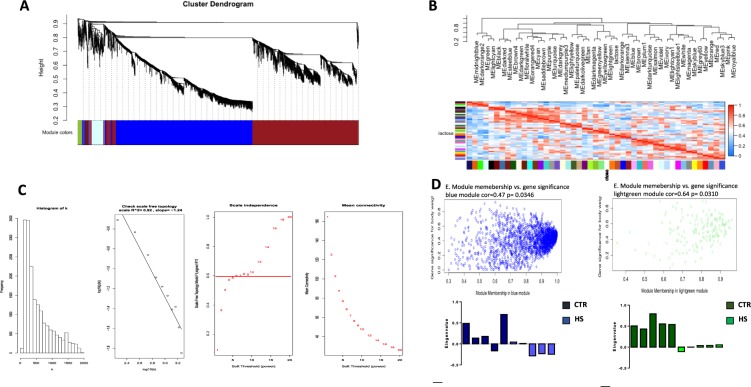
Composite visualization of co-expression analysis. (**A**) Gene dendrogram carried out by average linkage hierarchical clustering. The color row represents the module assignment determined by the Dynamic Tree Cut; (**B**) heatmap plot of the adjacencies in the eigengene network including the trait lactose. Each row and column in the heatmap corresponds to one module eigengene (labeled by color) or trait. In the heatmap, blue color represents low adjacency (negative correlation), while red represents high adjacency (positive correlation). Squares of red color along the diagonal are the meta-modules; (**C**) determination of soft-threshold power in WGCNA. The histogram of connectivity distribution and the scale-free topology are shown in the first two panels, respectively. The scale-free topology index and the mean connectivity for each power value between 1 and 20 are shown in scale independence and mean connectivity panels, respectively; (**D**) a scatterplot of gene significance for weight (GS) versus module membership (MM) in the blue and lighgreen modules. GS and MM exhibit a very significant correlation, implying that hub genes of the brown module also tend to be highly correlated with lactose. Eigene values from both modules (significantly associated with lactose amount) across the ten samples(CTR and HS) are also reported.

Because HS supplementation influences milk lactose probably influencing pathways related to sugar metabolism –mainly Glycolysis/Gluconeogenesis and galactose metabolism- we aimed to identify a consensus network connected with the trait of interest. Thus, using the lactose amount of each of the ten samples as trait for the weighted gene-correlation network analysis (WGCNA), 47 different modules were identified (Fig. 3A). Each module had a different number of genes (Supplementary Table 4), ranging from a minimum of 34 (module brown4) to a maximum of 9.591 (module grey). This latter contained all genes with low expression values, and it was discarded from further analysis. All modules were positively or negatively correlated with lactose, with a correlation value ranging from −0.82 (module lightcyan) to 0.64 (module lightgreen) (Fig. 3B and Supplementary Table 4). Among them, two modules (lightgreen and blue), which include 177 and 2467 genes respectively, showed a correlation coefficient higher than 0.45 and a p-value lower than 0.05 (Supplementary Tables 5 and 6). For both modules, we focused on the genes with the highest intramodular connectivity (hub genes, MM >0.7), as these genes may represent points of biological interest in defining our phenotypes. Among those belonging to the lightgreen module, we identified PGM1 (MM = 0,81; p.value = 0.004), which is strongly associated with galactose metabolism. Furthermore, applying the same criteria to the other significantly associated module (blue), we noticed that GALK1 showed a high MM value (MM = 0.99, p.value = 3.25e-08) and it is also involved in lactose metabolism. Interestingly, about 40% (125 of 316) of our DEGs are represented in the blue module confirming the strengths of our experimental analysis.

As a further confirmation that both light green and blue modules are related to lactose differences between HS and CTR groups, we calculated their eigengene values. As expected, the histogram showed an opposite pattern between the two experimental groups in both modules (Fig. 3D).

## Discussion

The last years have seen an increasing interest in livestock diet supplementation with different ingredients for improving the quality of products and to ameliorate the health status of animals. In this scenario, the use of hemp seed is gaining interest because it is characterized by a high content of oil (26–37.5%), protein (25%), and fiber (28%) with a digestibility of about 20%^6^. Specifically, oil is constituted mostly by PUFA with an approximate ratio of 3:1 between ω-6 and ω-3 fatty acids, which is considered the best condition for human health^17^. Indeed, ω-3 FAs are being widely investigated and a large body of evidence indicates their prominent role as protective lipid mediators in the context of inflammatory, metabolic, neurodegenerative, and neoplastic diseases^18,19^. However, the knowledge around the use of HS in animal nutrition is still limited to tolerability and the effects on production traits^8–16^. Thus, using a RNA-seq approach, which represents one of the most relevant techniques for obtaining a comprehensive analysis of gene expression changes and molecular processes influenced by the diet components, we investigated the effects of an HS-supplemented diet on the whole blood transcriptome of lactating sheep. Whole blood represents a quick and accessible source of biological material. Most importantly, gene expression analysis from this tissue is being successfully used to validate metabolic status from our and different groups^1,2,4,5,20^. However, before assessing the effects of hempseed-supplementation on gene expression, we evaluated the complete whole blood formula in both groups—CTR and HSG—to evaluate the potential effects of HS on cellular subpopulation but did not find any difference (Fig. 1).

HS supplementation influences the expression of 314 genes. About 271 genes were up-regulated, and when we looked for the enriched biological pathways associated to them, the highest one was “oxidative phosphorylation (oas00190)” (Table 1). This upregulation was as result of the genes that are related to all four complexes and ATP-synthase of the electron transport chain (Supplementary Fig. 1). In detail, *ND4*, *ND5*, *ND6*, *NDUFS7*, *NDUFS8*, and *NDUFV1* are subunits that belong to the core of NADH dehydrogenase, and constitute the minimal assembly required for the transfer of electrons from NADH to ubiquinone before they move to the respiratory chain. We also found up-regulation of *NDUFA3*, *NDUFA7*, *NDUFA8*, *NDUFA11*, and *NDUFA13* that belong to subunit A and *NDUFB2* and *NDUFB7* that belong to subunit B; both subunits have accessory functions in the NADH dehydrogenase complex and are not involved in catalytic activity. *SDHA*, the major catalytic subunit of succinate-ubiquinone oxidoreductase, was also up-regulated, which further reinforced the effect of HS supplementation on complex I and II of the electron transport chain. With regards to complex III, we found up-regulated *CYC1*, *CYTB*, *UQCR10*, and *UQCRC1*, while *COX1*, *COX2*, and *COX4I1* were found on complex IV and were also up-regulated. Finally, for the ATP-synthase complex, we found up-regulated *ATP5D*, *ATP5I*, and *ATP6*^21^. However, the protonic gradient generated by electron chain transport is often associated with thermogenesis, which in our data set, is the second most up-regulated pathway. Perinatal mortality caused by hypothermia is one of major concerns within the sheep industry. Thus, the possibility to increase cold tolerance with HS supplementation is desirable. Indeed, it has been previously shown that the high intake of ω-3 fatty acids, together with a ω-6 increase in rectal temperature of newborn lambs can improve cold tolerance^22^. However, in a different study, ewes fed with a docosahexaenoic acid supplement diet at late gestation and early lactation do not ameliorate thermogenesis indexes in lambs but have a potential positive effect on the immune system due to a higher level of immunoglobulins in the colostrum^23^. Thus, because in our study we show a general upregulation of genes involved in the thermogenesis activation, we could speculate that HS supplementation confers a better adaptation to cold stress.

Glycolysis and galactose metabolism pathways are also overexpressed following HS diet Supplementation (Table 1). Specifically, we found upregulation in *GALK1* that catalyze the phosphorylation of galactose, which is one of the steps involved in the production of lactose. Moreover, *PGM1* that together UGP2 catalyze the conversion of glucose to UDP-galactose is upregulated. Because it has been demonstrated that expression of *PGM1* is strongly correlated with the amount of lactose in milk^24^, in agreement, we found a higher content of lactose in animals that were fed hemp seed. Moreover, we performed a co-expression analysis that was successfully used in several studies, including those for candidate biomarkers about boar taint in non-castrated pigs^25^ and identification of molecular signatures related to feed effiency^26^. When applying the WGCNA analysis to our dataset, we found that *GALK1* and *PGM1* belong in the blue and lightgreen cluster, which represent the only two clusters that are statistically correlated to the lactose level. Moreover, several genes that are differentially expressed between our experimental groups belong to those two modules, further supporting our findings.

In conclusion, in this study, we have assessed the transcriptome signature induced by 5% hemp seed supplemented diet in ewes. The findings suggest that pathways related to energy production were the most affected. In addition, we found that this condition could also be potentially beneficial for adaptation to low temperatures. Moreover, we found a higher content of lactose, which makes the derived dairy products more profitable.

## Materials and Methods

### Experimental design and diets

In this study none of the animals was sacrificed. All the experiments were planned and conducted according to Directive 2010/63/EU of the European Parliament (European Union, 2010) and Directive 86/609/EEC (European Economic Community, 1986), which deal with the protection of animals used for scientific purposes.

Thirty two half-bred ewes, homogeneous for age (3–5 years), for days in milking (74 ± 4 days) and for weight (59 ± 4 kg) have been randomly divided into two groups of sixteen ewes each: a control group (CTR) and an experimental group (HSG) whose diet was supplemented with the 5% hemp seeds. To verify eventual variations among the selected groups before the trial, individual milk samples were collected to obtain information about milk yield, chemical-nutritional composition and fatty acid profile. The ewes were housed for the entire trial period in two separate areas of free housing with an access to an identical feeding area with individual pens (0.5 × 1.2 × 1 m) and free access to clean drinking water. The study was conducted for a period of 30 days, in which all animals received total mixed rations (TMR) whose composition, reported in Table 2, was defined taking into account the parameters reported on the Nutrient Requirements of Small Ruminants (Board on Agriculture, Division on Earth, & Life Studies; 2007). Samples of TMR were analyzed, according to AOAC methods (1990)^27^, for crude protein (CD; method 930.15) and ether extract (EE; method 920.39); detergent procedures reported by Van Soest *et al*.^28^ were used for the determination of neutral detergent fiber (NDF) and acid detergent fiber (ADF).

**Table 2:** Ingredients and chemical composition of Total Mixed Rations (TMR) administered to the control group (CTR) and the experimental group (HSG).

	CTR	HSG
**Ingredients (%)**		
Corn	31.1	26.3
Wheat bran	18.5	31.3
Cocoa shells	10	—
Soybean flour	6.2	7.4
Sugar beet pulp	6	6.1
Barley	4	7.9
Sunflower seed flour	5	8.4
Glutinous corn flour	3	3.3
CaCO_3_	1.7	1.7
NaCl	0.5	0.5
NaHCO_3_	0.2	0.2
CaHPO_4_	0.1	0.1
Vitamines	0.5	0.5
Hemp seeds	—	5
**Chemical composition (%)**		
Dry matter (DM)	87.8	87.8
Crude protein^a^	17	17
Ether extract^a^	3.8	3.8
Raw cellulose^a^	8.2	8.8
Ash^a^	7.3	6.5
Starch^a^	27.8	28.2
Neutral Detergent Fiber (NDF)	37.6	36.8
Acid Detergent Fiber (ADF)	23.9	24.2

^a^On a DM basis.

CTR = Control Group; HSG = Experimental Group.

### Blood and milk sampling

Milk, and whole blood (WB) samples were collected from each group at the beginning (T0) and after 30 days (T30) of the supplementation time. Although in the study we have used a larger number of animals, for the RNA-Seq analysis, 2.5 mL of jugular venous blood were collected from 10 animals at T30 (5 from the control group and 5 from HSG). The selection was performed with the aim to have all ewes with the same age (3 years) in order to avoid potential variations induced by this parameter. Duplicate WB samples from each individual animal were collected in PAXgeneTM tubes (Qiagen SpA, Milan, MI, Italy), stored at room temperature overnight and then at −20 °C until RNA isolation, as per the manufacturer’s instructions.

### Blood analysis

Complete blood cell count with leukocyte formula (total white blood cells, monocyte, lymphocyte, basophils, neutrophils, and eosinophils) for both CTR (n = 10) and HSG (n = 10) groups at T0 and T30 were performed at the Veterinary and Public Health Institute (Teramo, Italy) using a laser-based hematology analyzer with software applications for animal species (ADVIA 120 hematology system, Siemens, Munich, Germany) and following the routine procedure of the institute (Veterinary and Public Health Institute “G. Caporale”, Teramo, Italy).

### Chemical analysis of milk

Chemical composition of milk (fat, protein, casein, and lactose) was determined by MilkoScan FT 6000 (Foss Integrator IMT; Foss, Hillerød, Denmark), whereas the somatic cells count (SCC) was performed using the Fossomatic FC (Foss).

### Library preparation and RNA-Seq analysis

Next generation sequencing experiments, comprising RNA extraction and bioinformatics analysis, were performed by an external company (Genomix4life SRL, Baronissi, Salerno, Italy). Total RNA was extracted using TRIzol and following manufacturer’s instruction (Invitrogen, Carlsbad, CA, USA). Indexed libraries were prepared from 300 ng/ea purified RNA with TruSeq Stranded mRNA Sample Prep Kit (Illumina, San Diego, CA, USA) according to the manufacturer’s instructions. Libraries were quantified using the TapeStation 4200 (Agilent Technologies, Santa Clara, CA, USA) and pooled such that each index-tagged sample was present in equimolar amounts, with final concentration of the pooled samples of 2 nM. The pooled samples were subject to cluster generation and sequencing using an Illumina NextSeq. 500 System (Illumina, San Diego, CA, USA) in a 2 × 75 paired end format at a final concentration of 1.8 pmol.

The raw sequence files generated (.fastq files) underwent quality control analysis using FastQC tool Version 0.11.8 using default settings (http://www.bioinformatics.babraham.ac.uk/projects/fastqc/). To remove the adapter sequences, cutadapt (version 1.4) was used^29^. The mapping of paired-end reads was performed using STAR (version 2.5)^30^ on reference genome Oar_v3.1 (GCA_000298735.1) from Ensembl (http://www.ensembl.org/Ovis_aries/Info/Index). The quantification of transcripts expressed for each replicate of the sequenced samples was performed using HTSeq-Count algorithm DESeq. 2^31^ was used to perform the differential expression analysis. Genes with a false discovery rate (FDR) less than 0.05 were considered as differentially expressed genes (DEGs). Raw data associated with this project are deposited in the GenBank’s Sequence Read Archive (SRA) under the accession Bioproject number PRJNA528905.

### Enriched pathway analysis

The STRING software (v 11 http://string-db.org/) was used to identify canonical pathways using the dataset of 314 DEGs identified between HSG group and the CTR one with a false discovery rate (FDR) <0.05. We set the interaction score as 0.9, the highest value permitted by the software to avoid false positive. The significance of the canonical pathway was measured with the p-value and the ratio of DEG/number of genes in the pathway

### Unsigned weighted correlation networks analysis (WGCNA)

We used the unsigned WGCNA^32,33^ to perform the hierarchical clustering and identify co-expressed genes (“hub genes”), which may have main regulatory functions and/or major impact on our candidate genes. In detail, an adjacency matrix was first created between pairs of all expression values, before to be replaced with the weighted adjacency matrix obtained by raising the correlations to the power of 7 (Fig. 3C). The weighted adjacency matrix was then transformed into a topological overlap matrix (TOM), which allow the calculation of dissimilarity values used to minimize effects of spurious associations. The result was used as input for the linkage hierarchical clustering and the modules (clusters of highly interconnected genes) were identified in the resulting dendrogram through a dynamic hybrid tree cutting algorithm (DynamicTreeCut algorithm).

Using the module eigengene values, we finally estimated the relationships between each module and the lactose amount of each one of the ten samples used in the RNAseq experiment by calculating the Pearson’s correlation values. Modules were filtered out using a correlation value >0.45 and p.value <0.05. Interesting modules were in deep investigated.

### Statistics

GraphPad Prism (GraphPad v.6 Software, La Jolla, CA, USA) was used for statistical analysis. Differences in cell subsets from peripheral blood and plasma samples were assessed using Student’s t-test.

## Supplementary information

Supplementary information
