# Supplementary material for: Whole blood transcriptome analysis in ewes fed with hemp seed supplemented diet
Source: Sci Rep. 2019 Nov 7;9:16192. doi: 10.1038/s41598-019-52712-6 (PMC6838114; doi:10.1038/s41598-019-52712-6)
Supplement: Supplementary file 1 — Supplementary information [file 41598_2019_52712_MOESM1_ESM.pdf]

Whole blood transcriptome analysis in ewes fed with hemp seed supplemented diet

Marco Iannaccone[1†], Andrea Ianni[1†], Felice Contaldi[2], Salvatore Esposito[2], Camillo Martino[3], Francesca Bennato[1], Elisabetta De Angelis[1], Lisa Grotta[1], Francesco Pomilio[3], Daniele Giansante[3], Giuseppe Martino[1,*]

[1]Faculty of Bioscience and Technology for Food, Agriculture, and Environment, University of Teramo, Via R. Balzarini 1, 64100 Teramo, Italy.

[2]CREA, Via Cavalleggeri, 25, 84098 Pontecagnano Salerno, Italy

[3]Istituto Zooprofilattico Sperimentale dell'Abruzzo e del Molise "G. Caporale" Via Campo Boario – 64100 Teramo (TE), Italy

[†]These authors contributed equally to this work

*Corresponding author. gmartino@unite.it (G. Martino).

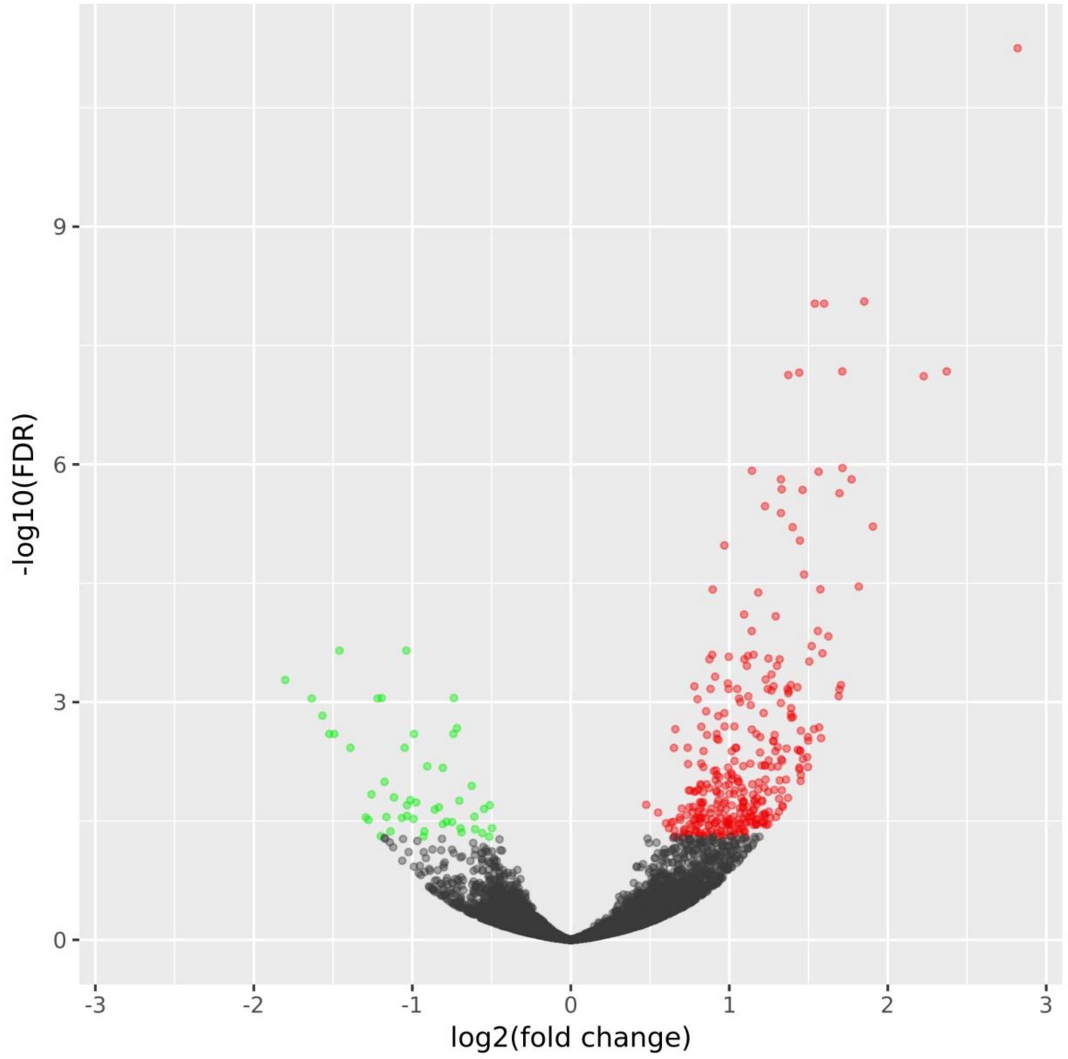

Supplementary figure 1. Volcano plot chart. The Volcano plot shows the relationship between the fold-change (on the X-axis) and the significance of the differential expression test (Y-axis) for each gene in the genome. Black dots represent the genes that are not significantly differentially expressed, while red and green dots are the genes that are significantly UP- and DOWN-regulated, respectively.

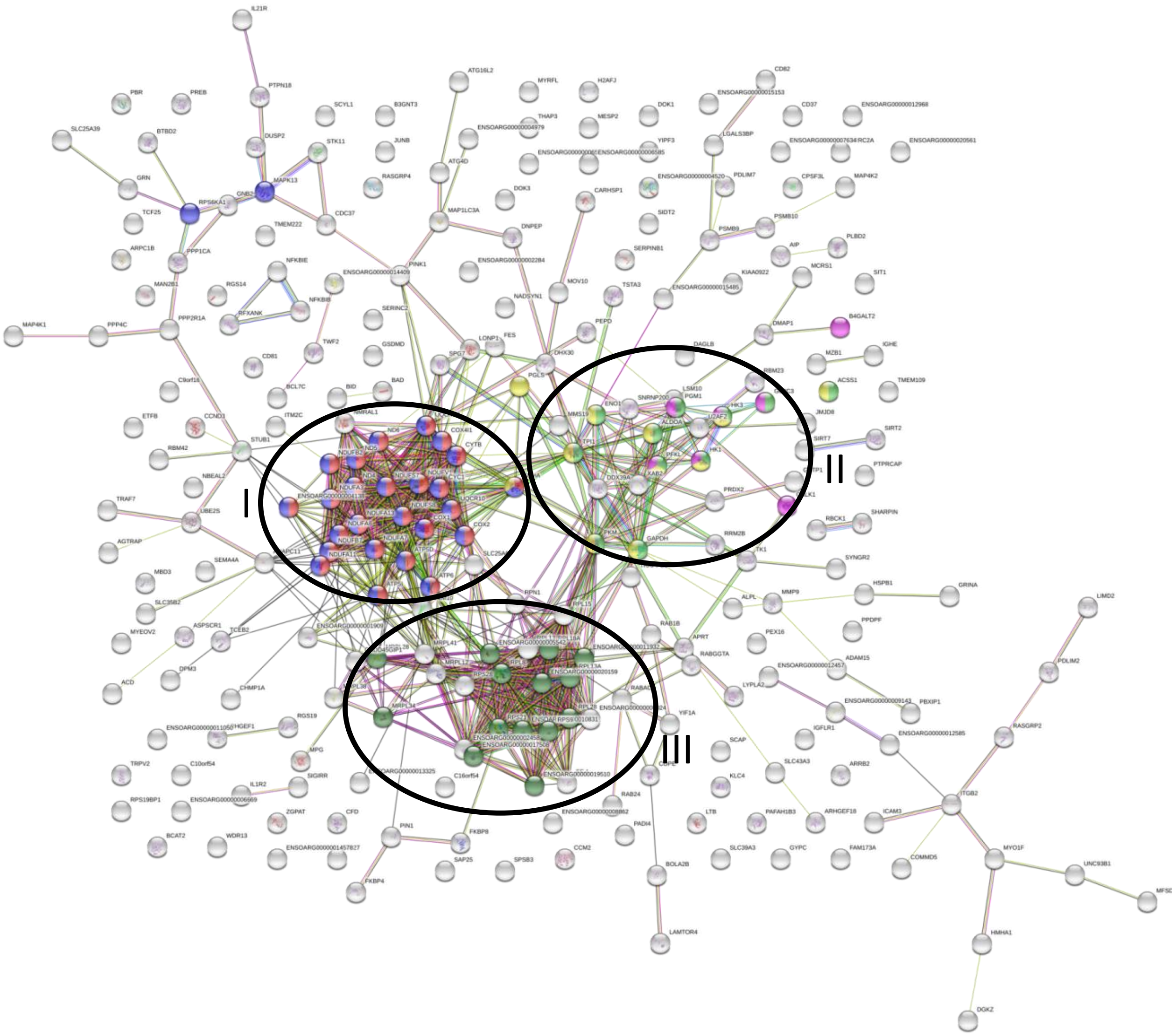

Supplemetary figure 1. Protein-protein interaction generated using the 270 up-regulated genes at the end of supplementation time with HS. DEGs clusterize mainly in 3 different groups: I includes genes belonging to oxidative phosphorylation and thermogenesis (blu and red coloured circles); II incluedes genes belonging to glycolysis/gluconeogenesis (3.92e-08), galactose metabolism and carbon metabolism pathways (yellow, light green and violet coloured circles); III includes gene belonging to ribosome activity (dark green coloured circles). Details of all genes included in the 3 groups are reported in table 1.

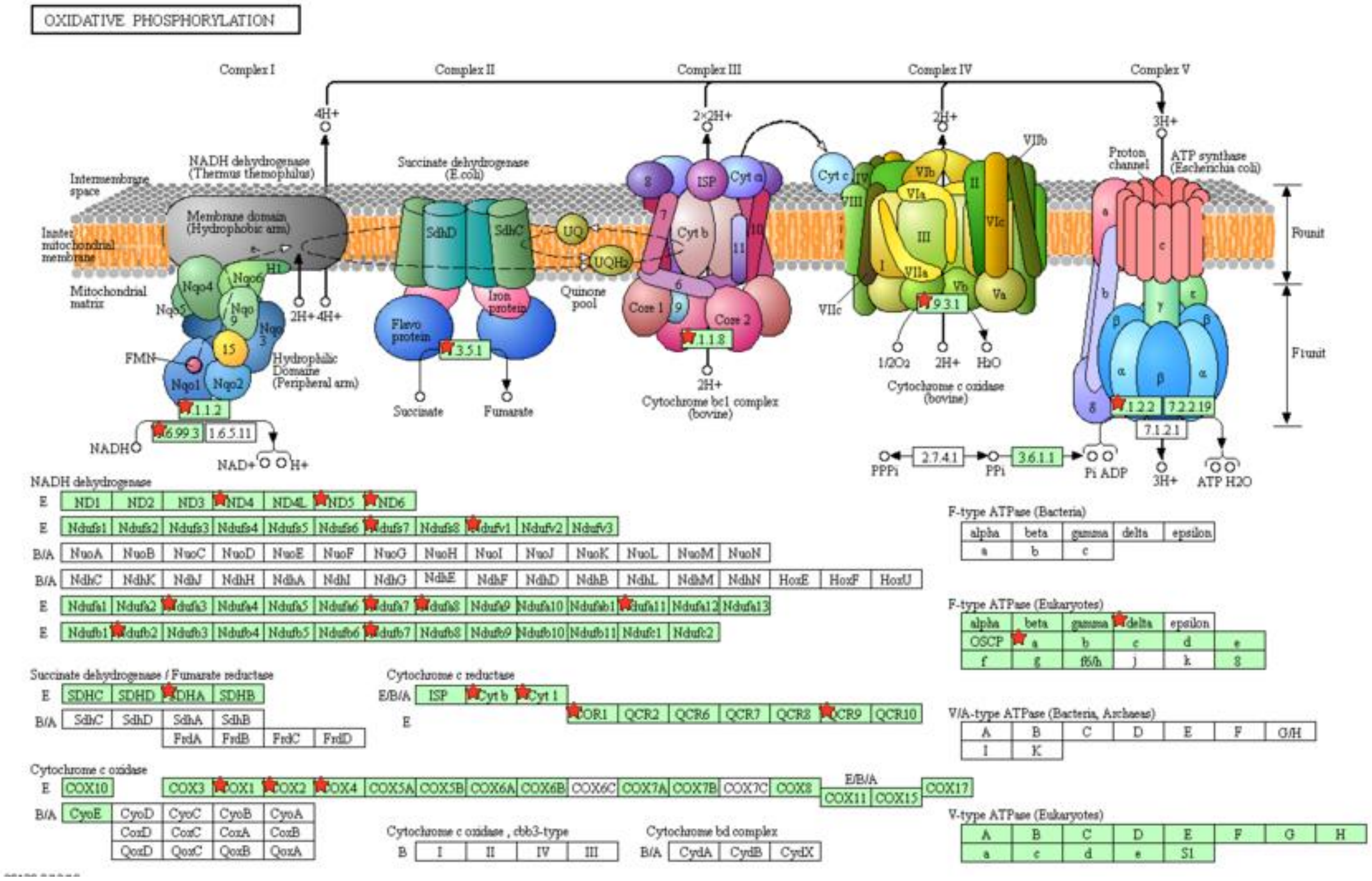

Supplementary figure 1. Schematic diagram of oxidative phosphorylation which includes electron transport chain and ATP synthesis. All genes upregulated by hemp seed supplementation have been indicated with a red star.

Supplementary table 1. List of all differentially expressed genes (FDR<0.05) in the HSG group compared to CTRL group following 30 days of hemp seed supplementation. Down-regulated genes are highlighted in green while up-regulated genes are highlighted in red.

Gene_ID	Gene symbol	log2FC	pvalue	FDR	Chromosome
ENSOARG00000013378	GLIS3	-6.46	8.10E-09	9.52E-06	2
ENSOARG00000020724	MGP	-3.06	1.37E-03	4.94E-02	3
ENSOARG00000015299	LOC101104054	-2.77	8.50E-06	1.64E-03	3
ENSOARG00000002356	RARRES1	-2.70	6.64E-07	2.55E-04	1
ENSOARG00000009775	LOC101123341	-2.58	6.06E-05	6.22E-03	1
ENSOARG00000001914	ABCG2	-2.31	8.98E-06	1.67E-03	6
ENSOARG00000010617	SGIP1	-2.30	2.11E-06	5.94E-04	1
ENSOARG00000001941	LOC101110458	-2.28	3.78E-04	2.17E-02	20
ENSOARG00000014496	CXCL8	-2.07	7.73E-04	3.46E-02	6
ENSOARG00000002463	LXN	-1.88	5.26E-07	2.22E-04	1
ENSOARG00000011975	MOB1B	-1.78	9.61E-06	1.67E-03	6
ENSOARG00000000421	BEX4	-1.75	6.01E-04	2.96E-02	
ENSOARG00000001633	-	-1.73	7.37E-04	3.35E-02	X
ENSOARG00000004954	RAB27B	-1.70	5.54E-04	2.78E-02	23
ENSOARG00000006648	DPM3	-1.68	2.84E-04	1.76E-02	1
ENSOARG00000020985	LOC105613001	-1.53	2.66E-04	1.69E-02	3
ENSOARG00000009306	DOCK7	-1.53	1.86E-04	1.35E-02	1
ENSOARG00000020266	TTF2	-1.47	2.63E-07	1.30E-04	1
ENSOARG00000001101	GNG11	-1.45	4.74E-04	2.54E-02	4
ENSOARG00000004402	DYNLT1	-1.42	2.73E-05	3.42E-03	8
ENSOARG00000021041	C7H15orf48	-1.36	3.50E-04	2.07E-02	7
ENSOARG00000003293	PDGFD	-1.34	1.04E-04	8.87E-03	15
ENSOARG00000023519	-	-1.33	7.93E-04	3.49E-02	7
ENSOARG00000014852	BBS10	-1.33	4.95E-04	2.58E-02	3
ENSOARG00000017989	-	-1.32	1.15E-03	4.40E-02	25
ENSOARG00000019914	-	-1.20	2.30E-06	6.27E-04	3
ENSOARG00000000017	-	-1.20	2.91E-04	1.79E-02	MT
ENSOARG00000019055	NPL	-1.14	5.50E-04	2.78E-02	12
ENSOARG00000009068	RSU1	-1.14	1.09E-03	4.18E-02	13

ENSOARG00000001883	NMD3	-1.07	2.33E-05	3.06E-03	1
ENSOARG00000013732	ARHGAP18	-1.06	8.83E-04	3.67E-02	8
ENSOARG00000006919		-0.95	7.99E-04	3.49E-02	
ENSOARG00000009414	ATP2C1	-0.93	6.32E-04	3.04E-02	1
ENSOARG00000002409	GFM1	-0.91	4.38E-05	4.79E-03	1
ENSOARG00000007240	EFR3A	-0.86	1.35E-03	4.90E-02	9
ENSOARG00000016837	-	-0.85	1.35E-03	4.90E-02	11
ENSOARG00000021027	MYEF2	-0.82	1.26E-03	4.66E-02	7
ENSOARG00000000764	-	-0.80	4.01E-04	2.29E-02	7
ENSOARG00000009351	-	-0.80	4.30E-04	2.39E-02	4
ENSOARG00000008878	CD84	-0.79	1.37E-03	4.94E-02	1
ENSOARG00000015472	C3H12orf29	-0.78	1.26E-03	4.66E-02	3
ENSOARG00000020715	RMND5A	-0.76	4.60E-04	2.47E-02	3
ENSOARG00000020662	DCUN1D1	-0.76	8.72E-04	3.67E-02	1
ENSOARG00000005428	TPI1	0.57	3.47E-04	2.81E-03	3
ENSOARG00000011503	LOC101105179	0.59	4.86E-06	3.28E-04	14
ENSOARG00000000016	COX1	0.65	2.60E-06	2.47E-04	MT
ENSOARG00000013450	NDUFB2	0.66	4.69E-06	3.25E-03	4
ENSOARG00000009826	PGM1	0.72	3.84E-04	2.94E-03	1
ENSOARG00000011318	PPP1CA	0.74	8.70E-04	3.67E-02	21
ENSOARG00000005491	HK1	0.74	2.71E-05	7.36E-04	25
ENSOARG00000005491	RPLP2	0.74	2.71E-05	7.36E-04	21
ENSOARG00000014457	-	0.77	7.16E-04	3.30E-02	5
ENSOARG00000004100	PPP4C	0.80	1.29E-04	1.04E-02	24
ENSOARG00000000035	CYTB	0.81	4.55E-05	9.54E-02	MT
ENSOARG00000000531	ARHGEF18	0.82	1.17E-03	4.43E-02	5
ENSOARG00000019733	MOV10	0.83	4.85E-04	2.57E-02	1
ENSOARG00000011932	RPL18	0.84	4.12E-04	2.31E-02	14
ENSOARG00000000685	B4GALT2	0.85	4.31E-04	3.12E-03	1
ENSOARG00000014386	NDUFA8	0.86	4.97E-05	9.88E-02	3
ENSOARG00000007894	GAPDH	0.90	2.96E-04	2.63E-03	3
ENSOARG00000007797	ARRB2	0.90	3.75E-04	2.17E-02	11
ENSOARG00000005542	LOC101108778	0.90	1.40E-03	5.00E-02	17

ENSOARG00000016615	-	0.91	2.18E-04	1.51E-02	3
ENSOARG00000018886	PARP6	0.93	2.27E-05	6.57E-04	7
ENSOARG00000002728	PEX16	0.94	1.41E-03	5.00E-02	15
ENSOARG00000004384	RPS6KA1	0.94	7.96E-04	3.49E-02	2
ENSOARG00000015019	-	0.95	6.23E-04	3.03E-02	5
ENSOARG00000015619	SDHA	0.96	2.20E-06	6.48E-02	16
ENSOARG00000012645	RPL8	0.96	5.27E-05	5.46E-03	9
ENSOARG00000013126	RPS21	0.97	1.33E-03	4.87E-02	13
ENSOARG00000017788	SIRT7	0.99	7.04E-04	3.28E-02	11
ENSOARG00000003683	NDUFA7	0.99	1.89E-05	2.09E-04	5
ENSOARG00000009989	MAP1LC3A	0.99	9.14E-04	3.73E-02	13
ENSOARG00000000129	EEF1D	1.00	8.55E-06	1.64E-03	9
ENSOARG00000008574	RASGRP2	1.00	6.57E-04	3.13E-02	21
ENSOARG00000012384	MAN2B1	1.01	1.56E-04	1.19E-02	5
ENSOARG00000003523	RAB1B	1.04	9.47E-04	3.78E-02	21
ENSOARG00000010800	PRDX2	1.04	3.91E-05	4.43E-03	5
ENSOARG00000015922	ICAM3	1.04	8.25E-04	3.57E-02	5
ENSOARG00000003182	CD81	1.04	3.50E-04	2.07E-02	21
ENSOARG00000010995	-	1.05	8.26E-04	3.57E-02	13
ENSOARG00000003599	-	1.05	7.99E-04	3.49E-02	24
ENSOARG00000009538	ENO1	1.06	2.05E-06	5.94E-04	12
ENSOARG00000000032	ND5	1.06	1.42E-04	1.11E-02	MT
ENSOARG00000002458	RPSA	1.06	9.08E-06	1.67E-03	19
ENSOARG00000012838	DOK1	1.08	6.16E-04	3.01E-02	3
ENSOARG00000011944	SIT1	1.08	1.67E-04	1.25E-02	2
ENSOARG00000005744	MAP4K1	1.09	9.41E-04	3.77E-02	14
ENSOARG00000007866	PSMB9	1.09	1.33E-06	4.37E-04	20
ENSOARG00000008259	LTB	1.09	6.35E-05	6.46E-03	20
ENSOARG00000007749	-	1.09	2.36E-04	1.58E-02	5
ENSOARG00000018702	MCRS1	1.09	1.16E-03	4.40E-02	3
ENSOARG00000013949	SNRNP200	1.10	1.08E-04	9.01E-03	3
ENSOARG00000012851	CD37	1.10	5.02E-05	5.30E-03	14
ENSOARG00000000019	COX2	1.10	6.06E-05	1.13E-03	MT

ENSOARG00000003628	YIF1A	1.11	1.41E-04	1.11E-02	21
ENSOARG00000014733	ETFB; VSIG10L	1.12	9.00E-07	3.11E-04	14
ENSOARG00000011357	FKBP4	1.12	7.35E-04	3.35E-02	3
ENSOARG00000004926	RBM42	1.12	1.32E-04	1.06E-02	14
ENSOARG00000018221	-	1.12	3.70E-04	2.16E-02	11
ENSOARG00000002284	LRRC41	1.13	6.82E-04	3.21E-02	1
ENSOARG00000004207	MYO1F	1.15	4.03E-04	2.29E-02	5
ENSOARG00000025944	-	1.15	7.44E-05	7.42E-03	3
ENSOARG00000007926	PAFAH1B3	1.15	1.36E-03	4.91E-02	14
ENSOARG00000003453	NMRAL1	1.15	8.45E-04	3.61E-02	24
ENSOARG00000002311	SERPINB1	1.15	9.22E-04	3.73E-02	20
ENSOARG00000004143	ALDOA	1.16	8.12E-05	1.33E-03	24
ENSOARG00000007420	YIPF3	1.16	9.24E-04	3.73E-02	20
ENSOARG00000003243	PSMB10	1.17	3.97E-05	4.43E-03	14
ENSOARG00000015485	LOC101108696	1.17	1.39E-05	2.07E-03	20
ENSOARG00000004090	-	1.17	3.00E-04	1.82E-02	19
ENSOARG00000001369	UBE2S	1.18	8.50E-04	3.62E-02	14
ENSOARG00000013443	STUB1	1.19	2.06E-04	1.45E-02	24
ENSOARG00000013165	-	1.19	8.52E-07	3.05E-04	21
ENSOARG00000009339	C43A3; LOC101105C	1.20	1.17E-03	4.43E-02	15
ENSOARG00000019141	PREB	1.20	9.47E-05	8.70E-03	3
ENSOARG00000013373	-	1.21	8.01E-04	3.49E-02	24
ENSOARG00000002381	TMEM131L	1.22	1.59E-05	2.26E-03	17
ENSOARG00000005003	IGFLR1	1.23	1.40E-04	1.11E-02	14
ENSOARG00000015829	PTPN18	1.23	9.60E-05	8.74E-03	2
ENSOARG00000017713	TRAF7	1.25	1.33E-03	4.87E-02	24
ENSOARG00000003866	SIDT2	1.25	4.79E-04	2.55E-02	15
ENSOARG00000001428	RPL28	1.25	3.44E-08	2.55E-05	14
ENSOARG00000012489	AIP	1.25	1.22E-03	4.55E-02	21
ENSOARG00000016897	TRPV2	1.26	6.91E-05	6.96E-03	11
ENSOARG00000005746	SYNGR2	1.27	1.32E-03	4.86E-02	11
ENSOARG00000000033	ND6	1.28	3.85E-05	4.43E-03	MT
ENSOARG00000007553	PLBD2	1.28	8.37E-04	3.60E-02	17

ENSOARG00000013388	LIMD2	1.29	3.05E-05	3.73E-03	11
ENSOARG00000003252	HK3	1.30	1.06E-03	4.10E-02	5
ENSOARG00000014174	CCM2	1.30	3.96E-05	4.43E-03	4
ENSOARG00000005475	KLC4	1.30	5.04E-04	2.60E-02	20
ENSOARG00000000022	ATP6	1.31	4.07E-04	2.30E-02	MT
ENSOARG00000013519	-	1.31	2.77E-06	7.19E-04	3
ENSOARG00000020159	LOC101109545	1.32	2.30E-04	1.56E-02	9
ENSOARG00000017239	MRPL34	1.33	8.42E-04	3.61E-02	5
ENSOARG00000011366	ATP5F1D	1.33	1.84E-04	1.35E-02	5
ENSOARG00000013629	SLC39A3	1.33	8.73E-04	3.67E-02	5
ENSOARG00000013006	MAPK13	1.33	1.62E-04	1.23E-02	20
ENSOARG00000006739	VSIR	1.34	1.29E-04	1.74E-03	25
ENSOARG00000017479	LAMTOR4	1.35	1.28E-05	2.01E-03	24
ENSOARG00000004122	RAB24	1.35	1.21E-05	1.94E-03	5
ENSOARG00000003076	NDUFA3	1.36	6.21E-05	1.15E-03	14
ENSOARG00000013457	PIN1	1.36	4.69E-06	1.04E-03	5
ENSOARG00000016351	PGLS	1.36	3.00E-04	1.82E-02	5
ENSOARG00000007658	PRRC2A	1.37	6.25E-04	3.03E-02	20
ENSOARG00000005975	BAD	1.37	9.94E-05	8.80E-03	21
ENSOARG00000005380	-	1.37	7.02E-04	3.28E-02	5
ENSOARG00000003257	LGALS3BP	1.37	2.50E-04	1.65E-02	11
ENSOARG00000009235	RFXANK	1.37	2.01E-04	1.42E-02	5
ENSOARG00000014412	NDUFS8	1.38	3.16E-05	3.77E-03	21
ENSOARG00000012367	ITGB2	1.38	3.58E-06	9.07E-04	1
ENSOARG00000015048	LOC101113599	1.38	5.36E-08	3.71E-05	9
ENSOARG00000006883	KIF2A	1.38	3.78E-04	2.17E-02	16
ENSOARG00000010851	-	1.39	1.96E-04	1.41E-02	3
ENSOARG00000017984	LOC101121062	1.39	2.85E-04	1.76E-02	25
ENSOARG00000011242	STK11	1.41	1.00E-03	3.98E-02	5
ENSOARG00000009596	LSM10	1.41	1.04E-04	8.87E-03	1
ENSOARG00000018731	ARPC1B	1.41	4.71E-06	1.04E-03	24
ENSOARG00000018014	RRM2B	1.41	2.34E-04	1.57E-02	9
ENSOARG00000005411	NDUFB7	1.42	1.40E-05	2.07E-03	5

ENSOARG00000016156	CDC37	1.43	9.29E-04	3.74E-02	5
ENSOARG00000008352	ARHGEF1	1.44	2.60E-04	1.66E-02	14
ENSOARG00000001909	-	1.44	1.00E-05	1.70E-03	1
ENSOARG00000009445	-	1.45	3.10E-05	3.75E-03	20
ENSOARG00000006493	LONP1	1.45	2.71E-04	1.69E-02	5
ENSOARG00000012585	-	1.46	2.18E-08	1.74E-05	17
ENSOARG00000016621	TSTA3	1.46	5.25E-04	2.70E-02	9
ENSOARG00000010436	MMS19	1.47	4.90E-04	2.57E-02	22
ENSOARG00000001792	CD82	1.47	1.02E-03	4.01E-02	15
ENSOARG00000010422	COPE	1.47	7.73E-08	4.72E-05	5
ENSOARG00000026908	-	1.48	9.16E-04	3.73E-02	14
ENSOARG00000014268	CHMP1A	1.48	2.30E-04	1.56E-02	14
ENSOARG00000014886	SCYL1	1.49	3.42E-05	3.99E-03	21
ENSOARG00000016785	RPS19BP1	1.50	9.44E-06	1.67E-03	3
ENSOARG00000020262	LOC101107687	1.51	4.62E-06	1.04E-03	3
ENSOARG00000009126	-	1.51	1.14E-04	9.49E-03	2
ENSOARG00000019398	RBM23	1.51	1.53E-04	1.17E-02	7
ENSOARG00000008293	RABAC1	1.52	8.99E-05	8.40E-03	14
ENSOARG00000004484	RGS14	1.52	6.97E-07	2.59E-04	5
ENSOARG00000016718	LOC101110095	1.52	8.96E-05	8.40E-03	19
ENSOARG00000007922	BCL7C	1.53	1.26E-03	4.66E-02	24
ENSOARG00000009498	NFKBIE	1.53	4.41E-04	2.43E-02	20
ENSOARG00000020161	DNPEP	1.53	4.02E-06	9.93E-04	2
ENSOARG00000010834	ARHGAP45	1.53	2.69E-04	1.69E-02	5
ENSOARG00000005487	-	1.54	1.20E-03	4.48E-02	24
ENSOARG00000013074	BTBD2	1.54	5.77E-04	2.86E-02	5
ENSOARG00000014009	-	1.54	2.65E-04	1.69E-02	3
ENSOARG00000005857	SIRT2	1.56	5.42E-04	2.77E-02	14
ENSOARG00000013296	NDUFV1	1.56	1.23E-05	1.69E-04	21
ENSOARG00000009440	MAP4K2	1.56	2.18E-04	1.51E-02	21
ENSOARG00000019106	RBCK1	1.58	1.31E-04	1.06E-02	13
ENSOARG00000006624	LOC101113085	1.58	2.48E-04	2.41E-03	11
ENSOARG00000008892	MRPL38	1.58	7.03E-04	3.28E-02	11

ENSOARG00000002015	XAB2	1.58	9.52E-04	3.78E-02	5
ENSOARG00000004725	INTS11	1.58	3.18E-04	1.91E-02	12
ENSOARG00000014413	LOC101117112	1.58	1.35E-06	4.37E-04	18
ENSOARG00000007018	NDUFA11	1.59	6.18E-07	2.46E-04	5
ENSOARG00000000340	ELOB	1.60	8.25E-09	9.52E-06	24
ENSOARG00000009697	GADD45GIP1	1.61	1.84E-04	1.35E-02	5
ENSOARG00000000056	-	1.61	6.62E-04	3.14E-02	12
ENSOARG00000005883	NFKBIB	1.62	5.06E-05	5.30E-03	14
ENSOARG00000002753	RPS9	1.62	1.31E-08	1.23E-05	14
ENSOARG00000007146	LYPLA2	1.63	2.22E-05	2.99E-03	2
ENSOARG00000017094	MZB1	1.64	1.48E-04	1.15E-02	5
ENSOARG00000005024	-	1.64	9.13E-05	8.46E-03	16
ENSOARG00000007688	-	1.65	4.26E-06	1.03E-03	X
ENSOARG00000011786	MRPL28	1.65	1.26E-04	1.03E-02	24
ENSOARG00000002093	DAGLB	1.68	4.58E-06	1.04E-03	24
ENSOARG00000006665	LOC101103862	1.69	6.06E-06	1.31E-03	23
ENSOARG00000005579	RASGRP4	1.69	7.98E-06	1.62E-03	14
ENSOARG00000017508	LOC101102096	1.70	1.56E-07	8.52E-05	20
ENSOARG00000020146	COMMD5	1.70	2.66E-05	3.36E-03	9
ENSOARG00000004138	LOC106991840	1.70	1.73E-08	1.50E-05	21
ENSOARG00000015553	GRINA	1.70	2.42E-05	3.14E-03	9
ENSOARG00000010687	MPG	1.70	9.33E-06	1.67E-03	24
ENSOARG00000008258	RGS19	1.70	5.58E-04	2.78E-02	13
ENSOARG00000014409	-	1.71	8.77E-04	3.67E-02	5
ENSOARG00000011589	NDUFS7	1.72	7.70E-05	7.61E-03	5
ENSOARG00000020673	ITM2C	1.74	4.16E-04	2.32E-02	2
ENSOARG00000005673	DDX39A	1.74	7.93E-04	3.49E-02	5
ENSOARG00000002387	LOC101123116	1.75	3.54E-07	1.60E-04	5
ENSOARG00000012457	-	1.76	1.00E-04	8.80E-03	17
ENSOARG00000020232	MYRFL	1.76	2.23E-04	1.53E-02	3
ENSOARG00000012213	WDR13	1.77	8.86E-04	3.67E-02	X
ENSOARG00000003098	ACD	1.78	4.46E-04	2.43E-02	14
ENSOARG00000005647	TK1	1.79	2.53E-04	1.65E-02	11

ENSOARG00000016386	ATG4D	1.79	8.01E-05	7.84E-03	5
ENSOARG00000009779	CFD	1.79	2.57E-04	1.66E-02	5
ENSOARG00000017798	-	1.80	2.38E-04	1.58E-02	X
ENSOARG00000013159	IL1R2	1.80	4.52E-04	2.45E-02	3
ENSOARG00000003660	TMEM222	1.80	6.31E-04	3.04E-02	2
ENSOARG00000008086	SLC25A39	1.80	2.45E-05	3.14E-03	11
ENSOARG00000000028	ND4	1.81	3.03E-06	7.80E-02	MT
ENSOARG00000016272	GNB2	1.82	2.43E-06	6.47E-04	24
ENSOARG00000015153	LOC101119773	1.82	7.62E-06	1.61E-03	1
ENSOARG00000017643	ASPSCR1	1.84	5.81E-04	2.87E-02	11
ENSOARG00000015049	PPP2R1A	1.86	4.59E-05	4.96E-03	14
ENSOARG00000006571	LOC101103612	1.86	7.89E-06	1.62E-03	23
ENSOARG00000003689	RPS28	1.86	1.02E-05	1.70E-03	5
ENSOARG00000005899	TWF2	1.89	8.33E-05	8.08E-03	19
ENSOARG00000014821	TCF25	1.89	1.17E-05	1.90E-03	14
ENSOARG00000015752	-	1.89	1.57E-05	2.26E-03	21
ENSOARG00000005753	NBEAL2	1.91	8.12E-06	1.62E-03	19
ENSOARG00000003881	SCAP	1.92	7.16E-04	3.30E-02	19
ENSOARG00000010128	ZGPAT	1.92	1.86E-05	2.61E-03	13
ENSOARG00000014024	SPG7	1.95	3.57E-04	2.09E-02	14
ENSOARG00000018872	MRPL41	1.95	9.24E-04	3.73E-02	3
ENSOARG00000013614	FAM173A	1.96	1.39E-03	4.97E-02	24
ENSOARG00000004556	-	1.98	2.41E-09	4.16E-06	26
ENSOARG00000002924	CCND3	1.98	1.95E-07	1.01E-04	20
ENSOARG00000010264	PDLIM2	1.98	3.25E-05	3.83E-03	2
ENSOARG00000012226	BCAT2	1.98	5.47E-04	2.78E-02	14
ENSOARG00000014140	UNC93B1	2.00	1.10E-03	4.22E-02	21
ENSOARG00000012968	GSTP1	2.00	9.58E-09	9.94E-06	21
ENSOARG00000012359	MBD3	2.00	2.87E-05	3.54E-03	5
ENSOARG00000013556	APRT	2.01	5.35E-07	2.22E-04	14
ENSOARG00000004434	PEPD	2.01	3.11E-04	1.88E-02	14
ENSOARG00000012648	FES	2.03	2.00E-05	2.76E-03	18
ENSOARG00000011864	PFKL	2.03	2.27E-04	2.31E-03	1

ENSOARG00000019139	RABGGTA	2.06	7.39E-04	3.35E-02	7
ENSOARG00000004979	-	2.07	4.88E-04	2.57E-02	21
ENSOARG00000016762	-	2.08	8.88E-04	3.67E-02	24
ENSOARG00000009928	TIMM10	2.08	1.35E-05	2.05E-03	15
ENSOARG00000014578	-	2.10	1.19E-03	4.48E-02	14
ENSOARG00000011230	THAP3	2.14	1.93E-06	5.89E-04	12
ENSOARG00000009143	-	2.14	1.02E-04	8.87E-03	18
ENSOARG00000001571	NADSYN1	2.14	7.64E-04	3.43E-02	21
ENSOARG00000008994	-	2.16	1.04E-03	4.10E-02	18
ENSOARG00000000801	DMAP1	2.18	6.40E-04	3.06E-02	1
ENSOARG00000013748	HSPB1	2.19	2.88E-07	1.36E-04	24
ENSOARG00000004520	LOC101112678	2.21	1.97E-04	1.41E-02	18
ENSOARG00000003543	ADAM15	2.21	8.87E-05	8.40E-03	1
ENSOARG00000003126	PBXIP1	2.23	1.06E-03	4.10E-02	1
ENSOARG00000005560	SEMA4A	2.24	7.08E-10	1.47E-06	1
ENSOARG00000010854	-	2.26	3.39E-04	2.02E-02	5
ENSOARG00000026481	-	2.27	1.05E-04	8.89E-03	18
ENSOARG00000000576	GSDMD	2.29	9.63E-06	1.67E-03	9
ENSOARG00000011291	-	2.30	4.23E-10	1.10E-06	X
ENSOARG00000013621	-	2.32	1.63E-04	1.23E-02	2
ENSOARG00000019510	RPS26	2.32	5.97E-08	3.87E-05	3
ENSOARG00000023915	-	2.32	8.98E-04	3.70E-02	7
ENSOARG00000004300	-	2.34	4.59E-04	2.47E-02	5
ENSOARG00000008425	LOC101102454	2.34	1.67E-10	5.78E-07	2
ENSOARG00000006541	-	2.34	1.05E-03	4.10E-02	21
ENSOARG00000007476	SIGIRR	2.35	1.03E-04	8.87E-03	21
ENSOARG00000011018	-	2.36	1.50E-06	4.71E-04	2
ENSOARG00000007634	LOC101105937	2.37	4.74E-05	5.07E-03	13
ENSOARG00000019140	TSPO	2.38	2.22E-05	2.99E-03	3
ENSOARG00000005359	CARHSP1	2.45	1.25E-04	1.03E-02	24
ENSOARG00000008164	GRN	2.46	1.05E-05	1.73E-03	11
ENSOARG00000001516	SERINC2	2.47	2.54E-04	1.65E-02	2
ENSOARG00000002897	DHX30	2.48	8.95E-05	8.40E-03	19

ENSOARG00000003287	DGKZ	2.54	9.83E-05	8.80E-03	15
ENSOARG00000017916	ANAPC11	2.57	2.00E-04	1.42E-02	11
ENSOARG00000013325	LOC101103616	2.63	1.05E-03	4.10E-02	24
ENSOARG00000010831	-	2.68	4.76E-09	7.06E-06	24
ENSOARG00000008507	ALPL	2.73	7.58E-04	3.42E-02	2
ENSOARG00000011715	-	2.77	1.33E-05	2.05E-03	5
ENSOARG00000007908	MMP9	2.80	4.44E-04	2.43E-02	13
ENSOARG00000006669	G6PC3	2.84	2.32E-05	3.06E-03	11
ENSOARG00000006585	ACSS1	2.86	1.47E-07	8.48E-05	13
ENSOARG00000009600	GALK1	2.89	2.51E-04	1.65E-02	11
ENSOARG00000011983	PTPRCAP	2.91	2.57E-13	1.33E-09	21
ENSOARG00000014952	SHARPIN	2.93	7.22E-04	3.32E-02	9
ENSOARG00000001091	U2AF2	3.02	5.03E-04	2.60E-02	14
ENSOARG00000008862	-	3.03	3.54E-15	3.68E-11	18
ENSOARG00000020561	LOC101104728	3.19	5.56E-04	2.78E-02	3
ENSOARG00000009202	-	3.23	1.06E-03	4.11E-02	1
ENSOARG00000006281	ATG16L2	3.25	1.82E-04	1.35E-02	15
ENSOARG00000018894	IL21R	3.57	1.42E-05	2.07E-03	24
ENSOARG00000016461	SPSB3	5.76	9.98E-05	8.80E-03	24
ENSOARG00000011783	MESP2	5.95	4.22E-05	4.66E-03	18
ENSOARG00000011050	-	6.40	2.12E-06	5.94E-04	18

Supplementary table 2. Significantly enriched biological processes obtained using the up-regulated genes from the HSG.

#term ID	term description	observed gene count	background gene count	false discovery rate	matching proteins in your network (labels)
GO:0006091	generation of precursor metabolites and energy	6	21	3.03e-05	COX1,COX2,CYTB,GAPDH,ND4,ND5
GO:0006807	nitrogen compound metabolic process	10	107	3.03e-05	ATP6,COX1,CYTB,EF-1,ENSOARG00000004520,ENSOARG00000019510,GAPDH,ND4,ND5,ZGPAT
GO:0008152	metabolic process	12	158	3.03e-05	ATP6,COX1,COX2,CYTB,EF-1,ENSOARG00000004520,ENSOARG00000019510,GAPDH,ND4,ND5,ND6,ZGPAT
GO:0009167	purine ribonucleoside monophosphate metabolic process	6	14	3.03e-05	ATP6,COX1,CYTB,GAPDH,ND4,ND5
GO:0022900	electron transport chain	5	13	3.03e-05	COX1,COX2,CYTB,ND4,ND5
GO:0034641	cellular nitrogen compound metabolic process	9	67	3.03e-05	ATP6,COX1,CYTB,EF-1,ENSOARG00000019510,GAPDH,ND4,ND5,ZGPAT
GO:0046034	ATP metabolic process	6	14	3.03e-05	ATP6,COX1,CYTB,GAPDH,ND4,ND5
GO:1901564	organonitrogen compound metabolic process	9	75	3.03e-05	ATP6,COX1,CYTB,EF-1,ENSOARG00000004520,ENSOARG00000019510,GAPDH,ND4,ND5

GO:0055114	oxidation-reduction process	7	45	3.39e-05	COX1,COX2,CYTB,GAPDH,ND4,ND5,ND6
GO:0006119	oxidative phosphorylation	4	6	4.23e-05	COX1,CYTB,ND4,ND5
GO:0044237	cellular metabolic process	10	122	5.59e-05	ATP6,COX1,COX2,CYTB,EF-1,ENSOARG00000019510,GAPDH,ND4,ND5,ZGPAT
GO:0044238	primary metabolic process	10	126	7.04e-05	ATP6,COX1,CYTB,EF-1,ENSOARG00000004520,ENSOARG00000019510,GAPDH,ND4,ND5,ZGPAT
GO:0045333	cellular respiration	4	8	8.83e-05	COX1,CYTB,ND4,ND5
GO:0006139	nucleobase-containing compound metabolic process	7	56	0.00010	ATP6,COX1,CYTB,GAPDH,ND4,ND5,ZGPAT
GO:0071704	organic substance metabolic process	10	133	0.00010	ATP6,COX1,CYTB,EF-1,ENSOARG00000004520,ENSOARG00000019510,GAPDH,ND4,ND5,ZGPAT
GO:0016310	phosphorylation	5	24	0.00018	COX1,CYTB,GAPDH,ND4,ND5
GO:0042773	ATP synthesis coupled electron transport	3	5	0.00062	CYTB,ND4,ND5
GO:0044271	cellular nitrogen compound biosynthetic process	5	50	0.0035	ATP6,EF-1,ENSOARG00000019510,GAPDH,

GO:1902600	proton transmembrane transport	3	11	0.0035	ATP6,COX1,COX2
GO:1901566	organonitrogen compound biosynthetic process	4	28	0.0036	ATP6,EF-1,ENSOARG00000019510,GAPDH
GO:0042775	mitochondrial ATP synthesis coupled electron transport	2	3	0.0079	CYTB,ND4

Supplementary table 3. significant enriched molecular function obtained using the up-regulated genes from HSG group.

#term ID	term description	observed gene	background gene count	false discovery rate	matching proteins in your network (labels)
GO:0016491	oxidoreductase activity	7	40	0.00012	COX1,COX2,CYTB,GAPDH,ND4,ND5,ND6
GO:0003824	catalytic activity	9	105	0.00045	ATP6,COX1,COX2,CYTB,ENSOARG00000004520,GAPDH,ND4,ND5,ND6
GO:0008137	NADH dehydrogenase (ubiquinone) activity	3	7	0.0048	ND4,ND5,ND6
GO:0009055	electron transfer activity	3	8	0.0048	COX1,COX2,CYTB
GO:0015078	proton transmembrane transporter activity	3	10	0.0048	ATP6,COX1,COX2

Supplementary table 4. List of all modules associated with lactose. The module name with number of genes, correlation value and p-value are reported in relation with the lactose trait.

Module_name	Number of genes	Correlation_value	p-value
MEblack	614	0.05779468	0.372195198
MEblue	2467	0.47512863	0.0346639879
MEbrown	2049	0.4325791	0.381041686
MEbrown4	34	0.06081007	0.435999987
MEcyan	389	-0.38869439	0.53115172
MEdarkgreen	131	-0.32633967	0.008812693
MEdarkgrey	104	-0.20514673	0.946787661
MEdarkmagenta	65	0.06138097	0.603068598
MEdarkolivegreen	70	0.46840569	0.777020088
MEdarkorange	96	0.19026107	0.055938911
MEdarkorange2	37	-0.43538628	0.343095979
MEdarkred	134	-0.22697536	0.474780165
MEdarkturquoise	126	0.17565644	0.33856232
MEfloralwhite	43	-0.20009291	0.421503152
MEgreen	1042	-0.57253985	0.911391936
MEgreenyellow	424	0.56293533	0.113089613
MEgrey	9951	0.43327898	0.531020836
MEgrey60	204	0.2450209	0.94895646
MEivory	45	-0.27512806	0.704903591
MElightcyan	226	-0.82406466	0.602132664
MElightcyan1	48	-0.03406998	0.902088755
MElightgreen	177	0.64292232	0.0310130226
MElightsteelblue1	48	-0.13220617	0.651476866
MElightyellow	147	0.32512839	0.002904869
MEmagenta	486	-0.3346861	0.275681334
MEmediumpurple3	53	-0.18193795	0.419170684
MEmidnightblue	346	-0.06129696	0.257770825
MEorange	100	0.1830075	0.868841879
MEorangered4	51	0.08489356	0.769739584

MEpaleturquoise	74	0.15369878	0.261181532
MEpink	488	0.20317819	0.632934016
MEplum1	52	0.10324017	0.220691453
MEpurple	425	-0.55989029	0.900534865
MEred	707	-0.15124481	0.639044449
MEroyalblue	145	0.42241428	0.082944416
MEsaddlebrown	80	-0.27004566	0.146303408
MEsalmon	410	-0.05347002	0.040217918
MEsienna3	63	0.27300849	0.485703373
MEskyblue	89	-0.10676245	0.96624799
MEskyblue3	59	-0.25855254	0.331906992
MEsteelblue	77	-0.10336313	0.036319551
MEtan	418	0.12578527	0.030940193
MEturquoise	3811	0.07309515	0.174808667
MEviolet	73	-0.08857551	0.56345995
MEwhite	94	-0.18046995	0.983742855
MEyellow	1377	0.27661037	0.122735611
MEyellowgreen	61	0.49253135	0.988108202

Supplementary table 5. List of all genes included in the lightgreen module. Gene Ids, gene symbols, gene correlation value to the lactose, correlation significance, module membership value and significance are reported.

GENE ID	Gene Symbol	GS.lactose	p.GS.lactose	MM.lightgreen	p.MM.lightgreen
101104790	LOC101104790	0.647147997	0.043111693	0.961327562	9.34E-06
101116308	TIMM29	0.543048101	0.104767381	0.952453152	2.11E-05
101119137	MRPS18C	0.63713571	0.047556772	0.946696538	3.31E-05
101107089	ZNF786	0.553043155	0.097279148	0.945786056	3.54E-05
101115173	RPS15A	0.629395992	0.05119522	0.940489963	5.10E-05
101118432	RHNO1	0.489440998	0.151063914	0.931464728	8.88E-05
106991510	-	0.600179776	0.066584961	0.929610212	9.86E-05
101123618	CSRP2	0.60222723	0.065418805	0.926852073	0.00011459
105616076	LOC105616076	0.469504595	0.17098243	0.925546735	0.000122793
101120908	SEMA4F	0.521446897	0.122165496	0.924739376	0.000128079
101116510	NDUFC1	0.490022958	0.150504722	0.922050262	0.0001469
101117690	LOC101117690	0.44005445	0.203130035	0.920232627	0.000160727
105615810	-	0.578836177	0.079556864	0.920084566	0.000161894
101107905	C19H3orf67	0.578836177	0.079556864	0.920084566	0.000161894
105612398	LOC105612398	0.578836177	0.079556864	0.920084566	0.000161894
101115477	HYDIN	0.578836177	0.079556864	0.920084566	0.000161894
101109300	LOC101109300	0.578836177	0.079556864	0.920084566	0.000161894
101105201	FFAR4	0.578836177	0.079556864	0.920084566	0.000161894
101108617	HGD	0.578836177	0.079556864	0.920084566	0.000161894
101113473	FCN3	0.578836177	0.079556864	0.920084566	0.000161894
101111709	STEAP1	0.578836177	0.079556864	0.920084566	0.000161894
105611299	LOC105611299	0.578836177	0.079556864	0.920084566	0.000161894
105602059	-	0.578836177	0.079556864	0.920084566	0.000161894
105606371	DPEP2NB	0.578836177	0.079556864	0.920084566	0.000161894
106991765	LOC106991765	0.578836177	0.079556864	0.920084566	0.000161894
101115527	LOC101115527	0.578836177	0.079556864	0.920084566	0.000161894
106990385	LOC106990385	0.578836177	0.079556864	0.920084566	0.000161894
101116655	GPR137C	0.605642896	0.06350329	0.914773271	0.000208045
101109153	TESK1	0.715995901	0.019863795	0.911743493	0.000238348
101105144	SNRPG	0.595785924	0.069133244	0.911559026	0.000240292

101116886	LOC101116886	0.657202712	0.03893719	0.911463683	0.000241301
100307042	FEN1	0.641903796	0.045403525	0.910124408	0.000255811
101102229	LSM8	0.591141015	0.07189544	0.908504286	0.000274209
101115546	RWDD2A	0.505385902	0.136196368	0.903674758	0.000334837
678684	NARS1	0.722295081	0.018312682	0.901836054	0.000360319
101110479	ERLIN2	0.345858104	0.327623849	0.899523709	0.000394351
100037674	TYMS	0.680817266	0.030223647	0.899346783	0.000397048
101118201	MRPL10	0.653850179	0.040297386	0.897898735	0.000419637
101112137	WDR18	0.576644053	0.080974811	0.895128395	0.000465458
101109824	THAP11	0.676007697	0.031878118	0.89511197	0.00046574
101118221	SRSF9	0.532535252	0.113025571	0.894903891	0.000469325
105610222	LOC105610222	0.734653006	0.015517303	0.892596686	0.000510443
101109279	ANGEL1	0.439737099	0.203494191	0.890537512	0.000549313
101103759	KDM2B	0.383517918	0.273943058	0.887761501	0.000605088
101117774	NCOR1	0.449037112	0.192979435	0.886954579	0.000622049
443301	YWHAH	0.450813683	0.191007773	0.885882729	0.000645112
101109145	TYSND1	0.628691013	0.051535543	0.881964874	0.000734756
101119635	ANKRD54	0.665228648	0.03580738	0.881056226	0.00075678
101105702	B4GAT1	0.484191095	0.156165335	0.879699206	0.000790565
101104663	SLBP	0.486281486	0.154121781	0.879641339	0.000792029
101123533	LOC101123533	0.655063463	0.039801497	0.87757706	0.000845585
101115056	LOC101115056	0.54855869	0.100595386	0.876663684	0.000870105
105606241	-	0.453596536	0.187943157	0.874995538	0.000916219
106990119	PIGZ	0.453596536	0.187943157	0.874995538	0.000916219
105604743	LOC105604743	0.453596536	0.187943157	0.874995538	0.000916219
101116544	NCMAP	0.52471053	0.119429236	0.873167604	0.000968768
105606686	LOC105606686	0.566555448	0.087710308	0.870590051	0.001046551
101119924	TCFL5	0.775313018	0.008416391	0.870328173	0.0010547
101117939	ENY2	0.65604912	0.03940168	0.869698199	0.001074493
101116505	CCDC34	0.434294518	0.209798333	0.86742894	0.001148034
101110182	LCMT2	0.511539062	0.130709531	0.865400321	0.001216815
101113437	NDUFB1	0.380780974	0.277671514	0.864104699	0.001262281
101111410	ATP5MD	0.530017905	0.115061688	0.863230874	0.001293634

101121403	LOC101121403	0.443014557	0.199751569	0.860004687	0.001414303
101103639	LOC101103639	0.613683488	0.059140394	0.858534458	0.001471918
101104903	GALNTL5	0.436390512	0.207357377	0.858054432	0.001491092
101109616	COQ2	0.334327777	0.345067402	0.857809882	0.001500929
101107429	WBP11	0.237269224	0.509219293	0.85770601	0.001505122
100499499	WSB2	0.50282284	0.138522917	0.857000487	0.001533825
100270716	SLIRP	0.583282439	0.076730367	0.85529442	0.001604872
101103082	LOC101103082	0.723020614	0.018139601	0.85436804	0.001644434
101122056	LOC101122056	0.309592112	0.384030436	0.849950714	0.00184286
101106788	ZC3H18	0.610762271	0.060701871	0.849797026	0.001850061
101111239	LAMTOR5	0.712123882	0.020861034	0.84805534	0.001933095
105607690	LOC105607690	0.390805204	0.264149714	0.845342292	0.00206776
100037664	LOC100037664	0.448475917	0.193604721	0.845246089	0.002072656
101123176	RAB11FIP3	0.429067589	0.215957324	0.842135957	0.002235502
101104080	IDH3B	0.774208048	0.008570793	0.840410098	0.002329749
101108712	UNKL	0.671671839	0.033421475	0.835905067	0.002589247
101122275	TIGD6	0.499725571	0.141366677	0.835171871	0.002633369
105604928	LOC105604928	0.612802513	0.05960848	0.833877691	0.002712567
101114533	-	0.638575683	0.046899455	0.831681914	0.002850843
101108082	ZSCAN2	0.568023131	0.086708877	0.829300132	0.003006492
101120180	LOC101120180	0.715465598	0.019998384	0.827213196	0.003147818
105604655	LOC105604655	0.389235049	0.266243346	0.827099909	0.003155623
100144758	CCNA2	0.44058628	0.202520615	0.826068574	0.003227326
105612497	LOC105612497	0.881068056	0.00075649	0.823176305	0.00343465
101109225	PGM1	0.417458297	0.230002931	0.813962158	0.00415896
101119090	LOC101119090	0.57181715	0.084154236	0.813084049	0.004233252
101108844	MYH10	0.545602454	0.10282018	0.810051317	0.004497147
101109243	LOC101109243	0.758474923	0.01099203	0.809090575	0.004583144
101121755	C2H2orf72	0.48144763	0.15887203	0.808977005	0.004593387
105612514	-	0.434039293	0.210096689	0.808323349	0.004652659
101121360	LOC101121360	0.5393743	0.107608503	0.804437884	0.005016319
101111112	NUCKS1	0.289992067	0.416351089	0.802889041	0.005166782
105612165	-	0.382089912	0.275884969	0.800247602	0.005430771

105611576	LOC105611576	0.655184393	0.039752297	0.798664158	0.005593554
101119859	PRMT6	0.161510384	0.655768833	0.796588915	0.005812121
101110003	SUMF1	0.378039033	0.28143422	0.793550716	0.006142979
443093	GOT2	0.472806102	0.167581322	0.786278542	0.006989003
101110984	TAF5	0.301091995	0.39789333	0.77767541	0.008092898
105613179	LOC105613179	0.242223316	0.5001456	0.776960267	0.00818988
101119038	SEM1	0.709682938	0.021507157	0.775004436	0.008459311
101113939	BCKDHB	0.672931723	0.032967904	0.774559678	0.008521443
106990861	-	0.484389153	0.155971018	0.771812561	0.008912381
105610712	LOC105610712	0.524661704	0.119469888	0.771134253	0.009010825
106990880	-	0.644079903	0.044442863	0.76881376	0.009353408
101114633	ELMO2	0.539574621	0.10745235	0.768783179	0.009357984
443297	LOC443297	0.345139367	0.328697589	0.768316438	0.009428008
106991442	LOC106991442	0.119576304	0.74213496	0.767712537	0.009519157
101107013	LOC101107013	0.506433348	0.135252533	0.765291005	0.009890897
101105990	SF3B6	0.258637494	0.470581899	0.761772371	0.010449117
101106662	LOC101106662	0.320415347	0.366726584	0.754123466	0.011738427
101117404	BUB1	0.709865895	0.021458256	0.75110253	0.012277054
106991640	LOC106991640	0.821384725	0.00356777	0.744193963	0.013573531
101108353	NDUFB4	0.611856464	0.060113852	0.740583054	0.014287876
101123048	ENPP1	0.265102843	0.45915367	0.736051705	0.015220928
101111976	SCRIB	0.713260195	0.020564873	0.726993735	0.017211855
101110721	ADGRG7	0.364833428	0.299938549	0.721850656	0.018419267
101113315	TPRKB	0.230008936	0.522639576	0.721264486	0.018560502
101123463	NDUFA5	0.11161551	0.75886014	0.714268257	0.020304581
105602906	-	0.419797516	0.227132238	0.709792669	0.021477819
101114362	PIGH	0.328542537	0.353993427	0.709776564	0.021482123
101121251	LOC101121251	0.747472143	0.01294698	0.698393899	0.024675481
101105752	CENPE	0.438806055	0.204564735	0.695303655	0.025595621
101109979	GEM	0.639444487	0.046505814	0.690535834	0.027060985
101115508	LOC101115508	0.369014122	0.294012073	0.690305988	0.027133044
101109126	C19H3orf20	0.219790133	0.541769538	0.68332797	0.029383675
101104998	RASL11B	0.467026425	0.17356218	0.679053986	0.030823246

105610271	-	0.555773132	0.095294813	0.677580815	0.031330362
101102475	TSPAN12	0.555773132	0.095294813	0.677580815	0.031330362
101104641	HOXD13	0.555773132	0.095294813	0.677580815	0.031330362
101105689	-	0.384875565	0.272103751	0.675370988	0.032101657
106990454	LOC106990454	0.538233266	0.108500686	0.671174458	0.033601697
101106995	LSS	0.384092511	0.273163789	0.668275718	0.034665161
106991532	-	0.515189404	0.127520014	0.651183361	0.041401931
101114007	NDUFA6	0.819242158	0.003731783	0.648401664	0.042575544
105606223	ATP5F1E	0.218393993	0.544404569	0.643828378	0.044553199
100302554	SLC39A6	0.03721404	0.918706931	0.634629802	0.048715271
101120255	PIGS	0.534542161	0.111418603	0.633018301	0.049470128
101114418	ESCO2	0.355176945	0.313867339	0.615130496	0.058376828
105603374	LOC105603374	0.714022167	0.020367913	0.608366961	0.062002292
101112287	LOC101112287	0.614817747	0.058541308	0.592058733	0.071344104
101121287	GAN	0.134628063	0.710781024	0.579058433	0.079414003
101121549	ANKRD13A	0.222479259	0.536708537	0.506034879	0.135611111
101105823	LOC101105823	0.264638258	0.459970711	0.449635088	0.19231447
101108653	-	-0.358103504	0.309610761	-0.354376374	0.315037058
101123347	HOOK3	-0.498390278	0.1426036	-0.420492523	0.226283266
101117110	KIAA1671	-0.347623561	0.324994102	-0.52248557	0.121290477
100216462	FN1	-0.186152035	0.606612252	-0.551460794	0.098441243
101117907	CACNB4	-0.887354269	0.000613605	-0.579941154	0.078848248
101116239	LIG4	-0.75177354	0.012155948	-0.610410682	0.060891615
101111560	LOC101111560	-0.16960484	0.639478891	-0.627883565	0.051927175
105609364	LOC105609364	-0.617500298	0.057140417	-0.634982632	0.048551029
106991526	LOC106991526	-0.476460028	0.163864701	-0.635505404	0.04830836
101116782	WNT2B	-0.519388325	0.123911289	-0.638466622	0.046949026
101104867	DLG2	-0.080329695	0.825408314	-0.646225049	0.043509286
101105604	SNUPN	-0.796015073	0.005873616	-0.664931398	0.035920152
101105678	SIRPB2	-0.26041491	0.46742771	-0.6672923	0.035031065
101121120	CLIP1	-0.864078173	0.001263224	-0.671746973	0.033394308
101109836	TAF8	-0.78760984	0.006828295	-0.695009765	0.025684335
101117981	KCNJ15	-0.678595806	0.030980364	-0.700138693	0.024166121

106991416	-	-0.227996798	0.526384323	-0.700424793	0.024083295
101120670	MAX	-0.50014657	0.140978058	-0.706895249	0.022261797
101111434	LOC101111434	-0.510752433	0.131403237	-0.755682671	0.011467017
105604793	PILRB	-0.479488561	0.160822017	-0.759567941	0.010809924
101104522	LOC101104522	-0.585430152	0.075388692	-0.764418702	0.010027277
101118600	LOC101118600	-0.31343303	0.377844755	-0.774243132	0.00856586
105603230	LOC105603230	-0.591051554	0.071949333	-0.780437331	0.007725967
101122115	KIAA0408	-0.616978123	0.057411355	-0.803779781	0.005079863
101105233	FGD4	-0.886924433	0.000622689	-0.80559691	0.00490579
101102594	GMPR	-0.49669817	0.144180517	-0.831187561	0.002882661
101120236	LOC101120236	-0.590989215	0.071986904	-0.831267276	0.002877513
101116103	WLS	-0.396992102	0.255988608	-0.846420631	0.00201345
101110450	NAIP	-0.577426855	0.080466609	-0.847847454	0.001943182
101102278	-	-0.708747112	0.021758491	-0.853739208	0.001671688
101120892	HMGB2	-0.657742429	0.038721147	-0.871454765	0.001019968

Supplementary table 6. List of all genes included in the blue module. Gene Ids, gene symbols, gene correlation value to the lactose, correlation significance, module membership value and significance are reported.

GENE ID	Gene symbol	GS.lactose	p.GS.lactose	MM.blue	p.MM.blue
101101898	PHF20L1	-0.925753171	0.000121468	-0.601883304	0.065613751
101116364	SENP2	-0.906297453	0.000300811	-0.531829753	0.113593907
101114952	TNFAIP8	-0.880051619	0.000781687	-0.633514846	0.049236712
101107913	TIGD3	0.851148358	0.001787439	0.675131294	0.032186086
101105634	CLK1	-0.8425392	0.002213884	-0.714898423	0.020143027
101122040	GIT1	0.489642073	0.150870564	0.998908222	0.00000000000621
101111864	SH3BGRL3	0.462260309	0.178588362	0.998836612	0.000000000008
101115757	AGTPBP1	-0.834371979	0.002682119	-0.506361816	0.135316861
101122880	PLLP	0.828511923	0.003059321	0.563876851	0.089557021
106991357	-	0.815039178	0.004069119	0.454584358	0.186862313
106992023	-	0.810929231	0.004419579	0.610513768	0.060835942
106991584	LOC106991584	0.80068561	0.005386346	0.500518577	0.140635208
101109051	PTBP2	-0.800030491	0.005452889	-0.576231536	0.081243451
101107003	LOC101107003	0.484347222	0.156012145	0.998635073	0.0000000000152
101102615	ACAP2	-0.791057335	0.006424342	-0.60595925	0.063327763
101121537	HERPUD1	-0.787986305	0.00678333	-0.476719201	0.163602977
101122014	LOC101122014	0.784520495	0.007205316	0.447581825	0.194603366
101121145	CXCL12	0.776969152	0.00818867	0.661637964	0.037185733
101114872	CHAC1	0.774729187	0.008497725	0.639263149	0.046587793
101104233	CLK4	-0.774100065	0.008585989	-0.594688556	0.069779461
101102536	OSBPL6	0.773628686	0.008652545	0.611026073	0.060559759
101112423	GGT5	0.484338292	0.156020904	0.998063593	0.0000000000614
101107640	PTPRG	0.770457623	0.009109788	0.695182045	0.025632305
106991010	-	0.493132706	0.147537946	0.998056283	0.0000000000623
101120464	KIAA0556	-0.769729647	0.009217114	-0.447129332	0.195109916
101106288	LOC101106288	0.497254948	0.143660473	0.997947474	0.0000000000775
105605852	DGCR6L	0.493788813	0.146916586	0.997638075	0.000000000136
105611599	-	0.764149093	0.010069695	0.586955571	0.074445067
100135455	TPT1	0.475754505	0.164578441	0.997394817	0.000000000201

101122631	MRPS11	0.506053542	0.135594303	0.997349134	0.000000000215
101106848	RPLP1	0.521182648	0.122388735	0.997332671	0.000000000221
101103086	TOP1	-0.760389972	0.010674371	-0.724169078	0.01786795
101120124	GBE1	-0.757374985	0.011177455	-0.75456196	0.011661648
101115969	LOC101115969	-0.754115283	0.011739864	-0.781918489	0.007534123
105603777	LOC105603777	0.751666177	0.012175269	0.604998626	0.063861745
101114953	SPATA24	0.748679377	0.012721436	0.68309645	0.029460457
101119684	ST14	0.482343455	0.157985127	0.997311345	0.000000000228
106991032	-	0.748056084	0.012837537	0.653152331	0.040584482
101110516	WTAP	-0.747816888	0.012882289	-0.745049654	0.013407985
105608579	LOC105608579	0.743276628	0.013752586	0.629717691	0.05104042
101119685	PTPRCAP	0.472268223	0.168132647	0.99715832	0.000000000284
101123646	AFMID	0.738598571	0.014691437	0.504316712	0.137163956
101106887	ARPP21	-0.737344204	0.014950585	-0.62033134	0.055686191
443519	HIF1A	-0.735806657	0.015272565	-0.606032594	0.063287115
101109127	UBR2	-0.735379466	0.015362874	-0.658370668	0.03847069
105602038	LOC105602038	0.734569403	0.015535144	0.680785858	0.030234257
101123096	PLEKHA3	-0.734196846	0.015614823	-0.90273219	0.000347728
101106827	HTR2B	0.732368009	0.016010088	0.680239589	0.030419202
101118832	OSBPL5	0.732368009	0.016010088	0.680239589	0.030419202
100101234	CHID1	0.495349192	0.14544525	0.997089502	0.000000000313
443277	CEBPB	0.490971985	0.149595523	0.997088428	0.000000000313
101108073	ADM5	0.454609218	0.186835159	0.996979292	0.000000000363
101102511	PLEKHB1	0.725399982	0.017579947	0.642461254	0.04515612
101103437	CCDC85B	0.501826481	0.139433866	0.996706318	0.000000000513
101105152	DLD	-0.724703359	0.017742542	-0.864853922	0.001235841
105602975	LOC105602975	0.71992277	0.018886604	0.662803803	0.036734343
101123565	-	-0.715523272	0.019983716	-0.705050915	0.022770972
101118775	CLEC1A	-0.713613076	0.020473495	-0.376661161	0.283335394
105604154	POLR2L	0.497389958	0.143534543	0.996605394	0.000000000579
101103196	RPLP2	0.479067604	0.161242892	0.996575654	0.000000000599
101108904	C3H12orf57	0.451160777	0.190623949	0.996325972	0.000000000794
105616104	LOC105616104	0.710222113	0.021363265	0.472368309	0.168029976

101122265	NCKAP5L	0.709009378	0.021687851	0.580646805	0.078397859
101111579	TM6SF1	-0.708702388	0.021770552	-0.625619864	0.05303565
101111447	LRRK2	-0.708196515	0.021907306	-0.869204052	0.001090206
106991575	-	0.70661166	0.022339573	0.690156477	0.027179987
101104014	BAZ1A	-0.705776334	0.022569756	-0.708024418	0.021953963
101116783	FADD	0.42386885	0.222184691	0.996095156	0.00000000101
101113141	LOC101113141	0.460780803	0.180165925	0.995746518	0.00000000142
101116108	DPM3	0.455443844	0.185924871	0.995573493	0.00000000167
101122675	-	0.476400059	0.163925297	0.995472816	0.00000000183
105608232	-	-0.701254141	0.023844301	-0.368468896	0.2947814
101114714	NATD1	0.46019703	0.18079065	0.995396235	0.00000000195
105604767	LOC105604767	0.697519285	0.024933561	0.676732527	0.031624932
101103448	ATXN7L2	0.697519285	0.024933561	0.676732527	0.031624932
105604388	LOC105604388	0.697160757	0.025039887	0.683210362	0.029422662
101119166	FAM177A1	-0.697020886	0.025081452	-0.727234934	0.017156618
101117149	MTLN	0.475528399	0.164807575	0.995382046	0.00000000198
101112463	LOC101112463	0.47365942	0.166708894	0.995372206	0.000000002
101117497	PTPN12	-0.691815606	0.026662161	-0.670933243	0.033689337
105604618	LOC105604618	0.687682904	0.027964718	0.60702349	0.062739617
101111201	PARP10	0.466472214	0.174142256	0.995319821	0.00000000209
101108092	LOC101108092	0.684716945	0.028925897	0.539431205	0.107564131
101112935	NDUFS8	0.458085943	0.183060501	0.995249225	0.00000000222
105610551	LOC105610551	-0.682831519	0.029548487	-0.422630168	0.223683391
101113745	LOC101113745	0.682601718	0.02962499	0.672110541	0.033263061
101122188	MXD3	0.479955405	0.160356037	0.995206559	0.0000000023
101110890	PITPNM1	0.520270171	0.123161546	0.995137322	0.00000000243
101119562	HERC5	-0.677874387	0.031228855	-0.626398893	0.052652425
106991482	LOC106991482	-0.676237383	0.031797739	-0.479528197	0.160782423
101113696	LOC101113696	0.509161779	0.132812915	0.995045044	0.00000000262
101105879	C2H9orf43	-0.672926706	0.032969702	-0.718378311	0.019266871
105605853	-	-0.672416112	0.033153021	-0.356121292	0.312490482
101110586	CCDC124	0.454765228	0.186664807	0.995044392	0.00000000262
100125354	PLIN2	0.670424184	0.033874798	0.519361001	0.123934566

780466	MIF	0.440461801	0.202663159	0.994637437	0.00000000359
101123116	LOC101123116	0.443904349	0.198742453	0.994540611	0.00000000386
443470	IGFBP4	0.458793664	0.182297692	0.994475934	0.00000000405
101111148	ANTKMT	0.458962011	0.182116518	0.99439275	0.0000000043
101116953	AMDHD2	0.458774638	0.182318174	0.994282268	0.00000000464
101114741	FBXL15	0.432168839	0.212290698	0.994270273	0.00000000468
101113725	CTSL	0.662248051	0.036949055	0.681154081	0.030110025
101103529	ZNF500	0.49709445	0.143810264	0.994266577	0.0000000047
101109691	-	0.49709445	0.143810264	0.994266577	0.0000000047
101109464	RECQL5	0.49709445	0.143810264	0.994266577	0.0000000047
101114547	AKTIP	-0.656836892	0.039084085	-0.6991754	0.024446435
101103833	KLHL36	0.474644158	0.165705489	0.9941297	0.00000000516
101114160	INTS1	0.44002382	0.203165166	0.9940695	0.00000000537
101121636	PDCD1	0.484061924	0.156292146	0.993896343	0.00000000603
101119766	TREX1	0.425335656	0.220417408	0.993807078	0.00000000639
101104198	CAPN10	0.497758983	0.143190682	0.99328053	0.00000000885
101114106	TPGS1	0.444528394	0.198036496	0.992876045	0.0000000112
106991813	-	0.653745208	0.040340484	0.507984861	0.133861902
101103141	LOC101103141	0.651898195	0.041103889	0.490828715	0.149732566
105613233	MS4A3	0.651645991	0.041208877	0.631520481	0.050178682
101109189	LOC101109189	0.651645991	0.041208877	0.631520481	0.050178682
101117024	RIPOR2	-0.650168764	0.041827443	-0.697395599	0.024970206
101108465	MRPL41	0.497388661	0.143535753	0.992825404	0.0000000115
101109634	PPDPF	0.473566041	0.166804232	0.992738953	0.0000000121
101105220	EIF4G3	-0.649495098	0.042111589	-0.755544533	0.011490883
101122114	TAB2	-0.649099346	0.042279117	-0.836220723	0.002570416
101109097	CMYA5	-0.648148236	0.042683563	-0.509003888	0.132953348
101118496	ELOB	0.451935069	0.189769349	0.992709781	0.0000000122
780444	BAD	0.484291798	0.156066516	0.992667787	0.0000000125
443446	THRA	0.391381213	0.263383944	0.992632694	0.0000000128
101112572	LOC101112572	0.643298709	0.044786147	0.675711418	0.031982005
101119561	PLA2G12A	-0.642183952	0.045279076	-0.616119511	0.057858699
100147789	SLC25A6	0.478961436	0.161349143	0.99262883	0.0000000128

101110843	RELL1	-0.638995515	0.046708959	-0.622371463	0.054653558
101117061	GTPBP6	0.460746512	0.180202587	0.992622318	0.0000000128
101121367	TMED6	-0.636513692	0.047842602	-0.678418788	0.031041213
101117771	SLC52A2	0.526777988	0.117715799	0.992538669	0.0000000134
101113198	FBP1	0.635807567	0.048168466	0.647767978	0.042845986
101105211	ORAI3	0.633840872	0.049083854	0.645226524	0.043942197
101120878	-	0.394751272	0.258928157	0.992527465	0.0000000135
100192427	LOC100192427	0.419869297	0.227044472	0.992431552	0.0000000142
101117506	ABHD17A	0.46035892	0.180617275	0.992378433	0.0000000146
101103941	GPR35	0.465982507	0.174655776	0.99228435	0.0000000154
101103106	DEAF1	0.430488353	0.21427307	0.992169106	0.0000000163
106991840	LOC106991840	0.448143774	0.193975353	0.992147589	0.0000000165
101103316	RPS28	0.461470371	0.179429633	0.992057056	0.0000000172
101121482	ZNHIT1	0.480594865	0.159719081	0.992034398	0.0000000174
101111663	MTFP1	0.50600996	0.135633555	0.992003761	0.0000000177
101104227	TMEM184B	0.500936881	0.140250299	0.991971544	0.000000018
101105716	HSD3B7	0.533282133	0.112425844	0.991964575	0.0000000181
101104173	LIG1	0.534512017	0.111442634	0.991961688	0.0000000181
101123464	PDLIM7	0.434722727	0.209298309	0.991890767	0.0000000187
101116037	DCTN1	0.451163084	0.190621399	0.99178896	0.0000000197
101112372	LFNG	0.403638887	0.247378581	0.991787172	0.0000000197
101117023	MRPL14	0.492023843	0.148591713	0.991776839	0.0000000198
101123626	ZYX	0.43234731	0.212080788	0.991725932	0.0000000203
101114949	TRABD	0.422686548	0.223615052	0.991725328	0.0000000203
101112896	ALG12	0.483869305	0.156481361	0.991703496	0.0000000205
101110018	BTBD19	0.620788067	0.055453901	0.632044082	0.049930226
101102439	AP5B1	0.458805734	0.182284699	0.991654267	0.000000021
101112238	SPNS2	0.414420794	0.233761065	0.991637271	0.0000000212
101102122	LOC101102122	0.551912218	0.09810882	0.991313429	0.0000000247
101115257	-	-0.61823223	0.056762068	-0.823849324	0.003385577
101119858	FANCF	0.618195561	0.056780983	0.631686511	0.05009981
101101844	LRRFIP1	-0.61731982	0.057233965	-0.660637005	0.037576261
106991908	-	0.616507951	0.057656035	0.60299862	0.064982943

100302318	GATD3A	0.493481963	0.147206987	0.991283706	0.000000025
101105367	LOC101105367	0.421383105	0.225198034	0.991128201	0.0000000268
101120024	CLEC3B	0.454586934	0.186859499	0.991028939	0.000000028
101104621	FAM53B	0.481365997	0.158952998	0.990943085	0.0000000291
105601982	LOC105601982	0.61255821	0.059738716	0.672492609	0.033125512
101120662	ZFYVE16	-0.611443226	0.060335483	-0.833738286	0.002721199
101108347	RPP21	0.496504645	0.144361542	0.990889098	0.0000000298
101104417	RBM38	0.486039004	0.154357996	0.990690817	0.0000000325
101119657	GALK1	0.452172533	0.189507706	0.990687727	0.0000000325
101123166	PNPLA2	0.43857654	0.204829138	0.990628966	0.0000000334
105603818	LOC105603818	0.608059433	0.062170564	0.58606851	0.074992865
101119259	CISH	0.405506654	0.244988628	0.990551612	0.0000000345
101121826	KAT6A	-0.606930515	0.062790855	-0.829277224	0.003008018
101115712	CCNB2	0.606447772	0.063057337	0.62484874	0.053416803
101114695	ARID5A	0.399386868	0.252867647	0.990459069	0.0000000358
101115720	MCRIP1	0.438705436	0.204680625	0.990412758	0.0000000365
101103676	INF2	0.495979644	0.144853331	0.990368972	0.0000000372
101115705	CFD	0.441466256	0.20151459	0.990193055	0.00000004
101116066	NAXD	0.474773346	0.165574121	0.990189816	0.00000004
101121189	C13H20orf27	0.406321769	0.243949686	0.990159589	0.0000000405
101102967	LOC101102967	0.605332542	0.063675797	0.663983055	0.036281541
101111462	PLEC	0.500855691	0.140324957	0.990150712	0.0000000407
101118537	LOC101118537	0.500855691	0.140324957	0.990150712	0.0000000407
101105453	SAC3D1	0.500855691	0.140324957	0.990150712	0.0000000407
101110073	MAPK7	0.49890327	0.142127621	0.990148772	0.0000000407
105616381	LOC105616381	0.603310066	0.064807505	0.344322846	0.329919614
105603009	-	0.603166122	0.06488855	0.625402965	0.053142677
105603532	-	0.603166122	0.06488855	0.625402965	0.053142677
101120874	GSTP1	0.397641375	0.255140351	0.990074217	0.000000042
101113512	ITPKB	0.423864931	0.222189424	0.989937846	0.0000000443
105604257	LOC105604257	0.600766718	0.066249281	0.650508951	0.041684446
101120122	TSSC4	0.48785925	0.152590137	0.989876009	0.0000000454
101116327	CARS2	0.540567668	0.106680358	0.989816234	0.0000000465

100270712	FABP5	0.599514339	0.066966878	0.699612672	0.024318915
101109175	REX1BD	0.433302495	0.210959373	0.989745175	0.0000000478
101119138	SH3TC1	0.421780508	0.224714729	0.989683091	0.000000049
101120439	SLC43A1	0.450172974	0.191717474	0.989632059	0.0000000499
101106717	SIVA1	0.453158469	0.188423649	0.989478314	0.0000000529
101112013	LOC101112013	0.490292308	0.150246336	0.989460193	0.0000000533
101115992	SDF2L1	0.457332671	0.183874472	0.989416621	0.0000000542
101103507	MRPL36	0.389393883	0.266031146	0.989302576	0.0000000566
101107225	GAS2L1	0.478889714	0.161420946	0.989297032	0.0000000567
101111258	RAP2C	-0.592737738	0.070937956	-0.84023947	0.00233922
101110355	JOSD2	0.462672099	0.178150741	0.989179241	0.0000000592
101106762	LCN6	0.507322041	0.134454911	0.989156787	0.0000000597
106991959	LOC106991959	0.507322041	0.134454911	0.989156787	0.0000000597
101107688	C1S	0.507322041	0.134454911	0.989156787	0.0000000597
101104132	-	0.507322041	0.134454911	0.989156787	0.0000000597
101122385	TRMT2A	0.456490314	0.18478723	0.989145964	0.0000000599
101112952	LOC101112952	0.587635911	0.074026693	0.428672493	0.216427039
101109325	BCAS2	-0.587232181	0.074274781	-0.544390533	0.103741158
101103150	LOC101103150	0.49177474	0.148829066	0.989127436	0.0000000603
101113686	HDAC5	0.394265787	0.259567467	0.989110125	0.0000000607
101109472	DBNDD2	0.586033899	0.075014291	0.539803991	0.107273727
101120817	-	0.488990733	0.151497426	0.988899657	0.0000000655
105612994	LOC105612994	0.446742488	0.195543585	0.988895042	0.0000000657
101120377	MPG	0.415641136	0.232247064	0.988856045	0.0000000666
101115250	YPEL3	0.404856219	0.24581944	0.988817002	0.0000000675
100305129	MRPL32	-0.582773444	0.077050587	-0.797520962	0.005713219
101114563	ZXDC	0.42009721	0.226765931	0.988757544	0.000000069
443117	JUNB	0.396871944	0.256145762	0.988505933	0.0000000753
101115745	DUSP23	0.4073762	0.242609379	0.988466668	0.0000000763
101102104	GATD1	0.462203176	0.178649129	0.988436415	0.0000000771
101109841	CORO1B	0.428352939	0.216807372	0.988239039	0.0000000825
101113072	LOC101113072	0.415913069	0.231910451	0.988144216	0.0000000852
101103806	MARCHF2	0.438228066	0.205230962	0.988013221	0.000000089

101105213	FAM207A	0.50010424	0.141017103	0.987910516	0.0000000921
101107260	LOC101107260	0.476157025	0.164171006	0.98779404	0.0000000957
101113423	NT5M	0.374352467	0.286536393	0.987741086	0.0000000974
101116329	AMZ2	0.57889797	0.079517129	0.680236932	0.030420103
101119489	PARK7	0.57862431	0.079693202	0.426159726	0.219428062
101112159	CHST1	0.487283801	0.153147692	0.987728939	0.0000000977
101108492	SGPP1	-0.577361683	0.08050884	-0.778788621	0.007943553
101104370	CHMP5	-0.577317856	0.080537248	-0.750977194	0.012299769
105614924	-	0.43804013	0.205447859	0.987541909	0.000000104
101107594	COPE	0.463980938	0.176764022	0.987515228	0.000000105
101109968	ALKBH7	0.392237918	0.262247266	0.987512777	0.000000105
101112474	LOC101112474	0.454869715	0.186550766	0.987501558	0.000000105
101110655	NECAP2	0.575219681	0.081904828	0.647560066	0.042934969
101110136	EP300	-0.57488106	0.082126933	-0.74884379	0.012690933
101119103	NUDT1	0.500286393	0.140849135	0.987442642	0.000000107
101115229	NCAPD3	0.574011773	0.082698882	0.631180959	0.050340229
101117189	EPN1	0.478729907	0.161581	0.98744242	0.000000107
101117762	ADAT3	0.484549038	0.155814258	0.987221905	0.000000115
101108361	LOC101108361	0.500590223	0.140569236	0.987163932	0.000000117
101110110	SLC19A1	0.501251395	0.139961319	0.987132738	0.000000118
101112458	TMEM18	0.35445834	0.314917192	0.986926541	0.000000126
101114511	AGPAT2	0.523772928	0.120211381	0.986920021	0.000000126
101106791	LOC101106791	0.464508493	0.176206892	0.986914004	0.000000126
101115122	MVB12A	0.474049203	0.166311289	0.98686448	0.000000128
101117328	CLTB	0.437198994	0.206420241	0.986819966	0.00000013
101121000	ERC1	0.571074993	0.084650096	0.54934676	0.100007499
101123490	BBC3	0.521107688	0.122452107	0.986776638	0.000000132
101120239	CSNK1G2	0.484534415	0.155828591	0.986707293	0.000000134
101114329	MOSMO	0.569485086	0.085718679	0.407816049	0.242051501
101108076	TFPT	0.532713586	0.112882192	0.986704727	0.000000135
105604619	FLYWCH1	0.509774703	0.132268628	0.986673383	0.000000136
101103837	ABTB2	0.44103871	0.202003016	0.986518767	0.000000142
101117254	GSDMD	0.391208721	0.263613134	0.986488035	0.000000143

101123281	LOC101123281	0.45191203	0.189794744	0.986364566	0.000000149
101116740	RAB20	0.391371509	0.263396834	0.986282377	0.000000152
100294652	AKT1	0.42556592	0.220140706	0.986277944	0.000000153
101104932	UCP2	0.389655818	0.265681406	0.986261487	0.000000153
101123002	EMILIN2	0.528335208	0.116435417	0.986232205	0.000000155
101106700	RPL38	0.564822082	0.088902533	0.541323072	0.106095458
105611679	-	0.500474328	0.140675962	0.986208545	0.000000156
101121851	NDUFB7	0.397169809	0.255756284	0.986190981	0.000000156
443417	TGFB1	0.414442582	0.233733985	0.986190573	0.000000156
101110745	PPP1R16A	0.516209962	0.126636996	0.986179623	0.000000157
101122898	NKAPL	0.562277346	0.090671546	0.50827451	0.133603266
105613844	-	0.516209962	0.126636996	0.986179623	0.000000157
101102849	VARS1	0.560995236	0.091571286	0.698965771	0.02450773
101118553	CEBPA	0.516209962	0.126636996	0.986179623	0.000000157
101116724	-	0.559934432	0.092320018	0.508837004	0.133101879
100174903	NR1D1	0.47111149	0.169321962	0.986164393	0.000000158
101118551	PLD3	0.520922872	0.122608441	0.986105579	0.00000016
101113996	SYTL1	0.512146159	0.130175698	0.98581767	0.000000174
101120606	MZT2B	0.456919073	0.184322304	0.985797745	0.000000175
101123545	EHMT1	0.45948071	0.181558966	0.985768619	0.000000176
101110230	LOC101110230	0.472311204	0.168088551	0.985583317	0.000000186
105608442	LOC105608442	0.420490887	0.226285262	0.985515742	0.000000189
101106011	BDH2	-0.555674369	0.095366148	-0.547418019	0.101450178
101116642	MCAT	0.49420351	0.146524673	0.985398101	0.000000195
101118997	SACM1L	-0.555341448	0.09560686	-0.655866468	0.039475565
101114619	GRINA	0.413757934	0.234585762	0.985361068	0.000000197
101121289	KMT5C	0.490998886	0.149569799	0.985308024	0.0000002
101119171	ATG2A	0.426000687	0.2196188	0.985211351	0.000000206
101110633	TMEM134	0.487051917	0.153372714	0.98517423	0.000000208
101111647	CSRP1	0.491449288	0.149139514	0.985169755	0.000000208
105614996	LOC105614996	0.432564477	0.211825527	0.985167893	0.000000208
105607861	LOC105607861	0.464352033	0.176372014	0.985099653	0.000000212
101105692	GPR132	0.501828407	0.139432101	0.984980895	0.000000219

105614076	LOC105614076	0.55316502	0.097190013	0.680694005	0.030265302
101122706	TMEM11	0.450115071	0.191781688	0.984936991	0.000000221
101103231	MRPL55	0.416607948	0.231051549	0.984926166	0.000000222
101109655	LOC101109655	0.437593137	0.205964268	0.984892441	0.000000224
101108527	ANKRD13D	0.478058118	0.162254876	0.984793482	0.00000023
101107304	MBOAT7	0.376048446	0.284183031	0.984786819	0.00000023
101119304	LTC4S	0.458637604	0.182465738	0.984698667	0.000000235
101106012	PDGFRA	-0.550886618	0.09886509	-0.788775184	0.006689788
101123600	CREBBP	-0.550765868	0.098954373	-0.78107117	0.007643452
101114566	RANBP9	-0.550701668	0.099001863	-0.692088748	0.026577564
101111994	RPS5	0.397670355	0.255102526	0.984697935	0.000000235
101105105	CRYBA4	0.548964999	0.100292014	0.659169268	0.038153895
101120012	ISOC2	0.437928998	0.205576179	0.984526818	0.000000246
105615377	LOC105615377	0.510369587	0.131741677	0.984442717	0.000000252
105610494	MISP3	0.480045404	0.160266299	0.984434928	0.000000252
101112814	YJU2	0.548173258	0.100883707	0.677282832	0.031433624
101108171	LOC101108171	0.476046945	0.16428237	0.98434685	0.000000258
101105488	EDF1	0.498278383	0.142707551	0.984166332	0.00000027
101112263	RIN3	0.439254484	0.204048711	0.984154977	0.000000271
105611364	-	0.473792452	0.166573129	0.984085521	0.000000275
101115666	AVPI1	0.436785056	0.206899742	0.983977325	0.000000283
101103115	MCRIP2	0.41288729	0.235671464	0.98396632	0.000000284
101118722	MON1B	0.474889536	0.165456023	0.983922391	0.000000287
101103418	NOP53	0.472356156	0.168042441	0.983861499	0.000000291
101121558	PTGDR2	0.421147884	0.225484377	0.983825308	0.000000294
101101937	SIPA1	0.388848322	0.266760397	0.983671748	0.000000305
100190977	CKB	0.422895467	0.223361919	0.983668026	0.000000305
101117865	ROMO1	0.456515804	0.184759571	0.983605285	0.00000031
101109080	-	0.407755312	0.242128493	0.983555115	0.000000314
101103616	LOC101103616	0.473058605	0.16732288	0.983516461	0.000000317
101119721	LOC101119721	0.484142228	0.156213302	0.983351615	0.000000329
101122954	CEBPE	0.453443727	0.188110683	0.983300226	0.000000333
101121678	LEAP2	-0.543874027	0.104135246	-0.653456242	0.040459284

101116560	CARD9	0.40954135	0.239870181	0.983296229	0.000000334
101104451	ANKMY1	0.462598377	0.17822904	0.983100284	0.00000035
443466	PRLH	0.462598377	0.17822904	0.983100284	0.00000035
443051	COL6A3	0.462598377	0.17822904	0.983100284	0.00000035
101113454	NGEF	0.462598377	0.17822904	0.983100284	0.00000035
105615733	LOC105615733	0.462598377	0.17822904	0.983100284	0.00000035
101111757	PDE4B	-0.541122547	0.106250524	-0.858867621	0.001458715
101105789	S100A16	0.462598377	0.17822904	0.983100284	0.00000035
106990787	-	0.462598377	0.17822904	0.983100284	0.00000035
101106557	NES	0.462598377	0.17822904	0.983100284	0.00000035
101108266	INSRR	0.462598377	0.17822904	0.983100284	0.00000035
105608968	ETV3L	0.462598377	0.17822904	0.983100284	0.00000035
101116526	IGSF9	0.462598377	0.17822904	0.983100284	0.00000035
105612162	LOC105612162	0.462598377	0.17822904	0.983100284	0.00000035
101123569	SUPT4H1	0.5377499	0.108880032	0.643032484	0.044903539
105606224	LOC105606224	0.537313907	0.109222915	0.678259354	0.031096086
101106565	ADAMTS4	0.462598377	0.17822904	0.983100284	0.00000035
443370	POU1F1	0.462598377	0.17822904	0.983100284	0.00000035
101120424	RNF213	-0.536569592	0.109809841	-0.609614885	0.061322529
101120035	TMPRSS7	0.462598377	0.17822904	0.983100284	0.00000035
105605206	LOC105605206	0.462598377	0.17822904	0.983100284	0.00000035
105602871	-	0.534838195	0.111182785	0.428641271	0.216464182
101113459	SVEP1	0.462598377	0.17822904	0.983100284	0.00000035
101120217	CCL19	0.462598377	0.17822904	0.983100284	0.00000035
101122581	LGI3	0.462598377	0.17822904	0.983100284	0.00000035
101116452	-	0.462598377	0.17822904	0.983100284	0.00000035
101103123	TRPM3	0.462598377	0.17822904	0.983100284	0.00000035
101107766	SCARA3	0.462598377	0.17822904	0.983100284	0.00000035
101107834	FILIP1L	0.533974342	0.111871803	0.675350425	0.032108894
101115683	PNOC	0.462598377	0.17822904	0.983100284	0.00000035
101110914	XKR6	0.462598377	0.17822904	0.983100284	0.00000035
105610586	-	0.462598377	0.17822904	0.983100284	0.00000035
105601905	-	0.462598377	0.17822904	0.983100284	0.00000035

101111646	SLC26A9	0.532665286	0.112921014	0.694389718	0.025872194
101109421	AKR7A2	0.462598377	0.17822904	0.983100284	0.00000035
101109254	CROCC	0.462598377	0.17822904	0.983100284	0.00000035
106990978	-	0.462598377	0.17822904	0.983100284	0.00000035
101116818	GPSM1	0.462598377	0.17822904	0.983100284	0.00000035
101115521	NTMT1	0.462598377	0.17822904	0.983100284	0.00000035
101107182	WDR34	0.462598377	0.17822904	0.983100284	0.00000035
443143	ITGAV	-0.530503652	0.114667026	-0.813536018	0.004194896
105607466	LOC105607466	0.462598377	0.17822904	0.983100284	0.00000035
443212	POMC	0.462598377	0.17822904	0.983100284	0.00000035
101113576	TOGARAM2	0.462598377	0.17822904	0.983100284	0.00000035
101108731	SMYD1	0.462598377	0.17822904	0.983100284	0.00000035
106991019	LOC106991019	0.462598377	0.17822904	0.983100284	0.00000035
101111446	LOC101111446	0.462598377	0.17822904	0.983100284	0.00000035
101114768	KCNK12	0.462598377	0.17822904	0.983100284	0.00000035
101116471	PROM2	0.462598377	0.17822904	0.983100284	0.00000035
105611974	-	0.462598377	0.17822904	0.983100284	0.00000035
105614699	LOC105614699	0.462598377	0.17822904	0.983100284	0.00000035
101107946	ALX1	0.462598377	0.17822904	0.983100284	0.00000035
105614796	-	0.462598377	0.17822904	0.983100284	0.00000035
101105069	LOC101105069	0.462598377	0.17822904	0.983100284	0.00000035
101104343	PTGER4	-0.527594884	0.117043038	-0.6308705	0.05048825
106991037	LOC106991037	0.462598377	0.17822904	0.983100284	0.00000035
101112975	TEAD4	0.462598377	0.17822904	0.983100284	0.00000035
101114265	DDX11	0.462598377	0.17822904	0.983100284	0.00000035
101111621	CSDC2	0.462598377	0.17822904	0.983100284	0.00000035
101113279	LOC101113279	0.526749374	0.117739408	0.570427191	0.085084446
101105651	LOC101105651	0.462598377	0.17822904	0.983100284	0.00000035
100037704	FBLN1	0.462598377	0.17822904	0.983100284	0.00000035
101113671	LOC101113671	0.462598377	0.17822904	0.983100284	0.00000035
101114186	MLC1	0.462598377	0.17822904	0.983100284	0.00000035
101117495	LMF2	0.462598377	0.17822904	0.983100284	0.00000035
105609617	LOC105609617	0.462598377	0.17822904	0.983100284	0.00000035

101123549	LOC101123549	0.462598377	0.17822904	0.983100284	0.00000035
101104655	FLNC	0.462598377	0.17822904	0.983100284	0.00000035
105609822	LOC105609822	0.462598377	0.17822904	0.983100284	0.00000035
101104760	LACTB2	-0.525359719	0.118889548	-0.875497127	0.000902171
101104575	YJEFN3	0.462598377	0.17822904	0.983100284	0.00000035
101107353	GDF1	0.462598377	0.17822904	0.983100284	0.00000035
101108387	KLHL26	0.462598377	0.17822904	0.983100284	0.00000035
443102	JUND	0.462598377	0.17822904	0.983100284	0.00000035
101111628	LOC101111628	0.462598377	0.17822904	0.983100284	0.00000035
102465821	MIR23A	0.462598377	0.17822904	0.983100284	0.00000035
102465824	MIR27A	0.462598377	0.17822904	0.983100284	0.00000035
101103719	NFIX	0.462598377	0.17822904	0.983100284	0.00000035
101120080	TSPAN16	0.462598377	0.17822904	0.983100284	0.00000035
443517	CNN1	0.462598377	0.17822904	0.983100284	0.00000035
101121854	LOC101121854	0.462598377	0.17822904	0.983100284	0.00000035
101112564	FSD1	0.462598377	0.17822904	0.983100284	0.00000035
101101785	-	0.523631465	0.120329666	0.698461677	0.024655559
101116907	ZFR2	0.462598377	0.17822904	0.983100284	0.00000035
105608683	LOC105608683	0.462598377	0.17822904	0.983100284	0.00000035
101122946	PRR7	0.462598377	0.17822904	0.983100284	0.00000035
101101781	-	0.462598377	0.17822904	0.983100284	0.00000035
101115458	APC2	0.462598377	0.17822904	0.983100284	0.00000035
101109876	GFRA3	0.462598377	0.17822904	0.983100284	0.00000035
101115622	C1QTNF2	0.462598377	0.17822904	0.983100284	0.00000035
105613852	-	0.462598377	0.17822904	0.983100284	0.00000035
101111286	LOC101111286	0.462598377	0.17822904	0.983100284	0.00000035
101112060	LOC101112060	0.462598377	0.17822904	0.983100284	0.00000035
101123558	DMP1	0.462598377	0.17822904	0.983100284	0.00000035
106991243	-	0.462598377	0.17822904	0.983100284	0.00000035
101103648	NSD2	0.462598377	0.17822904	0.983100284	0.00000035
101113761	LOC101113761	0.462598377	0.17822904	0.983100284	0.00000035
101114446	FOXB1	0.462598377	0.17822904	0.983100284	0.00000035
105615794	-	0.462598377	0.17822904	0.983100284	0.00000035

105611043	-	0.462598377	0.17822904	0.983100284	0.00000035
105611045	-	0.462598377	0.17822904	0.983100284	0.00000035
101111811	LOC101111811	0.462598377	0.17822904	0.983100284	0.00000035
100620047	BTG2	-0.519189243	0.124080939	-0.398142138	0.254487183
105615913	-	0.462598377	0.17822904	0.983100284	0.00000035
101107530	LOC101107530	0.462598377	0.17822904	0.983100284	0.00000035
105615969	-	0.462598377	0.17822904	0.983100284	0.00000035
101118030	C9H8orf82	0.462598377	0.17822904	0.983100284	0.00000035
105615980	-	0.462598377	0.17822904	0.983100284	0.00000035
101113248	TIGD5	0.462598377	0.17822904	0.983100284	0.00000035
101119647	THEM6	0.462598377	0.17822904	0.983100284	0.00000035
101117854	TSNARE1	0.462598377	0.17822904	0.983100284	0.00000035
101110246	ZFHX4	0.462598377	0.17822904	0.983100284	0.00000035
101114786	CALB1	0.462598377	0.17822904	0.983100284	0.00000035
101109190	OXGR1	0.462598377	0.17822904	0.983100284	0.00000035
105616226	-	0.462598377	0.17822904	0.983100284	0.00000035
105616295	-	0.462598377	0.17822904	0.983100284	0.00000035
101112746	SMTNL2	0.462598377	0.17822904	0.983100284	0.00000035
105607800	NTN1	0.462598377	0.17822904	0.983100284	0.00000035
101104413	MYOCD	0.462598377	0.17822904	0.983100284	0.00000035
106991422	LOC106991422	0.462598377	0.17822904	0.983100284	0.00000035
101111562	NXPH3	0.462598377	0.17822904	0.983100284	0.00000035
105610170	LOC105610170	0.462598377	0.17822904	0.983100284	0.00000035
100141298	KRT27	0.462598377	0.17822904	0.983100284	0.00000035
101118459	LOC101118459	0.462598377	0.17822904	0.983100284	0.00000035
101110525	CAVIN1	0.462598377	0.17822904	0.983100284	0.00000035
101105588	LAMC1	0.513702109	0.128813673	0.503999456	0.137451874
106991404	-	0.462598377	0.17822904	0.983100284	0.00000035
105616467	TMEM235	0.462598377	0.17822904	0.983100284	0.00000035
101116847	UBE2O	0.462598377	0.17822904	0.983100284	0.00000035
101120933	CASKIN2	0.462598377	0.17822904	0.983100284	0.00000035
101123478	HID1	0.462598377	0.17822904	0.983100284	0.00000035
101111414	SMAD7	0.512252095	0.130082684	0.63893361	0.046737016

101102657	FADS6	0.462598377	0.17822904	0.983100284	0.00000035
101108503	LOC101108503	0.462598377	0.17822904	0.983100284	0.00000035
101122042	GPR153	0.462598377	0.17822904	0.983100284	0.00000035
101123315	TMEM52	0.462598377	0.17822904	0.983100284	0.00000035
101117093	LOC101117093	0.462598377	0.17822904	0.983100284	0.00000035
101109907	MPEG1	0.51013275	0.131951313	0.491904612	0.148705291
101112408	PTPN14	0.462598377	0.17822904	0.983100284	0.00000035
101115892	FRMD4A	0.462598377	0.17822904	0.983100284	0.00000035
443477	AVP	0.462598377	0.17822904	0.983100284	0.00000035
105616788	C13H20orf204	0.462598377	0.17822904	0.983100284	0.00000035
101117352	TNFRSF6B	0.462598377	0.17822904	0.983100284	0.00000035
101110435	BIRC7	0.462598377	0.17822904	0.983100284	0.00000035
101109469	SNTA1	0.462598377	0.17822904	0.983100284	0.00000035
101120595	LOC101120595	0.462598377	0.17822904	0.983100284	0.00000035
101106450	LPIN3	0.462598377	0.17822904	0.983100284	0.00000035
100127216	TNNC2	0.462598377	0.17822904	0.983100284	0.00000035
101123359	EI24	0.507854914	0.133978035	0.617891242	0.056938124
101107201	TBPL1	-0.507645097	0.134165679	-0.761359302	0.01051607
101117693	BCAR1	0.462598377	0.17822904	0.983100284	0.00000035
101106278	ZFPM1	0.462598377	0.17822904	0.983100284	0.00000035
101109036	RGS9BP	0.462598377	0.17822904	0.983100284	0.00000035
101122717	LOC101122717	0.462598377	0.17822904	0.983100284	0.00000035
106991536	-	0.462598377	0.17822904	0.983100284	0.00000035
101115152	PAK4	0.462598377	0.17822904	0.983100284	0.00000035
101105272	LOC101105272	0.462598377	0.17822904	0.983100284	0.00000035
101107624	-	0.462598377	0.17822904	0.983100284	0.00000035
101115564	-	0.462598377	0.17822904	0.983100284	0.00000035
101103177	EHD2	0.462598377	0.17822904	0.983100284	0.00000035
101121794	SYNGR4	0.462598377	0.17822904	0.983100284	0.00000035
101116251	KCNC3	0.462598377	0.17822904	0.983100284	0.00000035
101119583	ACP4	0.462598377	0.17822904	0.983100284	0.00000035
101119751	ZNF628	0.462598377	0.17822904	0.983100284	0.00000035
105601888	LOC105601888	0.505533919	0.136062747	0.40147077	0.250169096

101102936	WDR20	-0.50551144	0.136083034	-0.518238123	0.124893437
101117205	DOCK1	0.505104257	0.136450841	0.698749167	0.024571176
101111997	ZIM2	0.462598377	0.17822904	0.983100284	0.00000035
101122381	LOC101122381	0.462598377	0.17822904	0.983100284	0.00000035
105602149	-	0.462598377	0.17822904	0.983100284	0.00000035
101122219	HSPA4L	-0.504303163	0.137176245	-0.622720588	0.054478124
101107895	HSPB2	0.462598377	0.17822904	0.983100284	0.00000035
101109209	TTC12	0.462598377	0.17822904	0.983100284	0.00000035
101120522	ABCC8	0.462598377	0.17822904	0.983100284	0.00000035
101112085	OLFML1	0.462598377	0.17822904	0.983100284	0.00000035
101102175	LOC101102175	0.462598377	0.17822904	0.983100284	0.00000035
101123662	LOC101123662	0.462598377	0.17822904	0.983100284	0.00000035
101113882	CREB3L1	0.462598377	0.17822904	0.983100284	0.00000035
106991626	-	0.462598377	0.17822904	0.983100284	0.00000035
101119671	TNKS1BP1	0.462598377	0.17822904	0.983100284	0.00000035
105611415	RTN4RL2	0.462598377	0.17822904	0.983100284	0.00000035
101103182	LOC101103182	0.462598377	0.17822904	0.983100284	0.00000035
101105358	RGS7BP	0.462598377	0.17822904	0.983100284	0.00000035
105602559	LOC105602559	0.462598377	0.17822904	0.983100284	0.00000035
101110873	LOC101110873	0.462598377	0.17822904	0.983100284	0.00000035
443306	FGF2	0.462598377	0.17822904	0.983100284	0.00000035
101108257	-	0.462598377	0.17822904	0.983100284	0.00000035
101117536	LOC101117536	0.462598377	0.17822904	0.983100284	0.00000035
105603291	-	0.462598377	0.17822904	0.983100284	0.00000035
101102516	-	0.462598377	0.17822904	0.983100284	0.00000035
101113627	LOC101113627	0.462598377	0.17822904	0.983100284	0.00000035
101114142	ADAMTS7	0.462598377	0.17822904	0.983100284	0.00000035
101103270	ISL2	0.462598377	0.17822904	0.983100284	0.00000035
101111492	GPR68	0.462598377	0.17822904	0.983100284	0.00000035
101114907	CHGA	0.462598377	0.17822904	0.983100284	0.00000035
105603225	LOC105603225	0.462598377	0.17822904	0.983100284	0.00000035
105601847	-	0.462598377	0.17822904	0.983100284	0.00000035
101102182	TRMT61A	0.462598377	0.17822904	0.983100284	0.00000035

101108429	PXK	-0.50041931	0.140726645	-0.651330643	0.041340404
105603311	XRCC3	0.462598377	0.17822904	0.983100284	0.00000035
105603281	AHNAK2	0.462598377	0.17822904	0.983100284	0.00000035
105610561	LOC105610561	0.462598377	0.17822904	0.983100284	0.00000035
101112092	TTC21A	0.462598377	0.17822904	0.983100284	0.00000035
101119338	ZBTB47	0.462598377	0.17822904	0.983100284	0.00000035
101119258	C19H3orf86	0.462598377	0.17822904	0.983100284	0.00000035
105603438	-	0.462598377	0.17822904	0.983100284	0.00000035
101119261	COL7A1	0.462598377	0.17822904	0.983100284	0.00000035
101112006	EFCC1	0.462598377	0.17822904	0.983100284	0.00000035
101114819	SLC41A3	0.462598377	0.17822904	0.983100284	0.00000035
105603655	-	0.462598377	0.17822904	0.983100284	0.00000035
101103274	DAAM2	0.462598377	0.17822904	0.983100284	0.00000035
105603708	LOC105603708	0.462598377	0.17822904	0.983100284	0.00000035
101106292	LOC101106292	0.462598377	0.17822904	0.983100284	0.00000035
101105036	LOC101105036	0.462598377	0.17822904	0.983100284	0.00000035
101110711	LOC101110711	0.462598377	0.17822904	0.983100284	0.00000035
105603946	-	0.462598377	0.17822904	0.983100284	0.00000035
106990154	-	0.462598377	0.17822904	0.983100284	0.00000035
105604163	LOC105604163	0.462598377	0.17822904	0.983100284	0.00000035
101120120	ANO5	0.462598377	0.17822904	0.983100284	0.00000035
101116360	KCNK4	0.462598377	0.17822904	0.983100284	0.00000035
101108175	SLC25A45	0.462598377	0.17822904	0.983100284	0.00000035
101121388	SYT8	0.462598377	0.17822904	0.983100284	0.00000035
106991827	TOLLIP	0.462598377	0.17822904	0.983100284	0.00000035
101116947	INSYN2A	0.462598377	0.17822904	0.983100284	0.00000035
101109756	-	0.462598377	0.17822904	0.983100284	0.00000035
101105461	GNAL	0.462598377	0.17822904	0.983100284	0.00000035
101123172	LOC101123172	0.462598377	0.17822904	0.983100284	0.00000035
101120129	SERPINB13	0.462598377	0.17822904	0.983100284	0.00000035
105604659	LOC105604659	0.462598377	0.17822904	0.983100284	0.00000035
101112971	LRP6	0.496667382	0.144209307	0.548607045	0.100559251
105604700	-	0.462598377	0.17822904	0.983100284	0.00000035

101104957	FBXL19	0.462598377	0.17822904	0.983100284	0.00000035
101114236	FZD9	0.462598377	0.17822904	0.983100284	0.00000035
101114746	LOC101114746	0.462598377	0.17822904	0.983100284	0.00000035
101117815	SH2B2	0.462598377	0.17822904	0.983100284	0.00000035
101114925	GPR146	0.462598377	0.17822904	0.983100284	0.00000035
101121567	ADO	0.462598377	0.17822904	0.983100284	0.00000035
101122571	LOC101122571	0.462598377	0.17822904	0.983100284	0.00000035
101107841	HKDC1	0.462598377	0.17822904	0.983100284	0.00000035
100145877	RIT1	-0.496132338	0.144710192	-0.71037213	0.021323348
101106391	PLAC9	0.462598377	0.17822904	0.983100284	0.00000035
101102872	MYOM2	0.462598377	0.17822904	0.983100284	0.00000035
101119723	PCYOX1L	0.495932276	0.144897753	0.691090007	0.026887787
105601914	-	0.462598377	0.17822904	0.983100284	0.00000035
105611964	ASMTL	0.462598377	0.17822904	0.983100284	0.00000035
101102784	LOC101102784	0.462598377	0.17822904	0.983100284	0.00000035
101120990	EDA	0.462598377	0.17822904	0.983100284	0.00000035
101105981	RTL8C	0.462598377	0.17822904	0.983100284	0.00000035
101113311	CHRDL1	0.462598377	0.17822904	0.983100284	0.00000035
100233168	BSP5L	0.462598377	0.17822904	0.983100284	0.00000035
101116713	-	0.462598377	0.17822904	0.983100284	0.00000035
101111169	ABCA2	0.462598377	0.17822904	0.983100284	0.00000035
101104154	REM2	-0.493490894	0.147198529	-0.564864223	0.088873425
105605780	LOC105605780	0.462598377	0.17822904	0.983100284	0.00000035
101115103	-	0.462598377	0.17822904	0.983100284	0.00000035
101106314	-	0.462598377	0.17822904	0.983100284	0.00000035
105605880	-	0.462598377	0.17822904	0.983100284	0.00000035
105605891	-	0.462598377	0.17822904	0.983100284	0.00000035
101123615	-	0.462598377	0.17822904	0.983100284	0.00000035
105605954	LOC105605954	0.462598377	0.17822904	0.983100284	0.00000035
101104726	LOC101104726	0.462598377	0.17822904	0.983100284	0.00000035
101119804	-	0.462598377	0.17822904	0.983100284	0.00000035
101102799	SPSB2	0.462598377	0.17822904	0.983100284	0.00000035
105606098	-	0.462598377	0.17822904	0.983100284	0.00000035

105606140	-	0.462598377	0.17822904	0.983100284	0.00000035
101106245	MICALL2	0.462598377	0.17822904	0.983100284	0.00000035
101108597	KCNN4	0.496738345	0.144142954	0.983045324	0.000000354
101110154	TP53I13	0.447105456	0.195136666	0.983014518	0.000000357
101117761	S1PR5	0.52194845	0.121742479	0.982986853	0.000000359
105606118	-	0.458787944	0.18230385	0.982738964	0.00000038
101117529	EMC10	0.402464988	0.248887304	0.982653689	0.000000388
101117537	CRKL	0.481248644	0.159069441	0.982645489	0.000000389
101120352	UBE2S	0.435860157	0.207973461	0.982627659	0.00000039
101114121	ANAPC11	0.458441465	0.182677071	0.982443802	0.000000407
101114723	C15H11orf71	0.486338031	0.154066729	0.98240443	0.000000411
101122842	FAM160B2	0.477918136	0.162395504	0.982398006	0.000000411
101103935	RASSF7	0.400330531	0.251643656	0.982314498	0.000000419
100579169	GSN	0.377996548	0.281492738	0.982236322	0.000000426
106991688	-	0.454961055	0.186451107	0.982178594	0.000000432
101122286	LOC101122286	0.457847033	0.183318432	0.982096551	0.00000044
101115287	GLI4	0.499013487	0.142025483	0.982019741	0.000000447
443200	TJP1	-0.489108965	0.15138352	-0.716766543	0.019669327
101105714	PPP1R35	0.386038693	0.270533347	0.981906155	0.000000459
101114831	SPINDOC	0.472196586	0.168206156	0.981808037	0.000000469
101105148	IER5L	0.430106062	0.214725515	0.981788099	0.000000471
101114282	RHPN1	0.516797852	0.126130059	0.98172845	0.000000477
101117526	E2F1	0.50033416	0.140805107	0.981576743	0.000000493
101102078	LOC101102078	-0.487844384	0.152604524	-0.670475157	0.033856196
101107795	LOC101107795	-0.487593765	0.152847213	-0.53957011	0.107455864
101109908	DCHS1	0.50033416	0.140805107	0.981576743	0.000000493
101106385	TGFB1I1	0.50033416	0.140805107	0.981576743	0.000000493
101123196	-	0.50033416	0.140805107	0.981576743	0.000000493
101109641	ZNF787	0.50033416	0.140805107	0.981576743	0.000000493
101114495	AKAP17A	0.50033416	0.140805107	0.981576743	0.000000493
105609798	LOC105609798	0.50033416	0.140805107	0.981576743	0.000000493
101112869	NUBP2	0.436660028	0.2070447	0.9815193	0.000000499
101112926	ADORA2A	0.452055494	0.189636636	0.981426103	0.000000509

101114728	TOP3B	0.455610935	0.185742946	0.98126015	0.000000528
101105235	TRAF2	0.455610935	0.185742946	0.98126015	0.000000528
101112798	GFI1B	0.501979351	0.139293862	0.981038131	0.000000553
105603312	ZFYVE21	0.375101265	0.285496062	0.981004313	0.000000557
780454	FURIN	0.432029306	0.212454894	0.98065115	0.000000599
101108996	ATP13A2	0.521977691	0.121717845	0.980646784	0.0000006
101116998	ING1	0.471144703	0.169287743	0.980589185	0.000000607
101115236	LOC101115236	0.568316776	0.086509402	0.980496484	0.000000618
101122334	BMP1	0.385352197	0.271459624	0.980444414	0.000000625
101119750	BLVRB	0.350233796	0.321126161	0.980287149	0.000000645
101117502	ATP5F1D	0.42195498	0.22450273	0.98016341	0.000000661
101106539	ABHD12	0.377800052	0.281763468	0.98014933	0.000000663
101110527	LOC101110527	0.401919834	0.249589688	0.980102262	0.00000067
101101973	ARRDC1	0.474766111	0.165581477	0.979855677	0.000000703
101119378	RHOT2	0.540218337	0.106951527	0.979769678	0.000000715
101113335	LOC101113335	0.463486248	0.177287392	0.979757774	0.000000717
101111625	CERK	0.457076476	0.184151797	0.979626452	0.000000736
101109746	LOC101109746	0.482874635	0.15746065	0.691232661	0.026843326
101104038	CLN8	0.418539774	0.228673199	0.979552123	0.000000746
101111585	B3GALT4	0.449755555	0.192180671	0.979513808	0.000000752
101122673	ABCD1	0.594853071	0.069682331	0.979418639	0.000000766
101107836	CTDP1	0.466986549	0.173603879	0.979413823	0.000000766
101109923	NDUFC2	0.481944126	0.158380109	0.587993552	0.073807376
101120808	PGAP1	-0.481927162	0.158396901	-0.622502459	0.054587689
105605699	-	0.430140065	0.21468525	0.979267125	0.000000788
101115549	GPIHBP1	0.430140065	0.21468525	0.979267125	0.000000788
101119587	SMPD4	0.430140065	0.21468525	0.979267125	0.000000788
101116550	PLCXD1	0.430140065	0.21468525	0.979267125	0.000000788
105602000	TMEM238	0.430140065	0.21468525	0.979267125	0.000000788
101119391	RFX1	0.430140065	0.21468525	0.979267125	0.000000788
101110841	ELANE	0.519028962	0.12421763	0.979255723	0.00000079
101115834	PSMB9	0.428362301	0.216796224	0.979220813	0.000000795
101102135	MPST	0.51566428	0.127108664	0.979203019	0.000000798

101111426	EIF4EBP1	0.412652414	0.235964841	0.978942089	0.000000839
101115474	ACOT8	0.373549275	0.287654558	0.978935246	0.00000084
105612424	SSNA1	0.387388124	0.268717625	0.978754166	0.000000869
101102772	LOC101102772	0.393515228	0.260557552	0.978448367	0.00000092
101113558	ARPC1B	0.383798902	0.273561834	0.978347161	0.000000937
101105615	B4GALNT4	0.586575385	0.074679528	0.97832766	0.00000094
101111911	LOC101111911	0.405085325	0.245526618	0.978298952	0.000000945
443390	OXT	0.461331054	0.179578246	0.978041848	0.000000991
101119612	DOK2	0.426657452	0.218831743	0.978034496	0.000000992
101117439	FAM171A1	0.478524586	0.161786781	0.591478904	0.071692126
101106897	MGMT	0.47899301	0.16131754	0.977973159	0.000001
101111705	SLC2A6	0.381024077	0.277339233	0.977949638	0.00000101
101111737	ACTN4	0.337211803	0.340660865	0.977925495	0.00000101
100302072	RABAC1	0.361102248	0.305280925	0.977724587	0.00000105
101118219	ARHGAP1	0.523442982	0.120487378	0.977593948	0.00000107
105606828	-	0.51698997	0.125964669	0.977572871	0.00000108
100302099	CD93	0.492524226	0.148115627	0.977438735	0.0000011
101115274	B4GALNT3	0.528798794	0.116055935	0.977400559	0.00000111
101122371	DPT	0.528798794	0.116055935	0.977400559	0.00000111
105606890	LOC105606890	0.528798794	0.116055935	0.977400559	0.00000111
101111903	PRDM11	0.528798794	0.116055935	0.977400559	0.00000111
105604490	LOC105604490	0.528798794	0.116055935	0.977400559	0.00000111
101117012	POLD1	0.407503465	0.242447889	0.977354338	0.00000112
101119148	UNC13D	0.390636241	0.264374572	0.977312766	0.00000113
101104910	CTBP1	0.444783915	0.197747859	0.977312042	0.00000113
101118349	KCNT1	0.434914208	0.209074936	0.977211106	0.00000115
101101968	CAPG	0.385424721	0.271361686	0.977184079	0.00000115
101110452	SF3A1	0.475024252	0.165319159	0.484588792	0.155775296
101118471	ZNF524	0.471043854	0.169391657	0.976685058	0.00000126
101113469	EIPR1	0.467088959	0.1734968	0.976531535	0.00000129
101115557	LOC101115557	0.365656008	0.298767496	0.976447632	0.00000131
101115330	FERMT3	0.405471591	0.245033373	0.976378542	0.00000132
101112239	ENGASE	0.462331925	0.178512207	0.976212764	0.00000136

443003	ATP6V0C	0.49267533	0.147972044	0.976205374	0.00000136
101120652	TOM1	0.472004293	0.16840357	0.976178719	0.00000137
101116929	PSMB10	0.437458005	0.206120532	0.976067001	0.00000139
101118605	TUBA8	0.496536	0.144332203	0.975949323	0.00000142
101110884	CNPY3	0.404653905	0.246078178	0.975934627	0.00000143
100145878	BLOC1S4	0.485254108	0.155124107	0.975834273	0.00000145
101114915	ZBTB7B	0.419901296	0.227005353	0.975789153	0.00000146
101112854	PCBP4	0.525803456	0.118521534	0.975457718	0.00000154
101123013	RPE	-0.473749862	0.166616586	-0.713045556	0.020620591
101102074	SEPTIN9	0.563025436	0.090149184	0.975450865	0.00000154
101121665	TMEM187	0.357468673	0.310531505	0.975383413	0.00000156
106991188	-	0.424607294	0.221293969	0.975166485	0.00000161
101121516	PLEKHJ1	0.428527863	0.21659913	0.975141097	0.00000162
101119064	NGFR	0.370618184	0.291754938	0.975122071	0.00000163
101108672	TNFRSF18	0.408897321	0.240683139	0.975000096	0.00000166
100216476	HTT	0.55550617	0.095487713	0.974905576	0.00000168
101102696	QPRT	0.367166202	0.296623877	0.974888468	0.00000169
101112506	TCF25	0.48495213	0.155419469	0.974885612	0.00000169
101116427	CCDC92	0.534713882	0.111281773	0.974843071	0.0000017
101114205	RNPEP	0.569550585	0.085674486	0.974815939	0.00000171
101122205	LOC101122205	0.461938438	0.178930866	0.974806216	0.00000171
105609429	-	0.390608736	0.264411186	0.974758468	0.00000172
101105648	CRAT	0.451957769	0.189744328	0.974724292	0.00000173
101120676	MIEF2	0.346477515	0.326699951	0.97469631	0.00000174
105616536	FNDC10	0.526954714	0.117570051	0.974692141	0.00000174
101108086	LOC101108086	0.403790965	0.247183501	0.974649268	0.00000175
101115815	TCEA2	0.450410678	0.191453995	0.974537529	0.00000178
101107484	PC	0.506867656	0.134862369	0.974443247	0.00000181
105604611	-	0.477938804	0.162374736	0.974306977	0.00000185
101106688	GAK	0.437847466	0.205670351	0.974294289	0.00000185
101119679	LOC101119679	0.41932024	0.227716291	0.974237321	0.00000187
101119469	MBD3	0.451465671	0.190287163	0.974129152	0.0000019
101122599	PEX11G	0.384249392	0.272951236	0.974104919	0.00000191

101116964	LOC101116964	-0.469801197	0.170675212	-0.7294613	0.01665256
101112190	ABHD11	0.346560227	0.326576683	0.974091077	0.00000191
101113505	NAPRT	0.351424741	0.319369378	0.974075388	0.00000192
101106672	ZDHHC12	0.512637792	0.129744383	0.974017031	0.00000193
101105718	POFUT2	0.446279297	0.196063581	0.973821026	0.00000199
101111035	LOC101111035	0.474046201	0.166314349	0.973784172	0.000002
101103285	BCL7C	0.458296835	0.182832997	0.973586829	0.00000206
101114955	GAMT	0.492828366	0.14782671	0.973576997	0.00000207
101113325	SLC4A2	0.472105813	0.168299329	0.973559501	0.00000207
101106872	NOSIP	0.47143292	0.168990975	0.973378008	0.00000213
101113408	USHBP1	0.45599092	0.18532962	0.973352533	0.00000214
101122370	TBKBP1	0.43853685	0.204874882	0.972835721	0.00000231
101104521	FBXO31	0.499253448	0.141803266	0.972669637	0.00000236
101104150	SOD3	0.483674071	0.156673285	0.97256603	0.0000024
101112677	GUCD1	0.557324922	0.094178448	0.972499771	0.00000242
101114699	F2RL3	0.460193501	0.18079443	0.972419938	0.00000245
105610127	LOC105610127	0.330353768	0.351186488	0.972408445	0.00000245
101108289	WDTC1	0.432543685	0.211849958	0.9723363	0.00000248
101103232	LOC101103232	0.46369211	0.177069483	0.972332719	0.00000248
101112901	ARHGAP45	0.440458938	0.202666438	0.972190819	0.00000253
106991165	-	0.395313061	0.258189453	0.972092938	0.00000257
101114605	MYO1G	0.472559502	0.167833952	0.972023049	0.00000259
101107513	LOC101107513	0.38011862	0.278577934	0.971978238	0.00000261
101122855	LOC101122855	0.403847689	0.247110759	0.971863869	0.00000265
443048	DCN	0.500264732	0.140869103	0.971811314	0.00000267
101120572	ELOF1	0.444444309	0.198131533	0.971696578	0.00000271
105612923	LOC105612923	0.432746611	0.21161158	0.971644329	0.00000273
101115904	C15H11orf96	0.482250829	0.158076691	0.971509054	0.00000279
101117698	DGKZ	0.39359207	0.260456092	0.971476814	0.0000028
101122598	GUK1	0.52471962	0.119421669	0.97147312	0.0000028
101118542	SLC38A10	0.396950119	0.256043511	0.971402142	0.00000283
101114245	SYN1	0.410732141	0.238371113	0.971392855	0.00000283
101104538	SORT1	0.466747657	0.173853814	0.581765706	0.077687127

101121210	APOM	0.46595899	0.174680459	0.971300654	0.00000287
105605091	LOC105605091	0.504077649	0.137380877	0.971279031	0.00000288
106992016	-	0.518875503	0.124348589	0.971212301	0.0000029
101116237	COMMD5	0.341649545	0.333936909	0.971190137	0.00000291
101120936	SPEF1	0.521387979	0.122215248	0.971173977	0.00000292
101117260	OXLD1	0.394090574	0.25979841	0.971086294	0.00000295
101110140	IFFO1	0.440075665	0.203105705	0.970728324	0.0000031
101113846	MAP2K2	0.427740645	0.217537194	0.970679548	0.00000312
101111491	PXN	0.409435446	0.240003757	0.970604879	0.00000315
101104079	CST3	0.404462935	0.24632255	0.970572062	0.00000317
101116972	LOC101116972	0.355572645	0.313290023	0.970491948	0.0000032
101107475	LOC101107475	0.473944197	0.166418346	0.970474232	0.00000321
101106905	NINJ1	0.414846654	0.233232095	0.970465701	0.00000321
101121395	LRCH4	0.33810491	0.339302135	0.9704554	0.00000322
101116568	ABCA7	0.340291341	0.33598753	0.970412407	0.00000324
101114044	RIPOR1	0.541279507	0.106129134	0.970402719	0.00000324
101105208	LOC101105208	0.381861424	0.276196377	0.97034801	0.00000326
101119403	LAMP1	0.46942683	0.171063033	0.970271731	0.0000033
101122460	AP2S1	0.551370499	0.09850782	0.970202059	0.00000333
101107687	LOC101107687	0.367126998	0.296679421	0.970166457	0.00000334
101109130	LYRM4	0.514604206	0.128028059	0.97014925	0.00000335
101120934	-	0.448720104	0.193332502	0.96998854	0.00000342
100286806	-	0.479982726	0.160328793	0.969970251	0.00000343
101114706	GCNT3	0.412973124	0.235564302	0.969760221	0.00000353
101104145	GADD45GIP1	0.375765845	0.284574446	0.969514345	0.00000364
101121122	TANGO2	0.524623922	0.119501352	0.969470596	0.00000366
101112312	STAP2	0.465353132	0.175317076	0.968956023	0.00000391
100145857	BCKDHA	0.407595839	0.242330711	0.968940426	0.00000392
101122299	MLYCD	0.478052423	0.162260595	0.968892545	0.00000395
101108823	NDOR1	0.489246465	0.151251116	0.968785467	0.000004
101107290	MFSD3	0.343849888	0.330628526	0.968771121	0.00000401
105616427	SMIM5	0.381259574	0.277017556	0.968715884	0.00000404
101113936	GSTZ1	0.420768056	0.225947194	0.968655637	0.00000407

101121258	ZNF358	0.423281262	0.222894907	0.968638756	0.00000407
101114469	LOC101114469	0.428344098	0.2168179	0.96854786	0.00000412
101102814	LOC101102814	0.383323053	0.274207611	0.968481254	0.00000416
101123066	GP6	0.482125206	0.158200927	0.968376767	0.00000421
105612995	LOC105612995	0.467000584	0.173589201	0.96832217	0.00000424
101108184	TSR3	0.423633124	0.222469457	0.968282127	0.00000426
101102417	KPTN	0.464478724	0.176238302	0.968140145	0.00000434
780524	RPS2	0.393924914	0.260016866	0.967950002	0.00000444
101109632	PLEKHM2	0.434451164	0.209615336	0.967920438	0.00000446
101113240	LTB4R	0.305732936	0.390294875	0.967866949	0.00000449
101115547	GPR31	0.470923267	0.169515959	0.967582495	0.00000465
443445	THRB	0.470923267	0.169515959	0.967582495	0.00000465
101102094	CDHR4	0.423121243	0.223088545	0.96740992	0.00000474
101114132	VAC14	0.456564317	0.184706936	0.967244824	0.00000484
101109108	FAAP100	0.536853991	0.109585347	0.967177175	0.00000488
101120627	NPRL3	0.465085509	0.175598724	0.967081609	0.00000494
101115743	GAB2	0.38245969	0.275381401	0.966973271	0.000005
101111643	SLC16A3	0.29831182	0.402478968	0.966949306	0.00000502
101107698	WDR83	0.438570935	0.204835598	0.966736366	0.00000515
101120509	THAP3	0.428703144	0.216390577	0.966699865	0.00000517
106991439	-	0.399361322	0.252900826	0.966687112	0.00000518
101105263	SPHK1	0.405698658	0.244743678	0.966485155	0.0000053
105604105	MACROD1	0.364858282	0.29990313	0.966477898	0.00000531
101120711	VPS51	0.378720478	0.280496526	0.96646488	0.00000531
101107721	FAM110A	0.316149041	0.37350047	0.966233843	0.00000546
101109132	LOC101109132	0.389585493	0.26577528	0.966186023	0.00000549
101120561	-	0.508127154	0.133734806	0.966082223	0.00000556
101103463	-	0.39707469	0.255880623	0.966081406	0.00000556
101117069	HDHD5	0.44319145	0.199550717	0.965741995	0.00000578
101121484	RABEP2	0.458677593	0.182422669	0.96572695	0.00000579
101104955	TSC22D4	0.373755424	0.287367344	0.96565448	0.00000584
101116843	LOC101116843	0.573626095	0.08295346	0.965238216	0.00000613
101109819	TMCC2	0.394303909	0.259517235	0.965072994	0.00000624

101109782	SZRD1	0.505889292	0.135742269	0.965070036	0.00000624
101103799	PLA2G6	0.519568727	0.123757683	0.965031939	0.00000627
101121737	ZNF771	0.317376053	0.371545977	0.964973508	0.00000631
101112143	ADCK5	0.654370524	0.040084206	0.964958342	0.00000632
101115451	TUBGCP6	0.473237556	0.167139866	0.964813492	0.00000643
101114815	LIMK2	0.383122376	0.274480199	0.964798171	0.00000644
101105512	UBALD2	0.337144758	0.340762975	0.964773307	0.00000646
101114408	LOC101114408	0.424140751	0.221856483	0.964497783	0.00000666
101116942	INKA1	0.31027428	0.382928237	0.964390565	0.00000674
101106374	LOC101106374	0.525369854	0.118881135	0.964258517	0.00000684
101114029	ZC3H3	0.502393471	0.138915031	0.964183571	0.00000689
101111182	POLR3H	0.469374927	0.171116843	0.96416817	0.00000691
101103186	SMTN	0.449494483	0.192470706	0.964154875	0.00000692
101108868	LOC101108868	0.393597281	0.260449212	0.964082727	0.00000697
101118272	ATG4D	0.428764021	0.216318172	0.963979551	0.00000705
101111530	UBAC1	0.465799507	0.174847906	0.963659531	0.0000073
105602100	LOC105602100	0.394732085	0.258953407	0.963571843	0.00000737
494436	-	0.384397254	0.272750985	0.963523858	0.00000741
105614819	LOC105614819	0.571102117	0.084631941	0.963409587	0.0000075
101106039	VGLL4	0.464001193	0.176742611	0.963407567	0.0000075
101102760	MLXIP	0.374332556	0.286564083	0.963336602	0.00000756
101108216	ARHGAP8	0.521739723	0.121918412	0.963299143	0.00000759
101118268	NCLN	0.304125855	0.392918088	0.963292527	0.0000076
443130	CAPN1	0.428639806	0.216465925	0.96309741	0.00000776
105605712	-	0.441401	0.201589094	0.962822125	0.00000799
101113646	NTHL1	0.501769559	0.139486018	0.962699491	0.0000081
101113369	LOC101113369	0.366251905	0.297920678	0.962597387	0.00000818
101114722	NUP62	0.46078606	0.180160305	0.962391099	0.00000836
101106991	LAMTOR4	0.378500359	0.280799233	0.962330452	0.00000842
101105595	SDHAF1	0.503823786	0.137611458	0.962161393	0.00000857
101119691	PAGR1	0.355183919	0.313857159	0.962150023	0.00000858
100147798	CCND3	0.380200483	0.278465819	0.962115753	0.00000861
101107239	RCE1	0.494941495	0.145828811	0.962053569	0.00000866

101121135	TNNI2	0.318326179	0.370036015	0.961921742	0.00000878
101122437	KLF1	0.547912591	0.101078995	0.961811584	0.00000889
101102612	RAB40C	0.496412881	0.144447427	0.96173926	0.00000895
101119273	KREMEN2	0.532095322	0.11337976	0.961687626	0.000009
101103631	-	0.426532827	0.218980967	0.961627756	0.00000906
101119407	UNK	0.535821352	0.110401857	0.961604193	0.00000908
105613208	LOC105613208	0.435712119	0.208145616	0.961543671	0.00000913
101111571	NR1H2	0.498367649	0.142624618	0.961490154	0.00000918
101114252	PLPP7	0.440281332	0.202869923	0.96122304	0.00000944
100135441	TALDO1	0.319144211	0.368738421	0.961078755	0.00000958
101109794	MPV17L2	0.447958127	0.194182694	0.960947195	0.00000971
101109786	RAPGEF3	0.419389281	0.227631751	0.960696461	0.00000996
101120598	RPL28	0.436327274	0.207430782	0.960569486	0.0000101
101105898	ESPL1	0.441956974	0.200954844	0.96054841	0.0000101
101110952	CBX4	0.458135213	0.183007335	0.960527864	0.0000101
101116821	IL17RA	0.487141595	0.153285666	0.960261039	0.0000104
101109193	CRYBA1	0.380231044	0.27842397	0.96016119	0.0000105
101114702	NDUFS7	0.488483246	0.151986934	0.960136783	0.0000105
101108261	NDUFAF3	0.423962922	0.222071107	0.959954643	0.0000107
101122982	TBC1D10A	0.489148016	0.15134591	0.959823115	0.0000109
105603154	-	0.501289613	0.139926229	0.959796587	0.0000109
101106938	PLPP2	0.581611563	0.07778479	0.959426308	0.0000113
101120852	TOR3A	0.391311899	0.263476029	0.959362272	0.0000114
101113608	VAT1	0.524636963	0.119490491	0.95935347	0.0000114
101121920	-	0.41742488	0.23004409	0.959307342	0.0000114
100307048	SPARC	0.546905615	0.101835654	0.959260776	0.0000115
101108793	UBTD1	0.314465474	0.376190437	0.959060993	0.0000117
101107948	LOC101107948	0.4114301	0.237494919	0.958956051	0.0000118
101119818	DOCK6	0.54060637	0.106650342	0.958843961	0.0000119
101111799	UBXN6	0.386587745	0.269793766	0.958734513	0.0000121
101111395	NDUFS6	0.455056006	0.186347543	0.958426379	0.0000124
101108484	NDUFA11	0.356380517	0.312113089	0.958320604	0.0000126
101115027	DNM2	0.324480118	0.360330014	0.958064782	0.0000129

101116596	PPP1R13L	0.380225253	0.278431901	0.958011779	0.0000129
101106839	KCTD17	0.35141483	0.319383979	0.95781407	0.0000132
105605819	ADAM8	0.353478548	0.316351587	0.957792004	0.0000132
101108272	RHBDF1	0.406987754	0.243102659	0.957724469	0.0000133
101114562	ABHD14A	0.386636341	0.269728359	0.95762228	0.0000134
101106412	ASL	0.497886421	0.14307205	0.957526054	0.0000135
101114353	BSG	0.453221896	0.188354035	0.957289754	0.0000138
101103427	SELENOM	0.525749281	0.118566425	0.957042602	0.0000141
101106041	LOC101106041	0.448840352	0.193198532	0.957027176	0.0000142
101104764	NAA38	0.368143737	0.295240718	0.956635447	0.0000147
101113934	MIDN	0.525385403	0.118868227	0.956454949	0.0000149
101112562	DDA1	0.398727697	0.253724589	0.95628561	0.0000152
101120195	YDJC	0.630602515	0.050616252	0.956236278	0.0000152
101102751	LENG1	0.451120651	0.190668298	0.956199821	0.0000153
101113304	ANKAR	0.554925401	0.095908218	0.956162708	0.0000153
101118541	HEXIM2	0.430876522	0.213814228	0.956099084	0.0000154
101104277	MRPS25	0.495889039	0.144938308	0.956036156	0.0000155
101112123	MAPRE3	0.418110459	0.229200542	0.956025213	0.0000155
105606262	LOC105606262	0.24888633	0.488051137	0.955962303	0.0000156
443123	TSPO	0.316067669	0.373630265	0.955595653	0.0000161
100820742	GPX1	0.313426398	0.377855394	0.9555406	0.0000162
101120390	FLNA	0.466539622	0.174071641	0.955286912	0.0000166
101111635	INSYN1	0.435702577	0.208156716	0.955183308	0.0000167
101122546	PPP1R12C	0.320542226	0.36652607	0.954912513	0.0000171
101112333	PHOSPHO1	0.381056065	0.277295528	0.954617649	0.0000176
101107049	FES	0.309942573	0.383463993	0.954314391	0.000018
101107297	CENPX	0.468934726	0.171573621	0.954309856	0.000018
101107153	LOC101107153	0.502906413	0.138446674	0.954199326	0.0000182
105610424	WDR24	0.503030038	0.13833394	0.954159275	0.0000183
101114606	CHPF2	0.393206616	0.260965254	0.953952775	0.0000186
101110783	HSF4	0.445706184	0.196708097	0.953643763	0.0000191
101102700	TBC1D2	0.527986247	0.116721581	0.953632529	0.0000191
101113184	TCIRG1	0.436981707	0.206671864	0.953616249	0.0000191

101103783	RPL15	0.411193355	0.237791917	0.953607139	0.0000192
100302028	ZGPAT	0.363151904	0.302339999	0.95357956	0.0000192
105609445	TGFBR3L	0.403957935	0.246969417	0.953577621	0.0000192
101118712	LOC101118712	0.403957935	0.246969417	0.953577621	0.0000192
101108328	ANKEF1	0.403957935	0.246969417	0.953577621	0.0000192
101106883	LOC101106883	0.403957935	0.246969417	0.953577621	0.0000192
105610928	LOC105610928	0.403957935	0.246969417	0.953577621	0.0000192
101108299	ANAPC2	0.403957935	0.246969417	0.953577621	0.0000192
105616534	-	0.403957935	0.246969417	0.953577621	0.0000192
105609432	LOC105609432	0.403957935	0.246969417	0.953577621	0.0000192
101101833	-	0.403957935	0.246969417	0.953577621	0.0000192
101102508	ERF	0.403957935	0.246969417	0.953577621	0.0000192
101122951	MFSD10	0.403957935	0.246969417	0.953577621	0.0000192
105615790	-	0.403957935	0.246969417	0.953577621	0.0000192
100147800	SLC25A10	0.403957935	0.246969417	0.953577621	0.0000192
101111917	B3GAT3	0.389553049	0.265818595	0.953358286	0.0000196
101105095	MYBL2	0.415863793	0.231971428	0.953337776	0.0000196
101123008	MAPK3	0.355951801	0.312737367	0.953334955	0.0000196
101118784	TBC1D9B	0.275536977	0.440976239	0.953212466	0.0000198
101104725	MRPL23	0.472753253	0.167635444	0.95319495	0.0000198
101113157	MAF1	0.398008479	0.25466143	0.953150942	0.0000199
101120164	TRMT44	0.524818637	0.119339258	0.952926048	0.0000203
101119851	KLHL22	0.33055769	0.350871167	0.952814072	0.0000205
101121704	MZF1	0.621481145	0.05510263	0.952759958	0.0000206
100913171	ATG9A	0.363532303	0.301795846	0.952736645	0.0000206
101106321	MYH9	0.495761986	0.14505752	0.952642694	0.0000208
101120386	LOC101120386	0.46839726	0.172132311	0.952483091	0.0000211
100294595	SOCS3	0.329982116	0.351761532	0.952443298	0.0000211
101118647	LOC101118647	0.523657674	0.120307745	0.952431899	0.0000211
101107161	CARD19	0.305972906	0.389903906	0.952426917	0.0000212
101120369	TSPAN32	0.340134747	0.33622437	0.952024161	0.0000219
101122490	THAP4	0.468466043	0.172060752	0.951885311	0.0000221
101103192	FGD2	0.555693684	0.095352194	0.951869081	0.0000221

101113841	TBRG4	0.407047607	0.243026616	0.951865143	0.0000222
101108969	MAP3K11	0.36597609	0.298312475	0.951786466	0.0000223
554319	PPIB	0.529899354	0.115158137	0.951770634	0.0000223
101103267	CAMKK2	0.52804776	0.116671106	0.951749323	0.0000224
101115232	PIK3IP1	0.346630121	0.326472536	0.95155032	0.0000227
101117866	GMEB2	0.466516543	0.174095816	0.951328285	0.0000231
101113949	TIMM17A	0.565182014	0.088654119	0.951063615	0.0000236
101120095	CISD3	0.435084804	0.208876044	0.951060416	0.0000237
101108482	FKBP8	0.483439631	0.156903938	0.95074889	0.0000243
101106280	-	0.27101818	0.448807724	0.950672487	0.0000244
101111266	LOC101111266	0.63901162	0.046701662	0.950669002	0.0000244
101120406	PICK1	0.53468731	0.111302939	0.950538521	0.0000247
101101871	-	0.546230534	0.102344923	0.950514093	0.0000247
101115548	EXOSC4	0.28543631	0.424041125	0.950329625	0.0000251
101123544	PDXP	0.265086008	0.459183267	0.950297452	0.0000251
101102766	SAYSD1	0.589393316	0.072953056	0.950228074	0.0000253
101105738	PSD4	0.406815051	0.243322151	0.950195287	0.0000253
101109888	MROH1	0.463226162	0.177562925	0.950124195	0.0000255
101107515	HDAC7	0.474320179	0.166035209	0.950008024	0.0000257
101123589	NBEAL2	0.38850346	0.267221938	0.949859473	0.000026
101108831	LOC101108831	0.559086475	0.092921321	0.949840815	0.0000261
101108956	CSK	0.372867682	0.288605276	0.949753613	0.0000262
101105700	BET1L	0.413240108	0.235231155	0.94968163	0.0000264
677670	BGLAP	0.450276928	0.191602221	0.94966895	0.0000264
101103562	RAB11B	0.566926202	0.087456638	0.949506526	0.0000268
105615315	-	0.549630771	0.099796167	0.949273936	0.0000272
101110022	LOC101110022	0.483220845	0.157119375	0.949271135	0.0000272
101104859	TBC1D9	0.392407117	0.262023093	0.949217128	0.0000274
101122026	IZUMO4	0.452836868	0.188776855	0.949167134	0.0000275
105601911	-	0.291490277	0.413836585	0.949093787	0.0000276
101107345	RPS19BP1	0.285152405	0.424522529	0.949089157	0.0000276
101106403	TFE3	0.401650196	0.2499375	0.949061602	0.0000277
101106780	LRRC14	0.415003449	0.233037508	0.94876201	0.0000283

101110857	FLII	0.3882882	0.26751025	0.94865068	0.0000286
101111852	MAST2	0.40399627	0.246920279	0.948641046	0.0000286
101102716	PNPLA7	0.496895779	0.143995814	0.948527176	0.0000289
101111336	SPSB3	0.411214229	0.237765722	0.948448681	0.000029
105609279	-	0.573648794	0.082938462	0.948388678	0.0000292
101113539	RPL29	0.328727836	0.353705743	0.948228051	0.0000295
105604794	LOC105604794	0.450096485	0.191802303	0.947967597	0.0000301
443331	TIMP1	0.405572216	0.244904971	0.94793613	0.0000302
101120430	OGFR	0.439293981	0.204003296	0.947903602	0.0000303
100302312	ARL4C	0.52595992	0.118391939	0.947879565	0.0000303
101117955	LOC101117955	0.361812543	0.30426006	0.947872463	0.0000303
101121783	LOC101121783	0.449712338	0.192228664	0.94766626	0.0000308
101112277	CASTOR2	0.56268531	0.090386441	0.947644466	0.0000309
101105775	TIMM10	0.345450688	0.328232274	0.947521322	0.0000311
443269	AGRP	0.259182998	0.469612857	0.947319582	0.0000316
101109965	LONP1	0.507135047	0.134622501	0.947302812	0.0000317
443133	IGFBP5	0.545674133	0.102765871	0.947162671	0.000032
101101791	-	0.545674133	0.102765871	0.947162671	0.000032
101118379	DMPK	0.545674133	0.102765871	0.947162671	0.000032
443278	UCP3	0.545674133	0.102765871	0.947162671	0.000032
101103099	VWA7	0.545674133	0.102765871	0.947162671	0.000032
443033	SFTPA1	0.545674133	0.102765871	0.947162671	0.000032
101114289	SYNGR2	0.35991048	0.306997846	0.947111812	0.0000321
101107924	LMNA	0.501670861	0.139576476	0.947087241	0.0000322
101106225	CARHSP1	0.326325446	0.357444663	0.947031003	0.0000323
101118800	NDUFAF8	0.390683997	0.264311008	0.946678074	0.0000332
101123065	MEGF8	0.340195502	0.336132471	0.946611645	0.0000333
101116402	REC8	0.256923751	0.473631936	0.946551864	0.0000335
101121935	LYL1	0.374115495	0.28686605	0.946375937	0.0000339
101111045	YIF1B	0.490710907	0.149845311	0.946259418	0.0000342
101119168	LEMD2	0.400611331	0.251280077	0.9462037	0.0000343
101101881	TST	0.493444026	0.147242914	0.946123347	0.0000345
101122229	SLC22A23	0.5159286	0.12688006	0.945902986	0.0000351

101120968	ZNHIT2	0.358871281	0.308499117	0.945851191	0.0000352
101102753	GSK3A	0.396897666	0.256112115	0.945821525	0.0000353
101110811	MRPS34	0.383574815	0.27386584	0.945751855	0.0000355
101121405	IRAK1	0.523621762	0.120337781	0.945570418	0.0000359
101105734	-	0.417947998	0.229400278	0.945552608	0.000036
101110240	GCHFR	0.275548095	0.440957048	0.945524226	0.0000361
101111056	NT5DC2	0.313942337	0.377028231	0.945455028	0.0000362
101115077	TMEM223	0.332048091	0.348570944	0.945453462	0.0000363
101115739	EHMT2	0.602425299	0.065306707	0.945147452	0.0000371
101107982	SPATA2L	0.431539062	0.213032368	0.945125705	0.0000371
101109129	LOC101109129	0.41326421	0.235201094	0.944927247	0.0000376
101119072	NAPA	0.517747134	0.125314153	0.944681965	0.0000383
105607128	LOC105607128	0.465323035	0.175348736	0.944672775	0.0000383
105609580	LOC105609580	0.461883571	0.17898929	0.694368542	0.025878627
101117217	GPAT4	0.525807638	0.118518068	0.944522699	0.0000387
101107487	RRNAD1	0.413253859	0.235214004	0.944458615	0.0000389
101117734	NDUFB11	0.344016606	0.330378544	0.944391773	0.0000391
101114848	ZNF81	-0.461022187	0.179907983	-0.576075825	0.081345004
101110967	CAVIN3	0.504461993	0.137032234	0.944283666	0.0000394
101108850	STMN3	0.456126023	0.185182792	0.944052362	0.0000401
101116753	ZNF335	0.454548875	0.186901074	0.943991438	0.0000402
100302082	MAP2K7	0.49242991	0.148205292	0.943591405	0.0000414
101122243	ATG4B	0.416651691	0.230997541	0.943516744	0.0000416
101111541	LOC101111541	0.269166159	0.452035502	0.943436032	0.0000418
101102529	LOC101102529	0.337425355	0.340335725	0.94341605	0.0000419
780477	CLDN4	0.336973211	0.341024317	0.943386002	0.000042
101116532	DTX2	0.385885632	0.27073972	0.943341174	0.0000421
101107422	CD99L2	0.49353883	0.147153142	0.943334266	0.0000421
101111024	PPP1R14D	0.448065244	0.194063044	0.943281214	0.0000423
101114060	SEC61A1	0.385789135	0.270869871	0.943252372	0.0000424
101102925	BICRA	0.363913236	0.301251452	0.943217623	0.0000425
101121742	ARID5B	-0.459308476	0.181743989	-0.755125465	0.011563498
101102864	GNB2	0.350087033	0.321343002	0.943153206	0.0000426

105607221	DNLZ	0.518849932	0.12437042	0.943115822	0.0000428
101121721	ZNF76	0.456151441	0.185155176	0.943077716	0.0000429
101121166	MAFF	0.295378374	0.407344639	0.943062719	0.0000429
443433	TPSB2	0.458174977	0.182964433	0.943056798	0.0000429
101115161	ITPK1	0.340000074	0.336428125	0.943056606	0.0000429
101121534	MYL9	0.423746079	0.222332977	0.942991264	0.0000431
101105542	S100A1	0.347141724	0.325710737	0.942990807	0.0000431
101109035	LOC101109035	0.435962333	0.207854686	0.942595014	0.0000443
101104031	ROGDI	0.409928556	0.239382154	0.942186167	0.0000456
101120279	EXOSC7	0.439413781	0.203865583	0.942027136	0.0000461
101120442	KREMEN1	0.458484098	0.182631123	0.623336099	0.054169743
105610305	ZNF783	0.458484098	0.182631123	0.623336099	0.054169743
105611242	LOC105611242	0.458484098	0.182631123	0.623336099	0.054169743
101112860	CSKMT	0.408486491	0.241202535	0.941987131	0.0000462
101108230	SLC49A3	0.362709083	0.302974099	0.941904826	0.0000464
101112294	PRICKLE3	0.526934741	0.117586517	0.941832725	0.0000467
101119803	PSMG4	0.349442846	0.322295678	0.941804751	0.0000468
101118488	SSH3	0.38342396	0.2740706	0.941541853	0.0000476
105614324	-	0.388154318	0.267689653	0.941240059	0.0000486
101106154	MFSD5	0.328430342	0.35416767	0.941159784	0.0000488
101107364	MTDH	-0.457815943	0.183352013	-0.708500787	0.021824981
101111954	C3H12orf73	0.632094093	0.049906538	0.941139709	0.0000489
101117230	LRRC8A	0.423803139	0.222264051	0.941118892	0.000049
101109235	TMEM53	0.429445447	0.215508652	0.941022912	0.0000493
101109581	MRPL28	0.444255872	0.19834461	0.941003295	0.0000493
101102252	NAGLU	0.473382863	0.166991346	0.940965827	0.0000495
101114059	ASB2	0.396126996	0.257121266	0.940946535	0.0000495
105608253	LETM1	0.519153803	0.124111155	0.940905547	0.0000497
101122801	TNFSF12	0.425657398	0.220030834	0.940793891	0.00005
101106587	SLC25A42	0.349523172	0.322176805	0.940724656	0.0000503
106991663	-	0.290907173	0.414814378	0.940531197	0.0000509
101106286	UNC45A	0.466440585	0.174175396	0.940457801	0.0000512
101107370	TEN1	0.422064673	0.224369503	0.94044669	0.0000512

101111544	REEP2	0.347310423	0.325459738	0.940409424	0.0000513
101110684	OPLAH	0.283761197	0.426885216	0.940401066	0.0000513
105604575	-	0.455098313	0.186301408	0.685653875	0.028619867
101120718	CD2BP2	0.234854218	0.513667255	0.940307488	0.0000517
101106452	LOC101106452	0.262535113	0.463677491	0.940274429	0.0000518
101121449	UBE2M	0.291336585	0.414094202	0.940240691	0.0000519
101104191	SIGIRR	0.484085212	0.156269279	0.940167707	0.0000521
101108441	BNIP3	0.66130748	0.037314368	0.940089992	0.0000524
101108500	CAMTA2	0.445302893	0.197162372	0.939982927	0.0000528
101119652	GRTP1	0.41124714	0.237724425	0.939952068	0.0000529
101123165	BOLA1	0.556337044	0.094888163	0.939922059	0.000053
101108401	FLOT2	0.290383117	0.415694082	0.939735122	0.0000536
101104826	MYO1F	0.355227266	0.313793891	0.939711964	0.0000537
101115893	PREX1	0.221889193	0.537817428	0.939686273	0.0000538
101120596	SBSN	0.566115123	0.08801219	0.939538374	0.0000543
101109666	SNN	0.325577503	0.358612745	0.939529024	0.0000544
101117780	SLC35C2	0.452128251	0.18955648	0.939394952	0.0000548
101119385	WASHC1	0.417948865	0.229399212	0.939311153	0.0000551
101122271	LRRC61	0.54830933	0.100781859	0.939122172	0.0000558
101103925	SFI1	0.382395633	0.275468599	0.939088028	0.0000559
105615257	LOC105615257	0.477784095	0.162530234	0.938906219	0.0000566
101122350	WDR91	0.543231989	0.10462643	0.938830432	0.0000569
101120162	C5H19orf57	0.4363536	0.207400221	0.938766457	0.0000571
101115754	HSPB1	0.298953819	0.401417815	0.938236966	0.0000591
101123368	LOC101123368	0.530326124	0.114811166	0.938181749	0.0000593
101102126	C1QA	0.464616448	0.176093013	0.938149414	0.0000594
101101972	NOL12	0.462167742	0.178686823	0.938114515	0.0000595
101103607	SIKE1	-0.451719871	0.19000664	-0.782584973	0.00744891
101120780	DHX37	0.473964363	0.166397783	0.938051014	0.0000598
101103287	ANKS3	0.476342944	0.163983021	0.93801044	0.0000599
101123244	LOC101123244	0.451451424	0.190302892	0.590922571	0.072027082
106991551	C14H16orf74	0.509970351	0.132095179	0.937971282	0.0000601
101111026	LOC101111026	0.191525334	0.596071373	0.937908972	0.0000603

101102725	C5H19orf53	0.312007732	0.380134414	0.93776547	0.0000609
101120284	ADAMTS15	0.538226519	0.108505975	0.937585634	0.0000615
101108634	MICOS10	0.427589998	0.217716972	0.937576639	0.0000616
101122584	SBF1	0.595687382	0.069191113	0.937573258	0.0000616
100913152	HSPA1A	0.353900215	0.315733857	0.937541908	0.0000617
101110561	ARHGEF11	0.561615903	0.091135015	0.937536574	0.0000617
101108782	DALRD3	0.541348701	0.106075648	0.93748708	0.0000619
101121898	AP2A2	0.352217366	0.318202951	0.937468113	0.000062
101102990	LOC101102990	0.417507437	0.229942417	0.937299381	0.0000627
101121994	DTYMK	0.242978584	0.498768333	0.937290364	0.0000627
101113519	TPPP3	0.422912621	0.223341142	0.937225958	0.000063
101103845	CINP	0.449831259	0.192096615	0.690721895	0.02700275
101115302	PPP2R1A	0.459300259	0.181752819	0.937202293	0.000063
101103370	SMURF1	0.449720704	0.192219373	0.670847061	0.033720686
106990907	-	0.580881097	0.078248688	0.937182143	0.0000631
101123204	MGAT3	0.55138782	0.098495047	0.937144976	0.0000633
101122540	DDRGK1	0.41251794	0.236132901	0.937039214	0.0000637
105608233	LOC105608233	0.34670821	0.326356198	0.936873231	0.0000643
105603814	LOC105603814	0.34670821	0.326356198	0.936873231	0.0000643
101114224	MRPL11	0.33609436	0.342364795	0.936866166	0.0000644
101110306	CTDSP1	0.264586968	0.46006095	0.936419313	0.0000662
101113342	SLC46A3	-0.448446167	0.193637901	-0.667822267	0.034833555
101102467	TARBP2	0.433837548	0.210332701	0.936308942	0.0000666
101115049	TMC6	0.312892521	0.378712253	0.936241415	0.0000669
101116070	VMO1	0.447433853	0.194768931	0.936076852	0.0000676
101121173	LRPAP1	0.336284626	0.342074361	0.936016206	0.0000678
101102584	USF2	0.485912507	0.154481311	0.935944443	0.0000681
101110774	GGNBP2	-0.447621843	0.194558604	-0.903579147	0.000336128
101108998	LOC101108998	0.301564406	0.397116639	0.935786501	0.0000688
101110813	CARD11	0.465363133	0.175306556	0.935774446	0.0000689
101108390	LOC101108390	0.491288938	0.149292615	0.935680574	0.0000693
101109216	FAH	0.50943607	0.13256917	0.935571234	0.0000697
101104767	OGFOD3	0.379391772	0.279574457	0.935533665	0.0000699

101102082	B9D2	0.241984621	0.500581207	0.935516739	0.0000699
101112577	FAM92A	-0.446114233	0.196249084	-0.735385443	0.015361608
101105444	-	0.636349529	0.047918228	0.935489926	0.0000701
101112566	RPL18A	0.432730389	0.211630631	0.935467273	0.0000702
101115149	SAMD4B	0.323002085	0.362649439	0.935377396	0.0000705
101117299	LOC101117299	0.445561968	0.196870474	0.682564684	0.029637331
100415775	ID3	0.556962169	0.094438663	0.935375334	0.0000705
101104587	RHBDF2	0.268118038	0.453866831	0.935201184	0.0000713
101119816	SGTA	0.390884439	0.264044304	0.935042823	0.000072
101108562	TPRN	0.469072558	0.171430521	0.934617695	0.0000738
105602361	LOC105602361	0.395486141	0.257962102	0.93432433	0.0000752
101110295	FOXK1	0.341051657	0.334838808	0.934106816	0.0000761
101103020	KCNK5	0.36592892	0.298379508	0.934008557	0.0000766
101117430	SP2	-0.444037141	0.198592109	-0.627651986	0.05203986
101108334	TSKS	0.292023221	0.412943856	0.933677815	0.0000781
101111302	JPT1	0.613291847	0.059348183	0.933572295	0.0000786
101114736	MRPL21	0.332374221	0.34806863	0.933344424	0.0000796
101106640	NLRP6	0.308670991	0.385521172	0.933300507	0.0000799
101121127	CCDC86	0.323488134	0.361885879	0.933280293	0.0000799
101107446	MAEA	0.47972921	0.160581712	0.933248208	0.0000801
101107535	KIF1C	0.33718919	0.340695304	0.933247887	0.0000801
101119926	FFAR2	0.442216785	0.200658852	0.68687925	0.028222969
100145870	SLC66A2	0.422851461	0.223415225	0.93323727	0.0000801
101119246	NUMA1	0.424628396	0.221268545	0.93322399	0.0000802
101111097	MED16	0.43880714	0.204563485	0.933200127	0.0000803
101114732	AARS2	0.452974748	0.188625377	0.933080931	0.0000809
101106029	ZNF575	0.37567926	0.284694428	0.93290003	0.0000817
101120515	ITPKC	-0.441124189	0.201905309	-0.47619121	0.164136431
101114573	HOGA1	0.37567926	0.284694428	0.93290003	0.0000817
101110218	PLEK	-0.441013606	0.202031715	-0.418128161	0.229178784
101117514	PYCR3	0.311250681	0.381353329	0.932862239	0.0000819
101122656	AXIN1	0.42587164	0.219773638	0.932760778	0.0000824
101107752	POLR3K	0.370333468	0.292154892	0.932512825	0.0000836

101118854	AKNA	0.459474595	0.181565533	0.932459344	0.0000839
101110113	CLIP2	0.420105123	0.226756264	0.932347704	0.0000844
101102662	NPEPL1	0.470074104	0.170392828	0.932269463	0.0000848
101120611	POMGNT2	0.500789047	0.140386257	0.932225895	0.000085
101114351	AP1M2	0.303209023	0.394418423	0.932162957	0.0000853
443351	CENPB	0.223715034	0.534389166	0.932115641	0.0000856
101109701	LOC101109701	0.359923903	0.306978479	0.931730562	0.0000875
100135695	PSAP	0.409415636	0.240028749	0.931688419	0.0000877
101103257	NDUFA3	0.487084862	0.153340731	0.931341144	0.0000894
101112660	RAI1	0.546701466	0.101989491	0.931133712	0.0000905
101112687	ALDH3B1	0.293790317	0.409990339	0.931129125	0.0000905
101107279	DOK3	0.272885533	0.445563832	0.930976394	0.0000913
101108778	LOC101108778	0.416342036	0.231380015	0.930972235	0.0000913
101112840	SNX21	0.420209649	0.226628587	0.930893803	0.0000917
105610921	PADI4	0.29021913	0.415969537	0.93081381	0.0000922
443046	ATP7B	0.468787273	0.171726789	0.930589328	0.0000933
101105916	IDUA	0.395978568	0.257315877	0.930512065	0.0000937
101103319	JMJD4	0.326262115	0.357543495	0.930246809	0.0000952
101111103	ISM2	0.295728935	0.406761704	0.930092185	0.000096
105604861	-	0.295728935	0.406761704	0.930092185	0.000096
101111100	CXCL13	0.437935737	0.205568397	0.317734706	0.370975639
101103471	IER2	0.28902212	0.41798281	0.930026897	0.0000963
105610709	LOC105610709	0.381310787	0.276947628	0.929921713	0.0000969
101120848	SPEM1	0.4378224	0.205699307	0.520672446	0.122820471
101104353	NFKBIL1	0.629652932	0.051071557	0.929527118	0.0000991
101114192	FAM174C	0.294787867	0.408327472	0.929512429	0.0000991
101107019	GRK6	0.40787989	0.241970588	0.929494215	0.0000992
105610931	-	0.337637187	0.34001336	0.929116905	0.000101326
100036760	RPL13A	0.407868078	0.241985557	0.928999398	0.000101985
101102937	PPP1R13B	0.427493278	0.217832441	0.928909354	0.000102492
101106776	LOC101106776	-0.43676334	0.206924915	-0.76167109	0.010465506
101108954	PEBP1	0.543677032	0.1042858	0.928702847	0.000103661
101105150	BLOC1S1	0.364964961	0.299751131	0.928702715	0.000103662

101102156	LOC101102156	0.258646623	0.470565674	0.928623624	0.000104113
101105076	ADAMTS10	0.54878974	0.100422801	0.928548541	0.000104542
105602913	LOC105602913	0.436260163	0.207508698	0.538367197	0.108395724
101113713	FMO5	-0.43615946	0.207625648	-0.49737645	0.14354714
106991831	LOC106991831	0.478420379	0.16189128	0.92853618	0.000104613
101105195	ZMYND12	0.435899672	0.207927522	0.604562661	0.064105056
101118905	SLC25A29	0.459861593	0.181150196	0.928534186	0.000104624
443104	-	0.358766154	0.308651203	0.92852999	0.000104648
101111605	BCAP31	0.320292499	0.366920777	0.928423974	0.000105257
101106244	TP53I3	0.435667552	0.20819746	0.654374831	0.040082445
100126570	ASZ1	0.509901202	0.132156466	0.928228952	0.000106383
101118138	EVL	0.609896659	0.061169723	0.928211799	0.000106482
101117378	CSTF2T	0.353770841	0.315923319	0.928124185	0.000106992
101101924	UBE2Q2	-0.435294102	0.208632179	-0.790850094	0.006448132
106991980	LOC106991980	0.44390508	0.198741625	0.92800306	0.000107699
101110393	AZIN2	0.575220228	0.08190447	0.927858216	0.000108548
101113227	SURF2	0.440031	0.203156931	0.927789986	0.00010895
105602976	LOC105602976	0.369697811	0.293048891	0.927759762	0.000109129
106991403	-	0.434657578	0.209374339	0.591743106	0.071533411
101118845	CHST12	0.365802867	0.298558678	0.927714529	0.000109396
101106548	HDDC3	0.417434085	0.230032751	0.927594126	0.000110111
100187549	HPCAL1	0.52316529	0.120719971	0.927503299	0.000110652
105602949	LOC105602949	0.523653536	0.120311206	0.927458606	0.000110919
101111567	AGTRAP	0.247030537	0.491406993	0.927430455	0.000111087
101122492	ZNF768	0.4542674	0.187208719	0.927391712	0.000111319
101115137	HOXB7	-0.433263069	0.211005593	-0.666584706	0.035295955
101108327	SAMD11	0.393934925	0.260003661	0.927202565	0.000112458
100147781	SLC39A3	0.295105146	0.407799258	0.927113736	0.000112995
106990325	LOC106990325	0.542685925	0.10504534	0.92699568	0.000113713
101120041	CUX1	0.49393783	0.146775683	0.926894624	0.000114329
101118855	UBL4A	0.292435319	0.412254181	0.926817565	0.000114801
101109247	EMD	0.458949507	0.182129971	0.926750527	0.000115213
101120043	LOC101120043	0.54637277	0.102237489	0.926686223	0.000115609

101116615	ESRRA	0.293368941	0.410693713	0.92657666	0.000116286
101110689	TBC1D16	0.315213354	0.374994317	0.926446112	0.000117096
101102013	ORAI1	0.455857026	0.185475201	0.926339959	0.000117758
101117651	STPG1	0.643686564	0.044615489	0.926293477	0.000118049
100302637	NR6A1	0.483823125	0.156526745	0.926086385	0.000119351
101111455	ATP5ME	0.401304988	0.250383186	0.926021323	0.000119762
101102042	LOC101102042	0.349265461	0.322558267	0.925961866	0.000120139
101114550	TRAPPC6A	0.483770767	0.156578211	0.925900596	0.000120528
101113302	FAM25A	0.627600662	0.052064856	0.925844133	0.000120887
101107545	RRAS	0.408823024	0.240777024	0.925792232	0.000121218
101104511	DPH1	0.491111187	0.149462444	0.92547582	0.000123251
101106414	RARG	0.267100655	0.455647641	0.92526884	0.000124594
101122545	LOC101122545	0.440296204	0.202852879	0.92511977	0.000125568
101102524	PAQR6	0.419150381	0.227924357	0.925032604	0.00012614
101117276	MADD	0.459601854	0.181428893	0.924654916	0.000128641
101104259	DVL1	0.465422209	0.175244422	0.924329069	0.000130828
101106768	FBXW9	0.465422209	0.175244422	0.924329069	0.000130828
101102089	ADCY2	0.465422209	0.175244422	0.924329069	0.000130828
101117268	NECAB3	0.250626234	0.484913837	0.924188172	0.000131783
101112553	ACRBP	0.494759379	0.146000344	0.924183391	0.000131815
101111981	SPNS3	0.238548999	0.506868751	0.923929125	0.00013355
101122645	LOC101122645	0.36182969	0.304235438	0.923785645	0.000134537
101112813	FDX2	0.525131359	0.119079216	0.92369466	0.000135165
101118996	MAPKAPK3	0.307471745	0.387466238	0.923583135	0.000135939
101102302	NECAP1	-0.428759622	0.216323405	-0.745424309	0.013335948
101101816	-	0.268546169	0.453118377	0.923548859	0.000136177
101104265	ARHGEF1	0.374691075	0.2860657	0.923536325	0.000136264
101103661	TMEM9	0.564061919	0.089428636	0.923460955	0.000136789
101121366	SCAND1	0.504501122	0.13699677	0.923302939	0.000137896
101118814	PCSK7	0.399704944	0.252454713	0.923144088	0.000139014
101119235	TPRG1L	0.306527308	0.389001378	0.92313344	0.00013909
101109052	TBC1D10C	0.473376295	0.166998058	0.923041483	0.00013974
101118990	LOC101118990	0.354390388	0.315016563	0.922953897	0.000140363

101117564	-	0.428126032	0.217077669	0.584385626	0.076039291
101111644	GID4	0.427771356	0.217500555	0.662636979	0.036798706
101107351	FCHSD1	0.359850054	0.307085035	0.922934812	0.000140498
105602053	LOC105602053	0.4050451	0.245578016	0.922882955	0.000140868
101122200	TNFRSF13B	0.400615975	0.251274066	0.922851136	0.000141095
101104415	UBE2T	0.584709517	0.075837162	0.922601144	0.000142889
105610092	LOC105610092	0.264676246	0.459903878	0.922516331	0.000143501
101115917	ACTN3	0.453359098	0.1882035	0.92249489	0.000143656
101112670	BCL2L12	0.399671105	0.252498625	0.922366743	0.000144586
101109560	LIN7B	0.278896012	0.435195544	0.922225853	0.000145613
101104386	GALNT14	0.513877687	0.128660534	0.92215673	0.000146119
100302083	CDKN1A	0.31606426	0.373635704	0.92206019	0.000146827
101115537	PIP5K1C	0.389728055	0.265584999	0.92202215	0.000147107
101110170	CEACAM20	0.37581163	0.284511012	0.921931515	0.000147776
101115252	LOC101115252	0.262204865	0.464260753	0.921637691	0.000149959
101105086	GSDMB	0.365962973	0.298331115	0.921541421	0.000150679
101119130	LOC101119130	0.320204145	0.367060476	0.921351328	0.000152109
101103911	ADIPOR1	0.442411318	0.200437395	0.921234034	0.000152996
101112923	PDF	0.399520232	0.252694465	0.921218099	0.000153117
101120112	ESS2	0.379601723	0.279286412	0.921197117	0.000153276
443504	EEF1D	0.433723586	0.210466087	0.921192216	0.000153313
101106095	STUM	0.436261925	0.207506652	0.921122479	0.000153843
105606095	-	0.304535047	0.392249363	0.920919862	0.000155391
101113149	GPX4	0.220891431	0.539694574	0.920854675	0.000155892
101105128	ADAM15	0.240635227	0.503046794	0.920826457	0.000156109
101107909	SMIM12	0.468979063	0.171527582	0.920388285	0.000159507
101110250	ZNHIT3	0.457837477	0.183328754	0.920135074	0.000161496
101115985	MYH14	0.328128999	0.354635883	0.920022186	0.000162388
101119527	PAQR4	0.272011449	0.447080928	0.919688302	0.000165048
101102671	LOC101102671	0.314064707	0.376832176	0.919655507	0.000165312
101114909	LOC101114909	0.327462296	0.355672881	0.919544709	0.000166203
101104680	LRFN3	0.360697569	0.305863356	0.919543597	0.000166211
101119242	AKT2	0.376364614	0.28374547	0.919514153	0.000166449

101117837	BID	0.338218882	0.339128942	0.919422698	0.000167188
443151	EP4C	0.361574695	0.304601704	0.919265543	0.000168463
101104340	RELT	0.231702974	0.519495336	0.918987117	0.00017074
101121963	STRN4	0.395070507	0.258508249	0.918849789	0.000171872
101110513	C7H15orf62	0.443393873	0.199321021	0.918345864	0.000176071
101110439	C14H19orf33	0.313835071	0.377200127	0.918190854	0.000177378
101106256	BORCS8	0.279380239	0.43436511	0.917980395	0.000179164
101105073	FARSA	0.327921869	0.354957892	0.917949801	0.000179424
101103166	NAT9	0.394650332	0.259061008	0.917939418	0.000179513
101116668	TNFRSF25	0.416890377	0.230702969	0.917919709	0.000179681
101108595	AP2A1	0.364448791	0.300486968	0.917809986	0.000180619
101115319	KLF15	0.394159698	0.259707286	0.91777182	0.000180946
101117799	LOC101117799	0.54878272	0.100428041	0.917610974	0.00018233
101119329	SELENOW	0.22086498	0.539744372	0.917561758	0.000182755
101108532	PGAM1	0.239397954	0.505312003	0.91743308	0.000183869
101112180	LZTS2	0.356213733	0.312355875	0.917320521	0.000184848
105614838	LOC105614838	0.42281929	0.223454199	0.612744214	0.059639541
101112442	DGKD	0.247657254	0.490272588	0.917138077	0.000186443
105615848	LOC105615848	0.422501112	0.223839869	0.597405991	0.06818638
101116814	PHYHD1	0.406697228	0.24347196	0.916913374	0.000188421
101113536	TMED3	0.396810337	0.256226358	0.916825913	0.000189195
101104618	RNH1	0.427214785	0.218165113	0.916719676	0.000190139
101113669	ANKRD39	0.629582188	0.051105585	0.916606304	0.00019115
101103609	NPM3	0.421373402	0.225209841	0.618808874	0.056465158
101120127	FAM131A	0.500618191	0.140543488	0.916569311	0.00019148
101102145	RPS14	0.343541864	0.331090642	0.916276456	0.000194113
101104086	KMT2B	0.400991979	0.250787682	0.916144865	0.000195304
101102320	SEC14L1	0.313521742	0.377702469	0.916078918	0.000195903
101113275	LOC101113275	0.373753716	0.287369723	0.916020154	0.000196438
101121436	DRC3	0.373753716	0.287369723	0.916020154	0.000196438
101102006	PAFAH1B3	0.326521462	0.357138857	0.915995871	0.000196659
100192428	DBP	0.362146916	0.303780112	0.915875342	0.000197761
101106772	SRD5A3	0.419895252	0.227012742	0.606717405	0.062908404

101103118	GP5	0.49598275	0.144850419	0.915792822	0.000198518
101120054	XPNPEP2	0.3106546	0.382314414	0.915739152	0.000199011
101107546	RNF166	0.328964615	0.353338308	0.915649023	0.000199842
101102844	RPUSD3	0.462044352	0.17881812	0.914856867	0.000207252
101108740	KIAA0930	0.414698245	0.233416361	0.914701336	0.00020873
101115063	ATG13	0.436837337	0.206839145	0.914669537	0.000209033
101104032	ZNF646	0.426276961	0.21928752	0.914340528	0.000212189
101119725	TLE6	0.417973562	0.229368842	0.602315323	0.065368933
100302341	GNAI2	0.274485561	0.442792835	0.914073141	0.00021478
101116260	AEN	0.359125951	0.308130852	0.913939291	0.000216086
443451	ATOX1	0.390780204	0.264182978	0.913868168	0.000216782
101116579	LOC101116579	0.571982047	0.084044318	0.913851567	0.000216944
105602374	-	0.532659971	0.112925286	0.913779649	0.00021765
101123668	UBA7	0.251546316	0.483258327	0.913762279	0.000217821
101103065	SNAPC2	0.402562526	0.248761751	0.913600634	0.000219415
101117118	RGL2	0.42815359	0.217044831	0.913333194	0.000222071
101114881	RPH3AL	0.303467512	0.393995143	0.913140813	0.000223996
101106614	PLEKHF1	0.459742477	0.181277974	0.913135784	0.000224046
101119152	POLR2I	0.442504306	0.200331587	0.912899725	0.000226425
101119435	MVP	0.256231427	0.474866553	0.91288397	0.000226585
105615565	-	0.465140789	0.175540524	0.91274171	0.000228028
101105273	TTYH1	0.285499878	0.42393337	0.912739386	0.000228052
101102301	NAV3	0.426132561	0.219460635	0.912619652	0.000229272
100174905	PSMB8	0.432117623	0.212350957	0.912606494	0.000229406
101118484	LOC101118484	0.386408684	0.270034842	0.912537652	0.00023011
101109967	SSBP4	0.525724695	0.118586802	0.91239863	0.000231537
101109238	WIPI2	0.364838901	0.29993075	0.912393908	0.000231586
101122538	KCNAB2	0.567785232	0.086870699	0.91227655	0.000232795
101122654	TFAP4	0.371386041	0.290677753	0.912208903	0.000233495
101123327	HIP1R	0.317658874	0.371096193	0.912189826	0.000233692
101120763	LOC101120763	0.415284296	0.232689198	0.336837086	0.341231767
106991221	-	0.500481284	0.140669555	0.9120562	0.000235079
101118183	NOM1	0.385130348	0.27175933	0.911991763	0.00023575

101111735	FBXL8	0.425524691	0.220190235	0.911874466	0.000236975
101113285	LSM10	0.448133972	0.193986298	0.91183773	0.000237359
101103078	LIMD2	0.325833485	0.358212756	0.911825172	0.000237491
101103013	BCL7A	0.583027381	0.076890721	0.911643832	0.000239397
105604615	PRSS33	0.513257362	0.129202086	0.911582318	0.000240046
105614865	-	0.38710327	0.269100353	0.911399801	0.000241979
101118896	FAM160A2	0.163450459	0.651852243	0.911015957	0.000246083
101115644	GFOD2	0.377747894	0.281835355	0.910912883	0.000247193
101112608	RAB3B	0.227724228	0.526892437	0.910629833	0.000250262
101110707	TMEM116	0.413375804	0.235061933	0.493898245	0.146813104
105613076	-	0.513481938	0.129005865	0.91034896	0.000253335
101123515	LOC101123515	0.413771404	0.234568987	0.910222303	0.000254729
101118187	NFIC	0.413771404	0.234568987	0.910222303	0.000254729
101109487	SERF2	0.311349251	0.381194515	0.910064125	0.000256479
105608016	-	0.534526673	0.11143095	0.910061408	0.000256509
101103171	MEAK7	0.311888031	0.380327015	0.9098388	0.000258986
101117742	WBP1	0.343491365	0.331166435	0.909732739	0.000260173
106991679	LOC106991679	0.412634764	0.235986895	0.410351113	0.238850208
101102096	LOC101102096	0.366897693	0.297004407	0.909335516	0.000264652
101116048	TBXA2R	0.527154847	0.117405135	0.909257152	0.000265543
101115380	CRK	-0.41200711	0.23677193	-0.663353257	0.036522894
101120592	HOXB6	0.435615569	0.20825794	0.909180866	0.000266412
101108744	LRRC25	0.263316496	0.462298761	0.908924079	0.000269352
101115914	IFRD2	0.429838812	0.215042134	0.908889523	0.000269749
101120820	CDPF1	0.262628709	0.463512248	0.908879634	0.000269863
101118977	BCAS4	0.353591241	0.316186433	0.908811659	0.000270646
105606169	C22H10orf143	0.46809357	0.172448472	0.908798378	0.000270799
101113874	PCIF1	0.614217245	0.058857976	0.908702296	0.00027191
101113876	GPR4	0.424509668	0.22141161	0.90866241	0.000272372
101106458	COMMD9	0.300804676	0.398366068	0.908366639	0.000275816
101102211	-	0.497750533	0.143198549	0.908284346	0.00027678
101113618	NFKBIB	0.26011487	0.467959502	0.90813629	0.00027852
105603705	-	0.31412083	0.376742275	0.908133827	0.000278549

101111783	TLE4	-0.409140053	0.240376557	-0.501820117	0.139439696
101123416	IL17RC	0.206304313	0.567431379	0.907807766	0.00028241
101113226	CENPM	0.502746252	0.13859281	0.907712405	0.000283547
101103168	ZNF281	-0.408652203	0.240992956	-0.633249081	0.049361552
101113845	KEAP1	0.503030953	0.138333106	0.907637001	0.000284448
101115470	GRN	0.233119734	0.516871741	0.907580995	0.000285119
101119104	RORC	0.40821759	0.241542838	0.691020421	0.026909493
101120707	MMACHC	0.516286263	0.126571131	0.907533467	0.000285689
101106408	MUL1	0.390311949	0.264806438	0.907487365	0.000286243
100170327	FASN	0.518782834	0.124427713	0.907296919	0.000288539
101105264	CLUH	0.512030139	0.130277613	0.907227529	0.000289379
101111070	GNA12	0.310782869	0.382107502	0.907121556	0.000290665
101123498	RNF215	0.407703634	0.242194013	0.69052483	0.027064432
101112025	TMEM8B	0.407684835	0.242217849	0.557453017	0.09408667
101115366	ABHD8	0.270971051	0.448889733	0.907000703	0.000292137
101110881	SMIM4	0.26083779	0.466678651	0.906929918	0.000293002
101106975	LOC101106975	0.449385792	0.19259153	0.90688008	0.000293612
101111418	CPPED1	0.367505426	0.296143503	0.906875263	0.000293671
101116448	FAM20C	0.388433791	0.267315231	0.906674073	0.000296143
101114256	ACTG2	0.555017556	0.095841414	0.906471558	0.000298646
101112435	UNC93B1	0.293629032	0.410259493	0.906398848	0.000299549
101119902	CC2D1A	0.767679421	0.009524174	0.906386796	0.000299699
105610832	LOC105610832	0.621950867	0.054865401	0.905731692	0.000307926
101102931	C7	-0.406391472	0.243860958	-0.600084917	0.066639317
101102340	ARPIN	0.30158908	0.397076093	0.905462074	0.000311359
101111516	PPIF	0.577154467	0.080643212	0.905356759	0.000312708
101101873	ATP2B3	0.451645882	0.190088266	0.905332924	0.000313014
106990892	-	0.555261178	0.095664955	0.905331308	0.000313035
101113178	CDC25A	0.555261178	0.095664955	0.905331308	0.000313035
101114825	B4GALT2	0.348672789	0.323436426	0.905244839	0.000314146
101122493	AP1S1	0.302746791	0.395175881	0.905154971	0.000315305
105615522	-	0.361392641	0.304863343	0.905086389	0.000316191
101103683	IRF7	0.424092338	0.221914901	0.905014233	0.000317125

101121011	MED28	0.404958124	0.24568917	0.447731339	0.194436159
105609034	ANKRD16	0.404872939	0.245798062	0.640903461	0.045849754
101110862	PEPD	0.294833072	0.408252195	0.904705297	0.000321147
105612430	ENTPD8	0.552508804	0.097670598	0.904114823	0.00032894
105602735	-	0.552508804	0.097670598	0.904114823	0.00032894
101101789	-	0.552508804	0.097670598	0.904114823	0.00032894
101122558	PTPN23	0.411089969	0.23792168	0.9040694	0.000329545
105604738	VKORC1	0.246641014	0.492112623	0.904029727	0.000330074
105610540	LOC105610540	0.528765936	0.116082806	0.903948	0.000331166
443166	SLC2A8	0.393336949	0.26079303	0.903387698	0.000338725
101119270	ZNF205	0.578600118	0.07970878	0.903217402	0.000341047
106991907	PAM16	0.44447188	0.198100369	0.903091237	0.000342775
101103835	RBM42	0.320173461	0.367108996	0.90306304	0.000343162
101116186	ZDHHC24	0.387821417	0.268136028	0.902983542	0.000344255
101122808	RPL18	0.371362845	0.290710262	0.902724517	0.000347835
101101820	-	0.515670747	0.127103068	0.902684338	0.000348392
101103094	DCLK2	0.42388585	0.222164162	0.902561062	0.000350107
101118292	MAP1LC3A	0.271960437	0.447169539	0.902543667	0.00035035
101120767	PRXL2B	0.261930401	0.464745741	0.902413175	0.000352173
101109981	LOC101109981	0.182920856	0.612982656	0.902248801	0.000354479
101110146	BATF	0.531344157	0.113986132	0.902211818	0.000354999
101117555	EVA1B	0.382555497	0.275251011	0.902199132	0.000355178
101121461	HYAL3	0.501736617	0.139516206	0.902009011	0.000357863
101121541	CMIP	0.484906055	0.155464564	0.901821211	0.00036053
105616895	-	0.402843294	0.248400537	0.414012009	0.23426946
101113123	SNX8	0.687233129	0.028109051	0.901500902	0.000365113
101110896	NME9	0.496440527	0.144421549	0.90101418	0.000372157
101119507	UPB1	0.496440527	0.144421549	0.90101418	0.000372157
105610408	-	0.496440527	0.144421549	0.90101418	0.000372157
105608456	-	0.496440527	0.144421549	0.90101418	0.000372157
106990134	S100A3	0.410118991	0.239142338	0.900968713	0.000372821
101108848	SPEN	0.527676447	0.116975999	0.900742358	0.000376135
101110509	TCOF1	0.698661326	0.024596937	0.900627015	0.000377832

101106160	SERHL2	0.40091268	0.250890217	0.667183904	0.035071556
101119253	SNRPD3	0.559039802	0.09295449	0.900606975	0.000378127
101110576	SLC2A4RG	0.520553771	0.122921031	0.900603574	0.000378178
101106617	RCN3	0.297192922	0.40433158	0.90035723	0.000381824
106991479	-	0.400465541	0.251468809	0.576758351	0.080900479
101122930	SLC6A8	0.65815112	0.038558092	0.900316335	0.000382431
443011	BGN	0.565167419	0.088664184	0.900306844	0.000382572
101108454	TIMM17B	0.327969694	0.35488353	0.900147773	0.000384944
101115982	PLTP	0.533175459	0.112511379	0.899794684	0.000390246
101109549	RGS9	0.399840771	0.252278494	0.565419371	0.088490547
101114752	PDLIM2	0.399249777	0.253045737	0.899633142	0.000392689
101113264	LOC101113264	0.64423524	0.044374813	0.899469098	0.000395182
101102873	VEGFD	0.330044063	0.351665651	0.899287376	0.000397957
101112736	VASH1	0.325594325	0.358586453	0.899082481	0.000401103
101113858	LOC101113858	0.355021837	0.31409379	0.898958951	0.000403009
100820740	TNXB	0.365408679	0.299119347	0.898949203	0.000403159
101122089	LOC101122089	0.571578371	0.084313568	0.898625255	0.000408189
101119928	MYPOP	0.196574702	0.586227308	0.898611043	0.000408411
101117073	HIGD2A	0.362866271	0.302748932	0.89828697	0.000413491
101109204	ZNF404	-0.398870624	0.253538643	-0.50974496	0.132295008
101108451	OGN	0.530207721	0.114907364	0.898203963	0.000414799
101122988	NQO2	0.307254586	0.387818959	0.89815378	0.000415592
101104048	NANS	0.413083793	0.235426176	0.897936712	0.000419033
101106071	-	0.531199451	0.114103178	0.89773201	0.000422297
101114628	CCL1	0.397560851	0.255245468	0.477086881	0.163232107
101120093	LOC101120093	0.310162383	0.383108925	0.897674665	0.000423215
101122819	LOC101122819	0.570679352	0.084915203	0.897572428	0.000424854
101101780	-	0.466952592	0.173639392	0.897453457	0.000426768
101123224	SWT1	-0.396946451	0.256048308	-0.718279191	0.019291455
105605404	-	0.372006602	0.289808761	0.897441588	0.00042696
100329218	SREBF1	0.547991281	0.101020017	0.897241167	0.0004302
101120342	TMEM256	0.302398451	0.395747168	0.896692561	0.00043916
101123535	ACER2	0.394344892	0.259463239	0.896644559	0.00043995

101114820	HMGA1	0.555172347	0.095729273	0.896501118	0.000442319
101123511	EHBP1L1	0.187823199	0.603326786	0.896041791	0.000449965
101115424	GET4	0.522767889	0.121053316	0.895431584	0.000460273
101115544	GRAMD2A	0.395511555	0.257928729	0.483632972	0.156713705
101102052	MFAP5	0.416976599	0.230596611	0.895314127	0.000462276
101121995	DCTPP1	0.38161374	0.276534162	0.895229144	0.00046373
101111996	NECTIN2	0.432980045	0.211337556	0.895139797	0.000465262
101116367	KCTD5	0.394850728	0.258797297	0.608961955	0.061677575
443211	G6PD	0.199692555	0.580179131	0.894985464	0.000467917
101114379	LOC101114379	0.61973304	0.055991457	0.894815878	0.000470847
101117812	STK40	0.295829248	0.406594969	0.894731132	0.000472317
101104797	LOC101104797	0.632265392	0.049825458	0.894427487	0.000477609
101106756	KDM3A	-0.394392756	0.259400185	-0.892523614	0.000511787
443541	PGD	0.229448118	0.523682204	0.89435008	0.000478965
101118799	MAP3K14	0.312133767	0.379931673	0.894319275	0.000479505
101110287	CFAP46	0.475098354	0.165243905	0.893919108	0.000486566
101107029	RECQL4	0.305070194	0.391375628	0.893558137	0.000493001
101116497	PROSER2	0.400620468	0.251268251	0.893393627	0.000495954
101106884	C18H15orf39	0.366805385	0.297135286	0.893172265	0.000499948
101111733	LOC101111733	0.615880486	0.057983641	0.893011949	0.000502855
101104033	PCOLCE	0.611434811	0.060340002	0.893010976	0.000502872
101112632	ZNF593	0.329681632	0.352226809	0.892946225	0.00050405
105607079	KLF16	0.324589316	0.36015895	0.892653512	0.000509399
101120887	ITGB5	0.445332397	0.197129117	0.892633239	0.000509771
101123025	GGA1	0.308626789	0.385592778	0.892631871	0.000509797
101109152	SLC66A1	0.475458072	0.164878882	0.892511079	0.000512017
101117965	LOC101117965	0.54018367	0.106978461	0.892424134	0.00051362
101102940	MTCH1	0.412104482	0.236650047	0.891868666	0.000523947
101117811	THOC6	0.315151988	0.375092391	0.891855863	0.000524187
100144766	FCGRT	0.392062288	0.262480071	0.688244159	0.027785321
105604778	-	0.619600472	0.056059245	0.891822283	0.000524816
101103741	METRNL	0.238280569	0.507361395	0.891455894	0.000531719
101102330	RPS9	0.26188085	0.464833324	0.891279239	0.000535071

101105179	LOC101105179	0.375907409	0.284378337	0.89106645	0.000539129
101109696	NUP42	-0.391067414	0.26380097	-0.782063648	0.007515505
105615175	LOC105615175	0.391063104	0.2638067	0.625891471	0.05290183
101123086	SLX4	0.464632292	0.176076304	0.890910967	0.000542108
101108518	ZMAT5	0.590778158	0.072114196	0.890791621	0.000544403
101104103	ADGRL2	0.525058618	0.119139672	0.890779557	0.000544636
101105403	PTPA	0.300149832	0.399444517	0.890763024	0.000544954
105604846	C24H16orf91	0.491929244	0.148681822	0.890730382	0.000545584
106990972	-	0.662685617	0.036779933	0.89029752	0.00055398
101103747	NXT1	0.213589563	0.553510861	0.890115097	0.000557547
101122959	MAP2K3	0.296510405	0.405463651	0.890060111	0.000558626
101110973	LOC101110973	0.256326107	0.474697628	0.88940861	0.00057152
101119624	-	0.445794762	0.196608403	0.889330123	0.000573088
101113927	ANKRD23	0.618854974	0.056441466	0.889074325	0.00057822
101118911	EHD1	0.228928379	0.52464922	0.888845439	0.00058284
105608252	TMEM129	0.286706005	0.421891279	0.888567095	0.000588495
101109335	LOC101109335	0.274231149	0.44323291	0.88834715	0.000592992
101115767	PHF19	0.241243143	0.501935389	0.888334633	0.000593248
101122793	RBPMS2	0.458629902	0.182474034	0.887935518	0.000601475
101117080	STON2	0.416809936	0.23080222	0.887921344	0.000601769
101107229	TKT	0.169004903	0.640681603	0.887840997	0.000603436
101104133	-	0.605046702	0.063834951	0.887526746	0.000609987
100620046	TOB2	-0.387916235	0.268008849	-0.605935511	0.063340924
101118018	RNF44	0.387869217	0.26807191	0.652670841	0.040783368
101111400	PEX6	0.314080004	0.376807672	0.887107887	0.0006188
101111511	SPATA1	0.4282701	0.21690603	0.887048055	0.000620067
101106378	TKFC	0.506757066	0.134961652	0.88672988	0.000626833
101118341	LDLRAP1	0.579908214	0.078869313	0.886547189	0.000630743
101108888	LIMS2	0.423961767	0.222072501	0.886341266	0.000635171
101109473	CIAO2B	0.489891361	0.150631061	0.88631189	0.000635804
105603685	-	0.26233258	0.464035153	0.88629279	0.000636216
101103102	GPR137	0.409205662	0.240293727	0.885967412	0.000643268
105605471	LOC105605471	0.216197187	0.548561003	0.885935758	0.000643957

101123371	FAM131B	0.331318797	0.349695549	0.885823725	0.0006464
101121716	SEMA3B	0.477240854	0.163076949	0.885767132	0.000647637
101115315	LOC101115315	0.581236216	0.07802294	0.885734092	0.000648359
101101854	HTRA1	0.385672375	0.271027398	0.658155392	0.03855639
678680	LGMN	0.385532994	0.27121551	0.589964644	0.072606213
105607505	LOC105607505	0.343060903	0.331812874	0.885488628	0.000653748
101120905	SWI5	0.547333196	0.101513925	0.885388355	0.000655958
101117241	MIOX	0.309220569	0.384631401	0.885147494	0.00066129
101118708	CRYL1	0.257848881	0.471984359	0.884869834	0.000667476
101118002	MORN2	-0.385055797	0.271860085	-0.684865392	0.028877261
101109726	MINDY3	0.3848615	0.272122772	0.366185156	0.298015471
101122463	SELENOH	0.508179982	0.133687639	0.884479933	0.000676233
101123583	MORN3	0.384763554	0.272255245	0.503905332	0.137537365
101123041	SPARCL1	0.384615081	0.272456125	0.3882542	0.267555804
101114989	ZNF687	0.274823743	0.442208165	0.883937246	0.000688562
101114450	NHSL1	0.348215138	0.324115374	0.883741755	0.000693043
101111082	CLCNKA	0.45510901	0.186289745	0.883475149	0.000699189
101117701	ULK1	0.208810195	0.562628131	0.883111011	0.000707646
105602272	-	0.323537672	0.361808102	0.882965061	0.000711057
101106926	PKN3	0.323537672	0.361808102	0.882965061	0.000711057
105606937	-	0.323537672	0.361808102	0.882965061	0.000711057
101116674	MED29	0.450126547	0.19176896	0.882723752	0.000716723
101115632	LOC101115632	0.508619294	0.1332958	0.882442299	0.000723373
101117721	TAOK2	0.638248727	0.047048168	0.882047344	0.00073278
101113354	RTN2	0.365787523	0.298580492	0.881930192	0.000735588
101108302	ATN1	0.520834541	0.122683204	0.881845852	0.000737614
101102298	-	0.574429317	0.082423839	0.881838873	0.000737781
101121686	TRAPPC9	0.417379222	0.23010033	0.881547078	0.000744824
105612811	C17H22orf39	0.55635857	0.094872662	0.881251429	0.000752009
101112187	SH2B1	0.40795108	0.241880379	0.880454102	0.000771638
101102338	GATB	0.470960391	0.169477687	0.880073516	0.000781138
101113694	MRPS12	0.154991699	0.668983606	0.879852302	0.000786699
101112531	RCC1L	0.596387329	0.068780752	0.878814045	0.000813186

105603758	GPSM3	0.235726547	0.51205874	0.8785156	0.000820919
101105185	LOC101105185	0.285014132	0.424757085	0.878345724	0.000825344
101111913	GLYCTK	0.46456789	0.176144231	0.878273809	0.000827223
101115735	PPIL2	0.479784186	0.160526846	0.877782914	0.00084013
101117428	MRPL57	0.649732295	0.042011394	0.877512487	0.000847302
100642181	SRD5A1	0.231489684	0.519890786	0.876749126	0.00086779
101112850	RITA1	0.430200705	0.214613453	0.876665755	0.000870049
105608522	LOC105608522	0.440467437	0.202656704	0.876531624	0.000873693
101115381	LOC101115381	0.267991732	0.454087745	0.876151106	0.000884091
101104951	APCDD1	0.267991732	0.454087745	0.876151106	0.000884091
101114707	ZNF219	0.267991732	0.454087745	0.876151106	0.000884091
101105557	SHB	0.267991732	0.454087745	0.876151106	0.000884091
101107767	RENBP	0.627105851	0.052306249	0.876060062	0.000886592
101112212	GLT8D2	0.624983048	0.053350287	0.876014573	0.000887843
101112621	TBL3	0.400279874	0.251709277	0.875863224	0.000892017
101112550	OST4	0.649883428	0.041947638	0.875817275	0.000893286
101118176	PARVB	0.367122226	0.296686182	0.875757336	0.000894945
101110137	VAV2	0.423166545	0.223033716	0.875704422	0.000896411
101115266	RNF144A	0.580432517	0.078534454	0.875564433	0.000900298
101110786	PPP1R14A	0.40766489	0.24224314	0.875453204	0.000903395
101122497	SIK1	0.475372699	0.16496547	0.875445016	0.000903623
101106601	WDFY2	0.647888844	0.042794315	0.875382953	0.000905355
105610672	-	0.656283306	0.039307086	0.87533602	0.000906666
101108149	FAM107B	-0.375315489	0.285198808	-0.740756678	0.01425294
105614947	-	0.656283306	0.039307086	0.87533602	0.000906666
101105455	CD248	0.656283306	0.039307086	0.87533602	0.000906666
105606176	NKX1-2	0.656283306	0.039307086	0.87533602	0.000906666
101120648	EFEMP1	0.656283306	0.039307086	0.87533602	0.000906666
101108893	CAPZB	0.43271068	0.211653778	0.875249193	0.000909095
101111979	MED11	0.296642208	0.405244915	0.874721109	0.000923973
101111216	CHMP4B	0.514988039	0.12769469	0.874348368	0.00093458
101121420	LOC101121420	0.631025636	0.050414248	0.874082002	0.000942214
101116874	CCDC88B	0.362269212	0.303604671	0.873994755	0.000944725

101122917	TNK2	0.20679869	0.566482523	0.873193032	0.000968022
105605689	-	0.20679869	0.566482523	0.873193032	0.000968022
101110710	LOC101110710	0.369236795	0.293698186	0.873135061	0.000969723
105605967	-	0.371611887	0.290361332	0.873097141	0.000970836
101122787	HSD17B11	0.372687603	0.288856741	0.536368141	0.109969035
101104282	MYBPHL	0.372455322	0.289181275	0.542689508	0.105042588
101118511	LOC101118511	-0.372374448	0.289294314	-0.391036843	0.263841617
100913151	FOXO3	0.375161905	0.285411901	0.872919913	0.000976053
101109713	HNF4G	0.276161991	0.439897981	0.87269334	0.000982753
105603310	LOC105603310	0.276161991	0.439897981	0.87269334	0.000982753
101111408	NUDT8	0.313801275	0.377254295	0.872656952	0.000983832
101102254	LOC101102254	0.391046855	0.263828305	0.872568401	0.000986461
101116898	APLF	-0.37128399	0.290820791	-0.6705603	0.03382514
654329	NUDT16	0.277157791	0.438182549	0.872460759	0.000989664
106991289	-	0.16883997	0.641012383	0.872332643	0.000993487
101107006	PTDSS2	0.695214475	0.025622519	0.872214722	0.000997014
443226	TIMELESS	0.387623542	0.268401545	0.872127527	0.000999629
101116309	HMG20B	0.43075781	0.213954494	0.871717796	0.00101198
101119470	NDUFA2	0.477219874	0.163098086	0.871593374	0.001015753
101112132	GTPBP3	0.385219559	0.271638789	0.870766583	0.001041084
101112099	CALHM2	0.630094975	0.05085927	0.870740566	0.001041888
101121454	SERPING1	0.368634186	0.294548058	0.637259814	0.047499881
101113261	IL34	0.450263078	0.191617574	0.870441457	0.001051169
101118499	FXYD4	0.399766694	0.252374591	0.870439579	0.001051228
101111251	ZMIZ1	0.367796673	0.2957314	0.593874311	0.070261489
105602860	-	0.694788262	0.025751338	0.870351112	0.001053985
101104745	LOC101104745	0.367432203	0.296247159	0.486612467	0.153799708
101107041	TSEN34	0.450011821	0.191896222	0.869887664	0.001068512
100145856	HEXA	0.235332501	0.512785075	0.86987721	0.001068841
101115199	RDH5	0.480038639	0.160273044	0.869789002	0.001071624
443076	MET	0.478806751	0.161504025	0.86970188	0.001074377
101107424	RCC2	0.299477767	0.400552782	0.869537495	0.001079585
101111728	TMEM51	0.541807657	0.105721316	0.869251691	0.001088684

101105362	SNX33	0.365982027	0.298304038	0.869111809	0.001093158
101112992	ABR	0.260507344	0.467263934	0.869015051	0.00109626
101105013	MNT	0.416655024	0.230993426	0.868490518	0.00111319
100462690	CRISP2	0.415594836	0.232304404	0.867936938	0.001131262
101104146	LOC101104146	0.415594836	0.232304404	0.867936938	0.001131262
101108900	-	0.415594836	0.232304404	0.867936938	0.001131262
106991326	-	0.415594836	0.232304404	0.867936938	0.001131262
101101812	-	0.415594836	0.232304404	0.867936938	0.001131262
106991155	-	0.415594836	0.232304404	0.867936938	0.001131262
101120100	AP5S1	0.605402244	0.063637028	0.867879061	0.001133164
101109942	PHC2	0.232736577	0.51758074	0.867670319	0.001140042
101123475	EMC6	0.483541681	0.156803511	0.867358285	0.001150381
443411	FXYD6	0.534627037	0.111350959	0.867307174	0.001152081
101109813	ARL4D	0.464071623	0.176668179	0.867014227	0.001161859
101118390	MAGI1	0.364596942	0.30027567	0.629233134	0.051273705
101120414	SPTB	0.540164198	0.106993592	0.866536392	0.001177938
101119881	-	0.221665535	0.53823798	0.866490391	0.001179494
101116736	RNF146	-0.363873894	0.301307652	-0.761962177	0.010418453
101108703	KCNK7	0.221665535	0.53823798	0.866490391	0.001179494
105604626	LOC105604626	0.499427379	0.141642331	0.866316441	0.001185393
101117580	BCL2L13	0.043413377	0.905212021	0.866152373	0.001190977
105604784	LOC105604784	0.703826091	0.023113512	0.86608482	0.001193281
101110222	CDCA3	0.537908106	0.108755781	0.865949124	0.00119792
101110364	MMP11	0.249552621	0.486848688	0.865610204	0.001209563
101122030	DNAAF4	-0.362696715	0.302991819	-0.740817786	0.014240659
101102938	TMEM40	0.702007331	0.02362867	0.86543174	0.001215727
101113535	NDN	0.475003867	0.165339865	0.865209449	0.001223437
105603536	LOC105603536	-0.362561516	0.303185565	-0.686862231	0.028228456
101114290	NUAK2	0.604400531	0.064195695	0.865170565	0.001224789
101117602	SPSB1	0.604400531	0.064195695	0.865170565	0.001224789
105604881	C24H7orf50	0.23790068	0.50805894	0.865067056	0.001228394
101112505	CHMP2A	0.297583164	0.403684977	0.864987268	0.001231178
101113026	PMVK	0.421268894	0.225337042	0.864915997	0.001233669

101112822	LOC101112822	0.329260846	0.352878884	0.864864042	0.001235487
101109073	GPBAR1	0.24779457	0.490024186	0.864709864	0.001240894
101109176	SLC25A23	0.676031494	0.031869784	0.864417842	0.001251181
101102599	PPP1R14B	0.179939948	0.618880415	0.864368907	0.001252911
101115487	RPS10	0.303597752	0.393781956	0.864270911	0.00125638
105614765	LOC105614765	-0.361273408	0.305034763	-0.649692106	0.042028359
101104925	TFAP2C	0.361160244	0.305197504	0.429500743	0.215443036
101119880	HSD17B10	0.301559424	0.397124827	0.864220124	0.001258181
101112206	LOC101112206	0.489487808	0.151018888	0.864176594	0.001259726
101121337	BRD1	0.229389009	0.523792144	0.864123038	0.001261629
101113953	-	0.32927619	0.352855095	0.864019678	0.001265307
101104940	ZFAND3	0.222995497	0.535739143	0.863942996	0.001268041
101115197	HAAO	0.222428989	0.536802972	0.863720634	0.001275992
106990843	LOC106990843	0.327116539	0.356211276	0.863711595	0.001276316
101116704	SIT1	0.405412904	0.245108278	0.863444296	0.001285924
106991028	-	0.359566992	0.307493635	0.675249861	0.032144303
101107815	CNOT3	0.468780532	0.171733793	0.863195699	0.001294907
101102115	SLC12A9	0.352000906	0.318521272	0.863169725	0.001295849
101114403	POU2F1	-0.358771002	0.308644189	-0.569777581	0.085521445
101105422	ALDOC	0.569250788	0.08587688	0.863145726	0.001296719
106991118	-	0.357809343	0.310037226	0.662617472	0.036806237
101122137	DGCR8	0.770633392	0.009084007	0.863139869	0.001296931
101110880	OXSR1	-0.35699252	0.311223052	-0.63387625	0.049067286
101108250	RIC3	0.348634733	0.323492856	0.862846614	0.001307598
100913160	YAP1	0.356710827	0.311632552	0.667450664	0.034971967
101119939	TM2D3	0.356562262	0.311848637	0.687820239	0.027920748
101102893	NAGK	0.601204457	0.065999654	0.862504748	0.001320114
101119596	KCNQ1	0.362140717	0.303789006	0.862402608	0.00132387
101114311	CCDC197	0.362140717	0.303789006	0.862402608	0.00132387
101102139	FAM83F	0.362140717	0.303789006	0.862402608	0.00132387
101104665	MRPL52	0.657505137	0.038816033	0.862298191	0.001327718
101110078	RABIF	0.595561792	0.069264912	0.86210031	0.001335033
101121684	ZFP36L1	0.32523421	0.359149513	0.861845637	0.00134449

105616119	-	0.354561331	0.314766613	0.616398475	0.057713105
106991597	LOC106991597	0.301557767	0.39712755	0.861360374	0.001362643
101104839	TSPOAP1	0.301557767	0.39712755	0.861360374	0.001362643
101107580	TMEM222	0.193132564	0.59293146	0.861314706	0.001364361
494435	SLC9A1	0.742469687	0.013911452	0.861023627	0.001375345
101122149	TSPAN4	0.466492752	0.174120739	0.86092918	0.001378923
101106199	LOC101106199	0.254413104	0.478115852	0.860835633	0.001382474
100125621	PLIN3	0.158396651	0.662070569	0.860596868	0.001391566
101122311	LOC101122311	0.352662468	0.317548915	0.490585879	0.149965021
101120560	ZNF74	0.731459105	0.016209096	0.860564884	0.001392787
101109236	TELO2	0.460422985	0.180548692	0.860522011	0.001394425
101117257	UNC45B	0.353854568	0.315800698	0.859972517	0.001415546
101116871	CHCHD6	0.280314092	0.432765644	0.859896291	0.001418494
100145868	ERP29	0.351455936	0.31932343	0.68000142	0.030500077
100820747	GATA1	0.388055703	0.267821839	0.859676918	0.001427003
101107806	NOTUM	0.368066952	0.29534924	0.859309802	0.001441326
101108757	ALDH8A1	0.479102297	0.161208181	0.858995239	0.001453681
101108351	TMEM258	0.37308321	0.288304466	0.858988707	0.001453938
106991016	LOC106991016	0.461569659	0.179323764	0.858800353	0.001461374
101119209	HK3	0.144080771	0.691285058	0.858610835	0.001468883
101103509	SETD1B	0.370310196	0.292187597	0.858403507	0.00147713
101119667	BACE1	0.505739255	0.135877517	0.858008427	0.001492939
101115304	EXOC3L2	0.349109123	0.322789792	0.32153843	0.364953622
101117179	HES2	0.349079135	0.322834211	0.63871188	0.0468376
105602721	LOC105602721	0.580064045	0.078769691	0.857941592	0.001495625
101103527	STX4	0.311327555	0.381229468	0.857788717	0.001501783
106991156	LOC106991156	0.382358881	0.275518634	0.857298867	0.001521638
101107434	SCUBE1	0.403427067	0.247650439	0.857234558	0.001524259
101108506	KIF21B	0.347478464	0.325209819	0.695553536	0.025520357
101115294	TNRC6C	0.584591086	0.07591103	0.856495338	0.001554619
101104572	LPAR2	0.368851946	0.294240795	0.856461022	0.001556039
101103332	STARD3	0.300626371	0.398659575	0.85615561	0.001568718
101114421	RETREG2	0.347287336	0.325494081	0.687749135	0.027943507

101108207	TEX261	0.300963275	0.398105085	0.855836369	0.001582051
101113214	SPACA9	0.506690186	0.135021717	0.855401177	0.001600358
101116590	JCAD	-0.347026165	0.325882728	-0.716647505	0.01969928
101108553	ZNF395	0.506690186	0.135021717	0.855401177	0.001600358
101121190	LOC101121190	0.506690186	0.135021717	0.855401177	0.001600358
105616094	-	0.346669201	0.326414312	0.504884762	0.136649363
101110640	PCBP3	0.359707355	0.307290985	0.85488398	0.001622314
101111820	WNK4	0.519690834	0.123653779	0.854754806	0.001627832
101115643	LDHD	0.210036982	0.560282369	0.854591421	0.00163483
105606266	LOC105606266	0.383225189	0.274340525	0.854570295	0.001635737
101102765	LOC101102765	0.363383407	0.302008777	0.854350842	0.001645175
101111570	TOMM40	0.608292701	0.062042898	0.854137505	0.001654388
101108523	WDR6	0.726903114	0.017232639	0.854007179	0.001660034
780451	CES5A	0.60599365	0.063308696	0.853600211	0.001677756
101107813	PIEZO1	0.531182283	0.114117069	0.853247145	0.001693241
101113838	TFPI2	0.343389277	0.331319686	0.443247493	0.199487108
101117853	DLL1	0.343332488	0.331404951	0.674420984	0.032437167
101102793	SEC61B	0.606050287	0.063277312	0.853114944	0.001699066
101116606	NUBPL	-0.34248346	0.332681058	-0.706330526	0.022416861
101122452	SLC16A5	0.122417823	0.736188175	0.853002268	0.001704042
101115420	LOC101115420	0.365063543	0.299610705	0.852809711	0.00171257
101120343	PCGF2	0.308506491	0.385787693	0.8527114	0.001716936
101104363	SERPINB11	0.400260846	0.251733929	0.852438549	0.001729096
101120856	PSENEN	0.314610535	0.375958287	0.852308082	0.001734932
101121257	RASGEF1C	0.33123823	0.3498199	0.851829757	0.001756451
101103943	FBRS	0.245650677	0.493908597	0.851274515	0.001781672
101109872	BRD3	0.339064614	0.337845181	0.640145754	0.046189696
101110964	LOC101110964	0.339050466	0.337866636	0.400003128	0.252067944
101105839	MIXL1	0.258423502	0.470962279	0.85097682	0.001795302
105605805	LOC105605805	0.360858602	0.305631521	0.850939751	0.001797004
101110900	LIMK1	0.446675979	0.195618201	0.850488295	0.001817832
101102224	ZMYND19	0.371809802	0.290084196	0.849889268	0.001845736
101112930	LOC101112930	0.443330203	0.199393253	0.849272753	0.001874778

101122622	ZNF792	-0.337277051	0.340561505	-0.694124984	0.025952689
101119899	MICALL1	0.74816263	0.012817639	0.849214178	0.001877554
106991838	-	0.337195818	0.340685209	0.695737421	0.025465068
101114037	COQ8A	0.60540056	0.063637964	0.849151027	0.001880551
101103928	ADSS1	0.560206278	0.092127774	0.84852017	0.001910676
101120812	LOXL2	0.731228982	0.016259754	0.848424939	0.001915253
101102365	JTB	0.32057299	0.366477461	0.848376589	0.00191758
101117373	ZBTB22	0.399265755	0.253024977	0.847874887	0.001941849
101114255	NCK2	0.702876959	0.023381376	0.847827367	0.001944159
101118428	ZER1	0.309573971	0.384059768	0.847443944	0.001962871
101106282	LOC101106282	0.33510347	0.343879376	0.63465283	0.04870454
101119357	PHLPP1	0.57960065	0.079066175	0.847259742	0.001971907
101102543	LOC101102543	0.331117052	0.350006973	0.847106789	0.001979433
101106927	CCDC142	0.43194988	0.212548391	0.846771507	0.001996002
101104888	MAP3K15	0.333785581	0.345899049	0.846145875	0.002027188
101119586	POP5	0.439008712	0.204331436	0.845696282	0.002049816
101117172	MICU2	-0.332185557	0.348359171	-0.85663188	0.001548978
101107810	RGS19	0.250648945	0.484872942	0.845124949	0.002078833
105602113	LOC105602113	0.490355097	0.150186142	0.844878864	0.002091423
105605734	-	0.331559458	0.349324237	0.387453822	0.268629396
101123189	C1QC	0.565541298	0.088406597	0.844535718	0.002109069
101122687	CBX7	0.298653617	0.401913849	0.8441465	0.002129215
101106184	TMEM107	0.576652057	0.080969604	0.844112231	0.002130996
105616708	LOC105616708	0.439395396	0.203886713	0.843335837	0.002171621
105614874	-	0.330860938	0.350402527	0.608199489	0.062093892
101122693	MAST1	0.423942608	0.222095631	0.843174589	0.002180128
101114988	IGHMBP2	0.152871361	0.673299882	0.842495462	0.002216221
105604572	-	0.239941175	0.504316944	0.841931783	0.002246505
101114561	OTUB2	0.440376371	0.202761021	0.841743864	0.002256667
101107127	PRRG2	0.186150813	0.606614657	0.840444761	0.002327828
101113439	MAP4	0.259454586	0.469130732	0.839832592	0.002361916
101110190	TTC39A	0.475006005	0.165337693	0.839819336	0.002362658
101108085	LOC101108085	0.414377408	0.233814994	0.839431102	0.002384468

101112273	PIK3AP1	-0.328761089	0.353654129	-0.706027089	0.022500487
101112932	ABCC10	0.390870174	0.26406328	0.839315211	0.002391006
101110749	MLF2	0.496238958	0.144610294	0.838555913	0.002434164
101110596	GPX2	0.589249446	0.073040567	0.83843351	0.002441173
443542	ATP5MC2	0.263201389	0.46250175	0.838417047	0.002442117
106991502	LOC106991502	0.591787018	0.071507054	0.838397472	0.00244324
101120574	LOC101120574	0.545222696	0.103108212	0.838382922	0.002444075
105608356	LOC105608356	-0.327407325	0.355758452	-0.632976837	0.049489652
101112415	SCAF1	0.460224131	0.180761619	0.838373252	0.002444629
100499498	MKRN1	0.22593464	0.530233457	0.837979365	0.002467308
101122734	TUBB2A	0.474943259	0.165401435	0.836977448	0.002525677
101120449	ILRUN	0.725087577	0.017652736	0.836901009	0.00253017
101113945	ATXN7L3	0.334308771	0.345096537	0.836267162	0.002567655
105609508	LOC105609508	0.247440885	0.490664106	0.836211083	0.00257099
101123140	TGM2	0.27535075	0.441297746	0.836017909	0.002582504
101107219	DPAGT1	0.326171715	0.357684594	0.654459636	0.040047774
105605768	LOC105605768	0.573830651	0.082818374	0.83530979	0.002625028
101117884	MSH5	0.433133789	0.21115719	0.835306856	0.002625205
106991485	-	0.597639403	0.068050663	0.835146023	0.002634934
101110457	LOC101110457	0.377886229	0.281644717	0.834183503	0.0026937
101117829	SELENON	0.665289919	0.035784165	0.833666811	0.002725633
101104324	LRRC63	0.665289919	0.035784165	0.833666811	0.002725633
100174906	ITM2C	0.221344697	0.538841494	0.833549986	0.00273289
101112654	AGPAT4	0.062915639	0.862915531	0.833463754	0.002738256
101112769	ZNRD1	0.407733425	0.242156241	0.833272172	0.002750205
101102887	RNF181	0.286311092	0.422559398	0.833121071	0.002759656
101106993	ARMC5	0.70315128	0.023303739	0.832771466	0.002781611
101113269	CAPN5	0.322400919	0.363594954	0.649928318	0.041928713
106990156	-	0.322383439	0.363622465	0.659982538	0.037833094
101120758	COL14A1	0.322383439	0.363622465	0.659982538	0.037833094
101117031	LOC101117031	0.322383439	0.363622465	0.659982538	0.037833094
101107824	SYN2	0.322383439	0.363622465	0.659982538	0.037833094
101122467	CDC42BPB	0.322383439	0.363622465	0.659982538	0.037833094

105605666	LOC105605666	0.322383439	0.363622465	0.659982538	0.037833094
101119941	LOC101119941	0.322383439	0.363622465	0.659982538	0.037833094
101111528	LOC101111528	0.401249799	0.25045448	0.832400253	0.002805061
101102688	LOC101102688	0.761949875	0.010420438	0.832357152	0.002807793
101108223	LOC101108223	0.761949875	0.010420438	0.832357152	0.002807793
101121951	NT5C	0.277985803	0.436758501	0.831864556	0.002839152
101110686	CORO6	0.337681796	0.339945494	0.831745349	0.002846779
101113830	ASIC4	0.763513319	0.010170224	0.831469062	0.002864512
101110272	KLF13	0.23468878	0.513972548	0.831157936	0.002884575
101112433	BANF1	0.318472152	0.369804301	0.537309076	0.109226718
106991149	-	0.431683047	0.21286267	0.831132671	0.002886209
101119393	LOC101119393	0.318128262	0.370350299	0.512041322	0.130267787
105609801	LOC105609801	0.605437169	0.063617607	0.830713066	0.00291344
101114785	RNASET2	0.326668757	0.356909147	0.830647948	0.002917682
101113367	KIFC1	0.770398368	0.00911849	0.830369736	0.002935857
101109030	LRRN2	0.394647189	0.259065147	0.830220481	0.002945641
101117612	EML2	0.264179563	0.460778027	0.829756675	0.002976195
101111219	EIF3K	0.195591994	0.588138437	0.828428382	0.003064959
101116307	KIAA0895	0.523820907	0.12017128	0.828233342	0.003078151
101114770	SLC6A12	0.426166262	0.219420224	0.82783629	0.003105133
101105058	SMPDL3B	0.441610597	0.201349851	0.827181132	0.003150026
443369	PADI3	0.23513423	0.513150703	0.827064331	0.003158078
101114095	ACVRL1	0.312523818	0.379304568	0.826715825	0.003182191
105611484	MEIKIN	0.021249196	0.953538365	0.826544944	0.003194063
105604364	LOC105604364	0.465232312	0.175444194	0.825961611	0.003234829
101120504	CUEDC1	0.309036176	0.384929823	0.825623989	0.003258594
101105895	ALDH4A1	0.433324603	0.210933458	0.825292409	0.003282055
101102744	SOX15	0.433324603	0.210933458	0.825292409	0.003282055
101104357	HRAS	0.362817724	0.302818465	0.824992213	0.0033034
100174988	ITM2B	-0.313327684	0.378013755	-0.582271606	0.07736715
101104499	FBXL4	-0.313160252	0.378282433	-0.754171891	0.011729931
101122289	SHPK	0.680428211	0.030355255	0.824877338	0.003311594
106990166	-	0.492345215	0.148285839	0.82479145	0.003317731

101115341	BFSP2	0.036987425	0.919200607	0.824686785	0.003325219
101110365	ARID3B	0.471956087	0.168453081	0.824389357	0.003346566
105616901	LOC105616901	0.378894675	0.280257097	0.823989677	0.003375407
101113422	MAFG	0.158527242	0.661805885	0.823801519	0.003389046
101123030	MINDY4	0.62185807	0.054912214	0.823755401	0.003392395
101122916	MAP11	0.418845665	0.228297884	0.823723866	0.003394686
105603073	-	0.311555908	0.380861659	0.58350387	0.07659133
101107533	KIFC2	0.431295043	0.213320142	0.823502788	0.003410781
101120042	LOC101120042	0.50142804	0.139799175	0.822462182	0.003487276
554321	LTF	0.516444621	0.126434498	0.821975848	0.003523446
105609231	LOC105609231	0.30972318	0.383818545	0.821837239	0.003533803
106992027	LOC106992027	0.30972318	0.383818545	0.821837239	0.003533803
101111990	LOC101111990	0.310796768	0.382085084	0.573824916	0.08282216
101109630	TEPSIN	0.30972318	0.383818545	0.821837239	0.003533803
105609473	-	0.30972318	0.383818545	0.821837239	0.003533803
105604792	LOC105604792	0.30972318	0.383818545	0.821837239	0.003533803
101120736	LOC101120736	0.30972318	0.383818545	0.821837239	0.003533803
101110552	C1H1orf56	0.30972318	0.383818545	0.821837239	0.003533803
106990872	-	0.30972318	0.383818545	0.821837239	0.003533803
101109798	PALM	0.30972318	0.383818545	0.821837239	0.003533803
106990487	LOC106990487	0.30972318	0.383818545	0.821837239	0.003533803
101113436	BCR	0.30972318	0.383818545	0.821837239	0.003533803
101114979	PPM1F	0.30972318	0.383818545	0.821837239	0.003533803
101116143	ZBTB7A	0.30972318	0.383818545	0.821837239	0.003533803
101111598	CALN1	0.30972318	0.383818545	0.821837239	0.003533803
101118415	LOC101118415	0.30972318	0.383818545	0.821837239	0.003533803
101114594	OBSL1	0.30972318	0.383818545	0.821837239	0.003533803
443523	CSF3	0.30972318	0.383818545	0.821837239	0.003533803
101123419	LOC101123419	0.30972318	0.383818545	0.821837239	0.003533803
101104825	TSSK6	0.30972318	0.383818545	0.821837239	0.003533803
101102149	CFAP99	0.30972318	0.383818545	0.821837239	0.003533803
106991366	LOC106991366	0.30972318	0.383818545	0.821837239	0.003533803
105612738	ZNF580	0.30972318	0.383818545	0.821837239	0.003533803

101105210	KCNC4	0.30972318	0.383818545	0.821837239	0.003533803
101104701	KLC2	0.30972318	0.383818545	0.821837239	0.003533803
101111922	LOC101111922	0.30972318	0.383818545	0.821837239	0.003533803
101109548	RNF112	0.30972318	0.383818545	0.821837239	0.003533803
101106970	LOC101106970	0.30972318	0.383818545	0.821837239	0.003533803
101108977	RBP1	0.30972318	0.383818545	0.821837239	0.003533803
105611776	LOC105611776	0.30972318	0.383818545	0.821837239	0.003533803
101108948	GDPD5	0.396478806	0.256660318	0.821814157	0.00353553
100302305	ARF5	0.535434357	0.110708837	0.821251878	0.003577786
101122519	MKNK2	0.430589552	0.214153391	0.821164427	0.00358439
443061	BCL2L1	0.492060958	0.148556367	0.82093786	0.003601542
101123660	P2RY6	0.331585995	0.349283304	0.820796407	0.00361228
106990979	-	0.254879782	0.477280986	0.820050027	0.003669319
101102535	AKAP4	0.273678956	0.444188764	0.81917214	0.003737232
101114977	AACS	0.362636557	0.30307802	0.817761552	0.003848236
101107734	ICE1	0.30165159	0.396973379	0.817108551	0.003900415
101108179	MFSD13A	0.480741119	0.159573615	0.816942463	0.003913766
101111832	LOC101111832	0.303194623	0.394442009	0.816818677	0.003923739
101101948	MAZ	0.465528064	0.175133121	0.81672562	0.003931248
101117272	LOC101117272	-0.309541053	0.384112996	-0.664196483	0.036199996
101104656	RAD23A	0.499099563	0.141945748	0.81657561	0.003943374
101121114	EMP3	0.266379676	0.456911524	0.81642527	0.003955553
101112388	TMEM131	0.30915338	0.384740126	0.6819691	0.02983629
101102719	LOC101102719	0.595123402	0.06952292	0.816420022	0.003955979
101122755	SLX1A	0.268316835	0.453519228	0.81628741	0.003966746
101108010	SNRNP25	0.598569155	0.067511809	0.815673009	0.004016902
443522	PMCH	0.311945521	0.380234507	0.81528567	0.004048755
101111834	STOML1	0.284784502	0.425146743	0.815187235	0.004056879
101109417	LOC101109417	0.297386291	0.40401112	0.814837667	0.004085822
101105758	MYO5A	-0.308281903	0.386151715	-0.686462255	0.028357609
105615140	-	0.251428904	0.483469452	0.814579129	0.004107323
101115396	EXOSC6	0.510880915	0.131289778	0.813440868	0.00420295
101111324	NOTCH4	0.50363838	0.137780009	0.813399033	0.004206495

101116767	PISD	0.451105737	0.190684783	0.813355499	0.004210185
101106191	GPR158	0.162432616	0.653906099	0.812716259	0.004264649
101111107	CHD7	-0.30529828	0.391003517	-0.523091322	0.120781973
101111754	NHLRC1	0.433001871	0.211311945	0.812535391	0.00428015
101121861	HSF2	-0.304658638	0.392047492	-0.872136064	0.000999373
101105462	ABAT	-0.304559203	0.392209904	-0.462048865	0.178813317
101123467	SH3BP2	-0.046433646	0.898645118	0.81229919	0.004300454
105602360	-	0.088497852	0.807920002	0.811748744	0.004348039
101114642	TRIM44	0.43535274	0.208563886	0.810996445	0.00441368
101110763	NDRG2	0.482236642	0.158090719	0.810374987	0.004468437
101114124	H6PD	0.581820083	0.077652693	0.81009627	0.004493152
101106984	FCRLB	0.337043127	0.340917792	0.810010762	0.004500754
101110356	-	0.623230686	0.054222474	0.809334103	0.004561236
101105785	LOC101105785	0.053022727	0.884338323	0.809212089	0.004572203
101115214	FAM81A	-0.302922643	0.39488763	-0.565285734	0.088582618
101116960	REEP4	0.439799859	0.203422144	0.808962528	0.004594694
101122020	ATP6V0E2	0.17820371	0.622324654	0.807858832	0.004695111
100302549	SLC1A5	0.347128788	0.325729987	0.806174154	0.004851396
101113548	SHC1	0.407305632	0.24269895	0.805928026	0.004874535
101105685	LOC101105685	0.190116051	0.598829534	0.805881822	0.004878888
105604809	-	0.361849317	0.304207256	0.805880605	0.004879003
101116157	ASGR1	0.545570663	0.102844273	0.80555349	0.004909899
106991637	-	0.163119466	0.652519908	0.805191387	0.004944262
101113105	POC1A	0.469646554	0.170835349	0.804772632	0.004984215
101111028	ZC2HC1A	-0.301085892	0.397903368	-0.868347067	0.001117853
101113048	STK16	0.13422639	0.711612906	0.804566376	0.005003978
101120923	LOC101120923	0.613349689	0.059317464	0.804494354	0.005010893
101101836	-	0.102613482	0.777881644	0.80390166	0.005068051
101101890	TRIR	0.295362822	0.40737051	0.803655212	0.005091955
101120455	LOC101120455	0.257403706	0.472776862	0.802743073	0.005181126
101106377	PDZK1IP1	0.299668138	0.400238701	0.80271449	0.005183938
105603226	LOC105603226	0.374819395	0.285887435	0.802564748	0.005198688
101107246	AGPAT3	0.49967981	0.141408958	0.801662091	0.005288239

105605833	LOC105605833	0.177076736	0.624563816	0.80162378	0.005292064
106991117	LOC106991117	0.458497961	0.182616183	0.801240744	0.005330414
554256	TLR5	0.160340664	0.658133905	0.801102061	0.005344348
101120358	SLC25A1	0.385747439	0.27092612	0.800509117	0.005404216
105611280	LOC105611280	0.518463882	0.124700283	0.800204264	0.005435181
101102503	CACNA1E	0.326713023	0.356840127	0.799988653	0.005457158
101118057	-	0.515348903	0.12738176	0.798991086	0.005559664
101116029	CRYBG2	0.365676588	0.298738229	0.798502516	0.005610365
101113116	NEURL1	0.303830116	0.393401743	0.798475762	0.005613151
101105541	ECM1	0.451851653	0.189861308	0.798361038	0.005625108
101104157	-	0.513588467	0.128912851	0.798215342	0.005640319
101101932	FZD4	0.295510133	0.407125496	0.591242628	0.071834258
101110159	CHMP6	0.166174921	0.646365078	0.798010279	0.005661778
101122041	MRC2	0.249562116	0.48683156	0.797913197	0.005671958
105603854	-	0.524845469	0.119316933	0.79785454	0.005678115
101114323	PARP14	-0.295184012	0.407668009	-0.750531093	0.012380854
101112958	HPGD	0.292950329	0.411393039	0.797544721	0.005710713
101103349	TRIM2	0.490430956	0.150113437	0.796487736	0.00582293
101114310	LOC101114310	0.152031358	0.675012225	0.796468539	0.005824983
105605818	LOC105605818	0.581580893	0.077804232	0.795110599	0.005971481
101108719	PAX5	0.632844343	0.049552076	0.795011732	0.005982248
101123476	ATP6V0A1	0.132296889	0.71561279	0.794539706	0.006033846
101111957	CACFD1	0.218248262	0.54467991	0.794158961	0.006075696
101112689	KAZALD1	0.346161371	0.327171334	0.793553414	0.00614268
101116992	LIPC	0.292852815	0.411556026	0.648221616	0.042652267
105613348	LOC105613348	0.454211692	0.187269642	0.793258338	0.00617551
101121302	MARK2	0.225957955	0.530189875	0.792289458	0.006284185
101117681	SCRN2	0.308471966	0.385843643	0.791898574	0.006328411
101115920	SELENBP1	0.402698602	0.248586651	0.791810015	0.006338461
101118975	SMOX	0.405195539	0.245385823	0.791777325	0.006342174
105604623	-	-0.045185558	0.901358157	0.790921063	0.006439978
105612542	LOC105612542	0.501461225	0.139768727	0.790408511	0.006499033
101120483	NINJ2	0.289885086	0.416530914	0.66041567	0.037662988

101119929	ZNF584	0.335582443	0.343146837	0.790194615	0.006523791
101102434	ME3	0.463266724	0.177519937	0.790160652	0.006527729
101109427	ASXL2	-0.28880683	0.418345396	-0.797508225	0.005714562
101106393	ALAD	0.165338901	0.648047236	0.789692377	0.006582187
101119773	LOC101119773	-0.286744388	0.421826369	-0.357766342	0.310099594
101103461	LOC101103461	0.067225063	0.853607947	0.789346913	0.00662257
101111742	TIMM8B	0.682960858	0.029505489	0.789106443	0.006650783
101117867	SAE1	0.667285672	0.03503354	0.789010974	0.006662008
101103449	-	0.287803169	0.420037679	0.788924293	0.006672211
106991310	-	0.563219318	0.090014119	0.788760126	0.006691565
105615960	LOC105615960	0.83486254	0.002652145	0.788200967	0.006757785
101103093	ISCU	0.362592638	0.30314096	0.787248893	0.006871605
101107390	RPP25	0.173050077	0.632586819	0.786596712	0.006950354
101120299	COLEC11	0.283380989	0.427531983	0.64578518	0.043699637
780507	FGFR1	0.281368588	0.430962822	0.669044165	0.03438105
101121949	LOC101121949	0.266032622	0.457520471	0.786207544	0.006997649
105609948	LOC105609948	0.280198753	0.432963045	0.619688011	0.056014476
101111275	GRB10	0.27986318	0.433537609	0.6931204	0.0262597
101114545	WFDC3	0.42403774	0.221980793	0.786177634	0.007001293
101106801	PLXND1	0.42403774	0.221980793	0.786177634	0.007001293
101105192	TREML1	0.278689464	0.435549991	0.666715841	0.035246762
105605623	-	0.42403774	0.221980793	0.786177634	0.007001293
105614828	-	0.277470769	0.437644027	0.344016024	0.330379417
106991509	-	0.42403774	0.221980793	0.786177634	0.007001293
101120987	LZTS1	0.42403774	0.221980793	0.786177634	0.007001293
105615065	-	0.42403774	0.221980793	0.786177634	0.007001293
105603595	LOC105603595	0.42403774	0.221980793	0.786177634	0.007001293
101108705	LOC101108705	0.42403774	0.221980793	0.786177634	0.007001293
105602874	-	0.42403774	0.221980793	0.786177634	0.007001293
105614872	CHADL	0.42403774	0.221980793	0.786177634	0.007001293
105602321	LOC105602321	0.42403774	0.221980793	0.786177634	0.007001293
101123155	MON1A	0.250840063	0.484528875	0.785822039	0.007044723
105604156	LOC105604156	0.30919528	0.384672321	0.784654423	0.007188672

101119179	SLC44A3	0.273735458	0.444090916	0.52437666	0.119707385
101106228	ZCWPW1	0.308928841	0.385103586	0.784219778	0.007242786
105608780	-	0.713265894	0.020563395	0.783623696	0.007317468
101116984	-	0.272590522	0.446075604	0.3780043	0.281482059
101102995	SHBG	0.368661793	0.294509096	0.783580714	0.007322874
101110433	CUBN	0.488256059	0.152206383	0.783140719	0.007378378
101103488	LOC101103488	0.225840621	0.530409222	0.783094215	0.007384262
100302349	POU5F1	0.188505627	0.601986991	0.782353	0.007478491
101110945	PDCD7	-0.270452627	0.449792285	-0.641421333	0.045618378
106990181	-	0.137252625	0.705352249	0.78195385	0.007529585
101120752	TMEM260	-0.268598426	0.45302706	-0.763631409	0.010151499
101109258	C3H12orf50	0.621571644	0.055056872	0.780327792	0.007740291
101110092	CPEB1	0.621571644	0.055056872	0.780327792	0.007740291
101123372	LOC101123372	0.488109158	0.152348382	0.780007202	0.007782321
101106861	MRPL27	0.311557171	0.380859625	0.779611257	0.007834452
101112641	ZBED4	0.334197633	0.345266931	0.778593026	0.007969651
101103220	-	0.304120486	0.392926866	0.778212669	0.008020575
101102666	TULP2	0.348897329	0.32310358	0.776745714	0.008219136
497274	CRYAB	0.267501009	0.454946494	0.573288023	0.083177028
101110931	VWF	0.347333171	0.325425901	0.776206867	0.008292937
101118332	PLAT	0.295825661	0.40660093	0.775844365	0.008342849
101115175	PGP	0.600661538	0.066309353	0.775471435	0.008394417
101110941	LOC101110941	-0.266409438	0.45685932	-0.613807238	0.059074837
101119755	SERGEF	0.111918658	0.758221547	0.775408221	0.008403181
101108095	LRRC58	0.259880542	0.468375012	0.775008649	0.008458724
101102949	COLEC12	0.681828677	0.029883332	0.774917837	0.008471384
101103995	MIEN1	0.295265325	0.40753271	0.773954174	0.008606549
106991842	-	0.400271442	0.251720201	0.773329305	0.008695004
101117928	HTR4	0.264219039	0.460708523	0.536799648	0.109628221
106991655	-	0.38729277	0.268845709	0.772984632	0.008744069
105605032	LOC105605032	0.263325595	0.462282717	0.64844718	0.042556162
101104397	GPX3	0.3922448	0.262238146	0.772931414	0.008751663
101105117	TARS2	0.537286397	0.109244572	0.772310285	0.00884063

101117638	LOC101117638	-0.263001904	0.462853631	-0.535093478	0.110979679
101113914	VSIG1	0.262947464	0.462949681	0.299990832	0.399706582
101106615	FXYD3	0.311241813	0.381367619	0.771349332	0.008979527
101123118	LOC101123118	0.28890791	0.418175142	0.771218138	0.008998609
105616808	-	0.105092447	0.77263249	0.771098859	0.009015982
101117219	RUSC2	0.523973575	0.120043735	0.771092205	0.009016952
105603274	-	0.262291486	0.464107738	0.697604269	0.024908403
443047	AQP3	0.210678508	0.559057204	0.770738323	0.00906864
105613005	-	0.261959592	0.46469415	0.437259304	0.206350432
101101926	VOPP1	0.467167209	0.173415009	0.769872616	0.009195966
101110235	MLLT1	0.490041443	0.150486981	0.769438929	0.009260223
105608396	-	0.261110047	0.466196677	0.606863085	0.062828033
100302063	RAB8A	0.260931351	0.466512997	0.615731402	0.05806166
101114004	SLC4A8	-0.260854088	0.466649793	-0.60737754	0.062544751
101113630	PHF1	0.333295407	0.346651778	0.769299172	0.009280998
101103364	SERPINB5	0.356861933	0.311412852	0.767750529	0.009513405
101102363	POP7	0.604849462	0.063944925	0.767708935	0.009519703
101114733	-	0.393599909	0.260445742	0.7672648	0.009587136
101113185	ACKR1	0.435568045	0.208313241	0.766986406	0.009629576
101118156	LOC101118156	0.038083586	0.916812876	0.766851243	0.009650229
101114928	ZNF488	0.195544088	0.588231662	0.7667971	0.009658511
101109659	ROR1	0.573062823	0.083326169	0.766081579	0.009768428
105608589	LOC105608589	0.319368626	0.36838284	0.765960025	0.009787188
101111280	KDM3B	-0.257888101	0.471914566	-0.740848634	0.014234462
101101911	CTSZ	0.349519786	0.322181815	0.765898138	0.009796749
105608992	MSANTD3	0.257544138	0.472526801	0.596860188	0.068504428
101111652	CDH3	0.607775905	0.06232597	0.765734172	0.009822113
101102757	LMO1	-0.037727584	0.917588279	0.765409475	0.009872476
101112155	LOC101112155	0.520454315	0.123005344	0.765228059	0.009900694
494434	UCP1	0.451080024	0.190713207	0.764811824	0.009965651
101105290	LOC101105290	0.510534115	0.131596167	0.764538793	0.010008423
101103184	CARD6	-0.254955748	0.477145146	-0.6766881	0.031640411
101104226	C3AR1	0.592655738	0.070986924	0.764221182	0.010058341

100270719	UQCRH	0.629435113	0.051176379	0.764009676	0.01009168
105602135	-	0.254255459	0.478398015	0.570819497	0.084821235
101115498	AS3MT	-0.253333406	0.480049818	-0.762010429	0.010410667
101110647	LOC101110647	0.252455326	0.481625148	-0.584082871	0.076228544
105604727	LOC105604727	0.25209469	0.482272801	0.574367367	0.08246461
100217407	TIMP3	0.446910782	0.195354852	0.763696381	0.010141206
101111014	-	0.351790686	0.318830577	0.763486774	0.010174437
101102095	JAGN1	0.452595788	0.189041881	0.763418174	0.010185329
105607129	AOPEP	0.434688146	0.209338664	0.762303593	0.010363454
101108988	ERAS	0.382504831	0.275319962	0.762125952	0.010392045
105602955	LOC105602955	0.159804064	0.65921978	0.761854743	0.010435801
101111441	ESYT1	0.378796799	0.280391612	0.761433736	0.010503983
101118646	LY6G6C	0.425285332	0.220477907	0.760780837	0.010610339
101103777	ZNF668	0.534488796	0.111461147	0.760088402	0.01072396
101113262	LRRC36	0.248053899	0.489555213	0.435048752	0.208918066
100135685	ASRGL1	0.575623562	0.081640426	0.760025626	0.010734303
101110167	SPINT2	0.311277561	0.381310018	0.759857362	0.010762061
101122618	NNAT	0.438504271	0.204912434	0.759404379	0.010837039
101106327	NDFIP1	-0.247353765	0.490821785	-0.607676704	0.062380404
101118655	DSG2	0.247189089	0.491119896	0.431639852	0.212913571
101104217	-	0.446048563	0.196322914	0.759062662	0.010893842
105602228	LOC105602228	0.155055456	0.668853952	0.759043972	0.010896955
101116710	SIGMAR1	0.399048604	0.253307199	0.758772408	0.010942255
443408	LPL	0.713142934	0.0205953	0.758682618	0.010957262
101107537	PTGES3L	0.244939948	0.495199222	0.629969253	0.050919586
101116073	ATF3	-0.243606082	0.497625284	-0.643762892	0.044581955
101123297	BRD4	0.512317581	0.130025207	0.758568728	0.010976317
101117390	ALOX5	0.267164078	0.455536534	0.758430281	0.010999513
101106977	LOC101106977	0.241819859	0.500881983	0.662444935	0.036872893
101111729	KCNK2	0.504887253	0.136647108	0.757808386	0.011104133
101111482	VPS9D1	0.241426462	0.501600443	0.696907409	0.025115209
101116914	JMJD7	0.554159926	0.096464257	0.75742223	0.011169446
101103834	FTL	0.219868558	0.541621672	0.757189841	0.011208881

101105696	PPP1R10	0.403463151	0.247604116	0.756377712	0.011347463
101117395	LOC101117395	0.240210096	0.503824644	-0.602506792	0.065260622
101112604	DUSP7	0.546586356	0.102076297	0.756021779	0.011408577
101116493	SRGAP2	0.532274178	0.113235679	0.755789765	0.011448538
101123651	MTARC1	0.610554239	0.060814094	0.755380343	0.011519295
101104401	LOC101104401	0.238994079	0.506052347	0.492011502	0.148603466
105608949	LOC105608949	0.14647735	0.686367537	0.754995944	0.011586006
101105426	GPRC5C	0.148844381	0.681521024	0.754372618	0.011694758
101108423	LOC101108423	0.517962602	0.125129415	0.754034839	0.011753989
101116540	IDS	-0.237515394	0.508766803	-0.590563602	0.072243749
101109777	TMBIM1	0.065850804	0.856574384	0.753961418	0.011766891
101115699	DUSP2	0.218971059	0.543314817	0.753588186	0.011832634
101123091	NUDT2	0.126455962	0.727758801	0.753065948	0.011925054
101106017	MYO5C	-0.234884201	0.513611935	-0.395377033	0.258105409
105605297	LOC105605297	0.192665646	0.593843016	0.752895117	0.011955396
105607815	LOC105607815	0.597644024	0.068047977	0.752555041	0.012015959
101110988	KCNAB1	0.234673397	0.514000939	0.575294372	0.08185589
101115412	FLI1	-0.233560663	0.516056335	-0.838014756	0.002465264
101122992	TRPT1	0.351759826	0.318875996	0.752360269	0.012050742
101111588	PTPRF	0.20699302	0.566109712	0.752216415	0.012076477
101119661	DIDO1	-0.231793078	0.519328317	-0.700185627	0.024152521
101107544	HCST	0.200573388	0.578474686	0.751968901	0.012120847
101117116	TXNRD3	0.569313415	0.085834575	0.749845457	0.012506207
105611737	LOC105611737	-0.231003693	0.5207923	-0.491735869	0.148866124
101118623	KCNRG	0.468726156	0.171790299	0.749118199	0.012640138
101104240	TMOD2	0.765173107	0.009909253	0.748866911	0.012686648
101122536	EFCAB5	0.229056043	0.524411623	0.531108137	0.114177076
101113571	LOC101113571	-0.228995757	0.524523816	-0.592733879	0.07094026
101105535	TMEM43	0.571883031	0.084110309	0.748665018	0.012724102
105609643	GATAD1	0.228856404	0.524783192	0.47111722	0.169316058
101120219	LOC101120219	0.369594047	0.293194965	0.747190052	0.013000083
101122943	TAB1	0.580490961	0.078497185	0.745867502	0.013251084
101110591	SEC24D	-0.2260355	0.530044932	-0.566921526	0.087459834

105602998	-	0.096392454	0.791089802	0.745649088	0.013292859
105605986	-	0.520707798	0.122790526	0.744686931	0.013477986
105605723	-	0.101479288	0.780285988	0.743864618	0.013637628
101119100	ZCCHC2	-0.225171077	0.531661577	-0.343904567	0.330546529
105603614	AMIGO3	-0.224552097	0.532820416	-0.482368288	0.157960584
101115782	PMPCA	0.224393004	0.533118431	0.661095333	0.0373971
101123423	DNAJC4	0.361108942	0.305271297	0.743589071	0.013691416
101111630	SH3RF2	0.223405133	0.534970427	-0.509099295	0.13286848
101112079	FBXO2	0.480594423	0.159719522	0.743114922	0.01378432
101102637	SLC39A5	0.448337496	0.193759132	0.74248633	0.013908162
101117200	RNF123	0.222925229	0.53587105	0.616131719	0.057852323
106991594	LOC106991594	0.408452523	0.241245508	0.74238893	0.013927421
101110592	C6H4orf19	0.222008229	0.537593655	0.470835671	0.169606288
101120881	RMC1	0.797532683	0.005711982	0.742232169	0.013958456
101115246	TRIM8	0.449255187	0.192736775	0.742115613	0.013981562
101112559	LOC101112559	0.525417531	0.118841561	0.74172524	0.014059147
105614926	SYNGR1	0.53349064	0.112258775	0.741719671	0.014060256
101108903	CNPY2	0.179866293	0.619026393	0.741344978	0.01413501
101113728	LOC101113728	0.32725562	0.355994657	0.739918292	0.014422188
101105467	SETD1A	0.18190134	0.614997551	0.739745988	0.014457144
106991487	LOC106991487	-0.219389831	0.542524535	-0.7080802	0.021938833
101120918	LOC101120918	0.663650487	0.036408854	0.739737702	0.014458826
101101915	DHX34	0.395687032	0.25769836	0.739136923	0.014581183
101109893	GRB2	0.195744261	0.587842164	0.738303883	0.014752035
101110294	LRRC71	0.006702299	0.985339381	0.737991728	0.014816414
101103693	GNAI3	-0.215973633	0.548984673	-0.711183041	0.021108467
105602959	LOC105602959	0.302556509	0.3954879	0.737410595	0.01493679
106990491	LOC106990491	-0.212167467	0.556217649	-0.491711995	0.148888888
101120889	LOC101120889	-0.211879287	0.556766791	-0.703949795	0.023078756
105607844	LOC105607844	0.145631922	0.688101066	0.737406455	0.01493765
105606871	LOC105606871	0.210545293	0.559311528	0.655421705	0.039655867
101122352	GIPC1	0.044229165	0.903437745	0.737307902	0.014958132
101114901	FOXR1	0.671354621	0.033536341	0.737036469	0.015014646

105609911	PTRHD1	0.83478936	0.002656601	0.736823055	0.015059184
101109667	GTF3C1	0.210355687	0.559673586	0.735778498	0.015278506
105602665	-	0.276433819	0.439429406	0.735319952	0.015375485
101111402	SRF	0.262522949	0.46369897	0.734276773	0.015597705
101117231	-	0.162341282	0.6540905	0.733899113	0.015678703
101121036	LOC101121036	0.131894343	0.716448062	0.73349834	0.015764979
105611619	CENPV	0.294827071	0.408262187	0.733238365	0.015821121
101122165	CCDC171	-0.205740809	0.56851365	-0.626467567	0.052618731
101115850	LGALS8	-0.204731153	0.570454768	-0.609013794	0.061649337
101112898	RBM27	-0.20106638	0.577521549	-0.61870281	0.056519693
101111287	SPINT1	0.247125493	0.491235042	0.732172178	0.016052822
106991991	LOC106991991	0.200411613	0.578787585	0.351580227	0.31914039
101123215	CDH24	0.554650912	0.096107371	0.731514933	0.016196823
101121909	NYAP1	0.468613711	0.171907183	0.731099344	0.01628834
101105254	ZNF410	-0.196500668	0.586371207	-0.592284496	0.071208896
101120347	ANGPTL1	0.468613711	0.171907183	0.731099344	0.01628834
101104172	LOC101104172	0.468613711	0.171907183	0.731099344	0.01628834
101111582	SCN5A	0.468613711	0.171907183	0.731099344	0.01628834
101102994	LOC101102994	0.19553322	0.588252811	0.444130896	0.198486001
101102354	NIT1	0.194493605	0.59027725	0.586984389	0.074427314
101111870	KANSL3	-0.194189221	0.59087046	-0.568404674	0.086449749
105606111	LOC105606111	0.468613711	0.171907183	0.731099344	0.01628834
101108448	FBXL18	0.468613711	0.171907183	0.731099344	0.01628834
101120708	LRRC42	-0.192537821	0.594092656	-0.451071218	0.190722942
101120828	SPPL2B	0.468613711	0.171907183	0.731099344	0.01628834
443345	GJB2	0.468613711	0.171907183	0.731099344	0.01628834
101119597	LOC101119597	-0.190430031	0.598214634	-0.609627123	0.061315886
105609173	-	0.468613711	0.171907183	0.731099344	0.01628834
101120765	KLHL21	0.468613711	0.171907183	0.731099344	0.01628834
106991534	-	0.468613711	0.171907183	0.731099344	0.01628834
101101814	-	0.468613711	0.171907183	0.731099344	0.01628834
101114396	RBM15B	0.183547072	0.611746205	0.681123625	0.030120287
101112000	LOC101112000	0.468613711	0.171907183	0.731099344	0.01628834

678671	CCR10	0.468613711	0.171907183	0.731099344	0.01628834
101111886	IFT43	0.347447116	0.325256434	0.730990498	0.016312369
101111283	CATSPERD	0.240921632	0.502523054	0.730775457	0.016359913
105614894	NSMF	0.179497272	0.619757938	0.606582119	0.062983101
105612560	LOC105612560	0.179497272	0.619757938	0.606582119	0.062983101
105603577	-	0.179353749	0.620042538	-0.593860119	0.07026991
101112751	DYNLRB1	0.522310418	0.121437758	0.730691587	0.016378482
105616126	LOC105616126	0.177813019	0.623100595	0.662654791	0.03679183
105612581	LOC105612581	0.054412629	0.881324159	0.730558166	0.016408052
106991531	-	0.248231813	0.489233581	0.72977313	0.016582792
101113129	WNT10A	0.175456563	0.627787753	-0.391803282	0.262823603
101121769	LOC101121769	0.174624227	0.629446205	0.55494012	0.095897546
101118974	TP73	-0.174407629	0.629878028	-0.363205479	0.30226333
101112370	NDUFB10	0.33979072	0.336744993	0.728653872	0.016834158
101104075	WFIKKN2	0.41473244	0.233373897	0.728379921	0.016896084
106991083	-	0.375608145	0.284792993	0.728278535	0.016919042
100302338	EIF6	0.2083602	0.563489522	0.728213841	0.016933702
100037663	GSTM3	0.165734704	0.64725066	0.426088762	0.219513158
101116661	LOC101116661	0.413757039	0.234586877	0.727645714	0.017062829
101122475	LOC101122475	0.163657175	0.651435378	0.683601444	0.029293156
101109844	CNNM2	-0.003612403	0.992097971	0.727615212	0.017069781
101123619	ATF7	0.163267787	0.652220694	0.646663483	0.043320109
101111499	AQP11	0.300669575	0.398588448	0.726969612	0.017217386
101119664	IRF2BP1	0.113211856	0.755498854	0.726418428	0.017344104
101123666	FHDC1	0.312463354	0.379401745	0.725454381	0.017567294
101108788	CLCF1	0.161919048	0.654943198	0.598279825	0.067679195
105612547	LOC105612547	0.161406604	0.655978557	0.417696987	0.229709074
101110076	PRG4	0.496337135	0.144518345	0.725373525	0.017586104
101105355	SLC37A4	0.586803903	0.074538544	0.724835572	0.017711603
105602840	LOC105602840	-0.15953771	0.659758993	-0.590086222	0.072532544
105603608	-	0.159284257	0.660272219	0.561135799	0.091472367
101107427	CD207	0.159284257	0.660272219	0.561135799	0.091472367
101106270	ARHGAP39	0.159284257	0.660272219	0.561135799	0.091472367

105608892	-	0.159284257	0.660272219	0.561135799	0.091472367
101105165	CNKSR3	0.158891157	0.661068473	0.437947824	0.205554438
101116612	HSD17B8	0.07971997	0.826716626	0.723874235	0.017937419
106990184	LOC106990184	0.490671238	0.149883286	0.723638703	0.017993048
101105123	LOC101105123	-0.155734535	0.667473503	-0.730312271	0.016462647
101112692	YBEY	0.228511144	0.525426045	0.723389677	0.018051995
101116330	PELP1	0.085557071	0.814207885	0.72277346	0.018198433
105606707	LOC105606707	0.526037587	0.118327643	0.72246342	0.018272422
101103380	LOC101103380	0.152982201	0.673074036	0.455915915	0.185411163
101107232	LOC101107232	0.448704544	0.193349841	0.722381762	0.018291944
101108855	LYPD5	0.382347279	0.275534431	0.721945662	0.018396445
105611808	-	0.151849886	0.675382331	0.486099815	0.154298736
101115479	CADM1	-0.150393425	0.678355	-0.610975387	0.060587047
101109377	LOC101109377	0.149454963	0.680272546	0.572210774	0.083892003
101115053	UBE2C	0.38480205	0.272203175	0.720292753	0.018796287
678676	CAPNS1	0.148292867	0.682649323	0.664710549	0.036004093
780456	ACSS1	0.606289193	0.063145037	0.720031994	0.01885991
101120526	GLRX5	0.509576192	0.132444759	0.719989365	0.018870325
105606990	-	0.145503247	0.688365027	0.546176848	0.102385492
101108219	LOC101108219	0.144809031	0.689789639	0.341623093	0.333976784
101118757	LOC101118757	0.144714756	0.68998317	0.374361702	0.28652355
101101946	TMEM265	0.241466276	0.501527712	0.71912849	0.019081511
101104787	LOC101104787	-0.143627838	0.692215603	-0.629762058	0.051019095
101117658	TMEM52B	-0.140268564	0.699128691	0.479284196	0.161026259
105608454	LOC105608454	0.139861937	0.699966857	0.514682792	0.127959761
105616290	LOC105616290	0.176540379	0.625630467	0.718959324	0.019123202
101114114	RRAGD	-0.135454323	0.709070672	-0.596481404	0.06872572
101119115	-	-0.135136196	0.709729055	0.422029232	0.224412542
101119713	-	-0.134394656	0.711264387	0.588028622	0.073785892
101108377	EMG1	0.585832757	0.075138891	0.71696484	0.019619502
105601981	LOC105601981	0.668388436	0.034623388	0.715519046	0.019984791
101115653	LOC101115653	0.131973047	0.716284732	0.60769788	0.062368782
106991621	P2RY2	0.600513411	0.066394014	0.715135227	0.020082549

101103515	DAG1	0.129962055	0.720461269	0.63112997	0.05036452
101117691	LOC101117691	0.267038877	0.455755875	0.715098135	0.020092014
101107589	GRAP2	0.466037999	0.174597541	0.715024585	0.020110791
101107250	MBLAC1	0.123619217	0.733677645	0.550994316	0.098785501
101122601	LOC101122601	0.57323445	0.083212492	0.71328498	0.020558446
101113644	PDXDC1	0.121702772	0.737683473	-0.671409338	0.033516509
101121321	ELF4	0.120415876	0.740376594	0.685044443	0.028818673
105613835	-	0.120001639	0.74124402	0.364706313	0.300119732
101118252	-	0.119849215	0.741563267	0.580155746	0.078711106
106991184	-	0.119785396	0.741696946	0.650261167	0.041788569
101114040	SFMBT2	0.118735872	0.743896204	0.673148974	0.032890116
105604629	LOC105604629	-0.116121944	0.749380863	-0.737742885	0.014867876
106990804	-	0.114865869	0.752020034	0.643506408	0.044694704
105602642	-	0.085360838	0.814627809	0.71088882	0.021186258
101112473	REXO4	0.591461091	0.071702835	0.710812305	0.02120652
105613124	-	0.110416434	0.761387327	0.65021495	0.04180801
101116007	COL17A1	0.110327305	0.761575257	-0.522579388	0.121211634
105616451	LOC105616451	-0.108789081	0.764820405	-0.538369321	0.10839406
105604515	C23H18orf32	-0.108705699	0.764996407	-0.839478553	0.002381794
105604284	LOC105604284	0.107731559	0.767053344	0.665324148	0.0357712
101121149	LOC101121149	-0.106043542	0.770620757	-0.599169043	0.067165618
101120826	MAPK9	-0.105607176	0.771543597	-0.756248717	0.011369585
101121439	MIF4GD	0.626243551	0.052728695	0.710538387	0.021279169
105601908	ZNF248	-0.104523728	0.773836014	-0.77426685	0.008562527
101117505	PDE4A	0.191274671	0.596561619	0.708336876	0.021869302
105611784	LOC105611784	0.207169027	0.565772135	0.708095657	0.021934641
105603563	-	0.098227011	0.787189637	0.649221105	0.042227527
101108271	SETBP1	-0.097135965	0.789508632	-0.634238473	0.048897866
101114865	MISP	0.309641302	0.383950907	0.708084023	0.021937796
100302317	ARL8B	-0.096334873	0.791212283	-0.596382422	0.068783623
101122169	RAB33A	0.095852445	0.792238629	0.639254529	0.046591692
101115912	TRAK1	-0.095170146	0.793690683	-0.562648134	0.090412398
105604729	-	0.094991154	0.794071705	0.564990305	0.088786375

101118891	CACNG6	0.09466113	0.794774332	0.565113304	0.088701506
106991845	LOC106991845	-0.094061516	0.796051261	-0.669281714	0.034293544
101102311	TBCK	-0.093986961	0.796210063	-0.756800936	0.011275095
101114422	TSSK3	0.093353509	0.797559585	0.348972548	0.32299212
101113303	CDHR1	0.09275042	0.798844868	0.566175864	0.087970507
101105706	FAM204A	0.090675207	0.803270803	-0.593189209	0.070668743
101107532	LOC101107532	-0.089880971	0.80496606	-0.316773987	0.372504367
101117994	LOC101117994	0.089274892	0.806260198	0.637189498	0.04753211
780463	LOC780463	0.089274892	0.806260198	0.637189498	0.04753211
101103064	RBM22	-0.088871725	0.807121301	-0.778888759	0.007930216
105606624	-	0.088841461	0.807185947	0.652267644	0.040950418
105605118	LOC105605118	0.088841461	0.807185947	0.652267644	0.040950418
101103235	PRDM6	0.309641302	0.383950907	0.708084023	0.021937796
105609820	LOC105609820	0.086971802	0.811181738	0.405990632	0.244371453
105606960	TRNP1	-0.086649498	0.811870958	0.660999264	0.037434606
101115884	SLC10A5	-0.086374039	0.812460096	-0.749086116	0.01264607
105606122	IQSEC1	0.309641302	0.383950907	0.708084023	0.021937796
101112817	PTPRS	0.309641302	0.383950907	0.708084023	0.021937796
554261	TLR1	-0.084774634	0.815882492	-0.677741523	0.031274767
101107853	CCDC184	-0.083185189	0.819286355	0.491122833	0.149451312
101119745	LOC101119745	-0.081189687	0.823563649	0.616444968	0.057688863
100913157	ILK	0.364965689	0.299750094	0.706894173	0.022262092
101104176	LOC101104176	-0.078898929	0.828478976	0.381851101	0.276210451
105608797	LOC105608797	0.078061611	0.830276972	0.678667544	0.030955728
105602485	-	0.078061611	0.830276972	0.678667544	0.030955728
101111323	STAB1	0.078061611	0.830276972	0.678667544	0.030955728
105606082	-	0.078061611	0.830276972	0.678667544	0.030955728
101113356	RINL	-0.074018365	0.838969002	-0.78930307	0.006627708
101107048	VSIG10	-0.073635653	0.83979257	-0.688483176	0.027709163
101123271	ACADL	0.073231032	0.840663437	0.359690646	0.307315105
105605926	LOC105605926	-0.07253573	0.8421603	0.698463357	0.024655065
105602696	LOC105602696	0.070498138	0.846549485	0.63002877	0.050891026
101122904	LOC101122904	0.069250566	0.84923877	0.568429714	0.086432761

101122241	HPS3	0.069156102	0.849442456	-0.772883261	0.008758537
101117599	DVL2	-0.069065296	0.849638261	-0.392857318	0.261427129
105609819	-	0.223036041	0.535663039	0.706610276	0.022339953
101102978	CCDC130	0.441896761	0.201023478	0.706576635	0.022349192
101102474	GGCT	0.063944616	0.860691703	-0.593917328	0.070235969
105616771	-	0.471489936	0.168932305	0.706027236	0.022500446
101108245	DEF8	-0.061165108	0.866700767	-0.431078881	0.213575251
101119485	C11H17orf67	-0.060542997	0.868046573	0.398451107	0.254084643
101108368	PSMD14	0.057551589	0.874522055	-0.697627976	0.024901388
101107746	RAB13	0.055500836	0.878965209	0.480574704	0.15973914
101116931	RPS16	0.216664687	0.54767543	0.705685997	0.022594746
101118346	PUS10	0.053721196	0.882823442	-0.7445542	0.013503665
101116936	BICDL1	0.496938831	0.143955593	0.705550841	0.022632171
101121560	ATG3	0.052553313	0.885356606	-0.661874694	0.037093774
105604766	LOC105604766	0.050436261	0.889950909	0.438291749	0.205157495
100500777	MAPRE1	0.049573568	0.891823928	-0.568779543	0.086195639
101108383	KRBA1	0.046601319	0.898280709	0.678407736	0.031045014
101117042	HIP1	0.55395093	0.096616426	0.70519823	0.022730011
101106867	BPIFB6	0.239721721	0.504718836	0.704667475	0.022877829
105601868	ARPC4	-0.045171266	0.901389231	0.676319517	0.03176903
101120954	RHBDD3	0.25689159	0.473689257	0.704581656	0.022901791
105605809	LOC105605809	0.330955547	0.350256382	0.704151457	0.023022175
105613831	LOC105613831	0.042314323	0.907602974	0.509225311	0.132756434
101109027	LOC101109027	-0.041242613	0.909935085	-0.533620494	0.112154807
101102507	CRISPLD2	-0.040400104	0.911768874	0.678659093	0.03095863
101117702	MCEE	0.039012795	0.914789281	-0.60042715	0.066443349
101122279	RUFY3	0.038873895	0.915091745	-0.701900013	0.023659312
101120656	LRP1	-0.128738486	0.723005713	0.702982583	0.023351462
101113111	LOC101113111	0.50997517	0.132090909	0.702113715	0.023598321
101111303	LOC101111303	0.259193666	0.469593914	0.702107263	0.023600161
105610080	LOC105610080	0.036430612	0.920413717	0.577337709	0.080524379
101114640	TRIM21	-0.035605873	0.922210823	-0.720745733	0.018686117
100302066	RAB4A	0.035317492	0.922839279	0.491258874	0.149321331

101102689	SLC16A12	0.035110033	0.92329141	-0.605699147	0.063472056
106991757	ARIH2OS	0.032964649	0.927968138	0.62118657	0.055251748
101114808	HARBI1	-0.031690598	0.930746397	-0.711574695	0.021005221
101107328	SLC29A3	-0.030652249	0.933011168	-0.675853795	0.031932053
105615270	LOC105615270	0.028860313	0.936920623	0.611045437	0.060549337
101122777	YBX3	-0.02858923	0.93751215	-0.516485456	0.126399281
101109079	LOC101109079	-0.026804564	0.941407126	-0.623388232	0.054143676
105609438	LOC105609438	-0.024263659	0.946954482	-0.722655835	0.018226479
101107192	TRIM52	0.022316205	0.951207605	-0.626720867	0.052494576
101107062	TYW3	0.022016177	0.951862949	-0.764169111	0.010066542
105609069	-	0.021948836	0.952010046	0.497276423	0.143640438
101118173	TBC1D13	0.711621126	0.020993004	0.70197438	0.023638075
101121345	PEAK3	0.020361518	0.95547764	0.668992049	0.034400269
106991113	-	0.019983277	0.956304034	0.491182178	0.149394602
101113031	CCDC58	0.018400402	0.959762745	-0.759558003	0.01081157
101106433	THBS1	-0.016639764	0.963610593	0.361336626	0.304943868
101103179	TRIM6	0.012018215	0.973713951	-0.437544218	0.206020829
443193	CPT1B	0.010421485	0.977205478	0.587892113	0.073869539
101104589	GRB7	-0.0092811	0.979699343	0.5487043	0.100486599
106991913	-	-0.00845612	0.981503559	0.53782832	0.108818431
101115356	SOX5	-0.00789749	0.982725318	-0.675590331	0.032024529
101116080	LOC101116080	-0.007544717	0.983496871	-0.574892386	0.082119498
101110963	PVR	-0.007257444	0.984125177	0.523630276	0.12033066
101122510	FHL2	0.155623118	0.667699933	0.701208346	0.023857455
101118748	DUSP5	0.00532751	0.988346403	0.591457014	0.071705286
101113188	LOC101113188	0.454491406	0.186963862	0.700561732	0.02404372
101117567	TRIM32	-0.002389171	0.994773718	-0.299866545	0.39991149
101119041	LOC101119041	0.001595997	0.996508766	0.625480005	0.053104645
100034668	CLN5	0.000178924	0.999608603	-0.81590446	0.003997955
